# Comparison of whole genome sequencing performance from fish swabs and fin clips

**DOI:** 10.1186/s13104-024-07075-1

**Published:** 2025-01-15

**Authors:** Annabell Macphee, Temitope Opeyemi Oriowo, Nils Sternberg, Madlen Stange

**Affiliations:** 1 School of Molecular Biosciences, College of Medical, Veterinary and Life Sciences, University of Glasgow, Glasgow, Scotland; 2 Leibniz Institute for the Analysis of Biodiversity Change, Museum Koenig Bonn, Centre for Molecular Biodiversity Research, Bonn, Germany

**Keywords:** Swabs, Fin clips, Fishes, DNA, Whole-genome sequencing, Illumina, NGS, Sampling, 3Rs

## Abstract

**Objective:**

Fin clipping is the standard DNA sampling technique for whole genome sequencing (WGS) of small fish. The collection of fin clips requires anaesthesia or even euthanisation of the individual. Swabbing may be a less invasive, non-lethal alternative to fin-clipping. Whether skin and gill swabs are comparable to fin clips in terms of DNA extraction quality and sequence read mapping performance from WGS was tested here on Eurasian minnows (*Phoxinus phoxinus*).

**Results:**

Of 49 fin clips, all met the DNA concentration threshold of 20 ng/μl, whereas 43 of 88 swabs met this requirement. Preserving swabs in ATL buffer and treatment with Proteinase K during DNA extraction consistently raised skin swab DNA concentrations above the cut-off. All samples passed the A260/A280 absorbance ratio cut-off of 1.3. Ultimately, 93.88% of the fin clips, 30.61% of the skin, and 7.69% of the gill swabs were suitable for sequencing. Mapping performances of all three tissues were comparable in reads passing quality filtering, percentage of reads mapping to the *P. phoxinus* reference genome, and coverage. Overall, skin swabs treated with Proteinase K during extraction, can match fin clips in WGS performance and represent a viable non-invasive DNA sampling alternative.

**Supplementary Information:**

The online version contains supplementary material available at 10.1186/s13104-024-07075-1.

## Introduction

Modern whole genome sequencing (WGS) techniques allow inspection of genomic diversity, which is highly relevant for the evaluation of conservation status and measures [1, 2]. In fish genetics, genetic material is traditionally gathered from fin clips, i.e. small pieces of fin tissue [3]. Though this sampling approach works well in terms of DNA yield and quality for WGS [4], it often requires euthanisation of the animal if the individual is small. In light of the “3 Rs” (replace, reduce, and refine) defined by Russell and Burch [5], moving towards less invasive and more sustainable sampling techniques would be favourable. Catch-and-release fin clipping presents a non-culling alternative but the animal is released into the wild with missing fin tissue, risking infection, diminished growth, and reduced survival [6]. A non-lethal, less invasive DNA sampling approach as an alternative to fin clipping is represented by mucus swabbing of the lateral length of fishes. The technique is already a research standard on larger fish for genetic sampling [7] and proven appropriate for barcoding in smaller fish such as sticklebacks and zebrafish [8–10]. This begs the question whether swabs could provide qualitatively adequate DNA samples for WGS.

We investigated whether swabbing could replace fin clipping in small stream fishes, here tested on Eurasian minnows, matching DNA quantity and quality for WGS. Additionally, gill swabs and skin swabs were compared to investigate which mucus layer yields superior WGS results. The rationale for this was that external skin swabs may produce lower quality DNA than internal gill swabs [11] and be less subject to contamination from other fishes that might touch the to-be-swabbed fish.

## Methods

### Specimen collection and processing

Swabs and fin clips were collected in April 2024 from Eurasian minnows (*Phoxinus phoxinus*). Fish were caught employing single-pass electrofishing. Fishing was undertaken in minnow quality habitats using DC backpack electrofishing gear (either IG600, Hans Grassl GmbH, Schönau am Königssee or EFGI 650, Elektrofischerei-Eifel UG, Prüm) and a 0.15 m diameter handheld, netted ring anode. Netted fish were sacrificed with 3–6 drops of clove oil (100% *Eugenia Caryophyllus* essential oil) in approx. 80 ml of water. Fish were swabbed and fin clipped after five minutes of absolute inactivity. Prior to taking samples, fish were rinsed with distilled water using a squirt bottle until any visible contaminants (e.g., debris, sand) and oil were removed. Non-invasive swabs from skin mucus and gills were taken from every fish using regular tip size Copan 4N6FLOQSwabs^®^ Genetics swabs. Skin swabs were collected by stroking a swab 10 times along each side of a fish from below the gills to the base of the caudal fin. Gill swabs were sampled by gently lifting the operculum on either side and turning the swab below each operculum 5 times. Swab tips with mucus were broken off the swab stem at the break point following the manufacturers instructions and stored in a locked Eppendorf tube in air. Fish were fin clipped by removing the right pectoral and pelvic fins. Fin clips were stored in 96% molecular grade ethanol. All samples were frozen at – 20 °C once in the laboratory. Fish bodies were preserved in 5–7% formaldehyde solution for analyses unrelated to this study. In total, 39 gill swabs, 49 skin swabs, and 49 fin clips were taken from 49 fish.

To test the effect of storage type before extraction on DNA quality and quantity, skin swabs were either stored separately in empty (n = 39 for gill and skin each) or in with 360 µL ATL filled (AN26 to AN35, n = 10) Eppendorf ^®^ DNA-loBind tubes. The samples stored in ATL buffer were directly subjected to DNA extraction, samples stored in empty Eppendorf ^®^ DNA-loBind tubes were stored at − 20 °C.

### DNA extraction, assessment of quantity and purity

DNA was extracted from all samples following the QIAGEN DNeasy^®^ Blood & Tissue kit including RNase (4 µl) treatment directly after lysis before precipitation. All samples were eluted in 50 to 100 µl AE buffer (for details on each sample see supplementary file 1). To assess the impact of Proteinase K on extraction performance from swabs, five of the skin swab samples stored in ATL buffer (AN26 to AN30) were treated with 20 µl Proteinase K during lysis, the remaining five (AN31 to AN35) were lysed without Proteinase K. Fin clip samples were always treated with Proteinase K during lysis.

DNA yield was quantified with a Quantus^™^ Fluorometer using the QuantiFluor^®^ dsDNA System. DNA integrity and purity was assessed using A260/A280 and A260/230 ratios from NanoDrop^®^ ND-1000 Spectrophotometer measurements. Ratio cut-offs were set to ensure purity from RNA, protein, or other organic contaminants. Samples passed internal (our) quality control (QC), with a minimum DNA concentration of 20 ng/μl (some after evaporating excessive liquid to increase concentration), an A260/A280 absorbance ratio above 1.3, and an A260/A230 absorbance ratio above 1.8. A total of 1.2 μg DNA per sample from all samples passing internal QC (iQC) was used for WGS with Novogene GmbH. Novogene GmbH performed further DNA quality assessment (fragment size distribution) on a fragment analyser (Agilent 5400, 3 μl). Samples that were classified as severely degraded by Novogene GmbH were deemed to have failed external QC (eQC). However, samples failing eQC were revised internally and manually by inspecting the fragment length distribution. If fragment peaks were overwhelmingly above 3000 bp, samples were still subjected to library preparation and sequencing.

### Sequencing, read quality filtering, and mapping

WGS was performed using PCR-free library preparation. Libraries were sequenced paired-end 150 bp on an Illumina NovaSeq X Plus to a target coverage of 15. Raw reads were quality checked using Fastqc v.0.12.1 [12], and quality trimmed with AdapterRemoval v.2.3.3 [13]. The trimmed and cleaned reads were mapped to the *P. phoxinus* reference genome (NCBI accession number: PRJNA1030284) [14] using BWA v.2.2.1 [15]. Mapped reads were sorted with SAMtools v.1.19.2 [16] and optical duplicates removed using picard v.3.2.0 [17]. Mapping statistics were calculated using Qualimap v.2.3 [18]. Full parameter settings for all analyses can be found in the attached scripts in the *Availability of data and materials* section.

### Statistics and assessment of sequencing performance

The statistical significance of differences in DNA quality post extraction was compared between fin clips, gill swabs, skin swabs, and skin swabs pre-treated with ATL buffer with or without Proteinase K. DNA quality was assessed using DNA concentration, 260/280 ratio, and 260/230 ratio per sampling group. To ensure that DNA quality was normally distributed, Shapiro–Wilk tests were performed for each category. If data was normally distributed (p > 0.05), a one-way ANOVA was used to assess significant differences between sampling groups. If data was not normally distributed (p < 0.05), a Kruskal–Wallis test was performed and if significant, a post-hoc Dunn’s multiple comparisons test (Bonferroni-Sidak adjustment) was used to compare tissue sources individually.

Sequencing performance for swab and fin clip DNA was compared by calculating the percentage of reads retained after quality filtering and the percentage of reads mapped to the reference genome. The percentage of retained reads was calculated by dividing the number of retained reads after filtering by the number of raw reads multiplied by 100. The percentage of mapped reads was calculated by dividing the number of mapped reads by the number of retained reads multiplied by 100. Mean sequencing coverage and read length is reported for swabs and fin clip WGS data. Sequencing performance was statistically compared between tissue sources as described above for DNA quality.

## Results

### Fin clips outperform swabs in DNA concentration

**Fig. 1:**
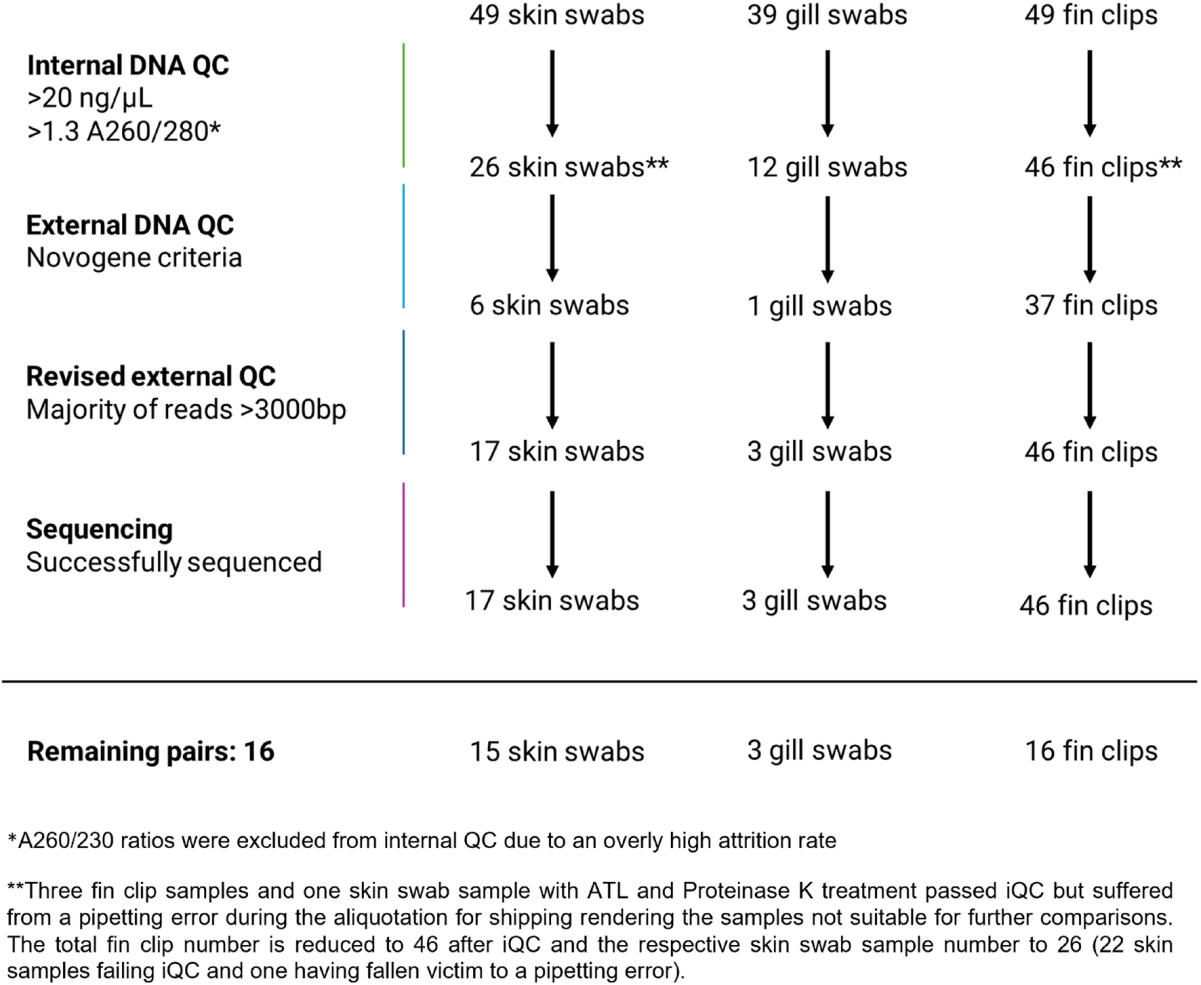
DNA isolation workflow from swabs and fin clips. 85 swabs (46 skin, 39 gill swabs) and 46 fin clips were taken from 46 specimens for paired WGS. The workflow is separated into samples that failed internal QC (DNA purity and concentration), and external QC (performed by Novogene GmbH; DNA fragmentation). Remaining sample pairs are those where at least one swab (skin, gill, or both) and a fin clip from the same specimen passed all stages. In the end 16 pairs consisting of 16 fin clips and 15 skin and 3 gill swabs could be compared

Fin clips yielded a considerably higher DNA quantity than swabs, with no instances of fin clips failing iQC, in contrast to 49 swabs in total that were unable to meet the required DNA concentration of 20 ng/μl (Fig. 1**, **Table 1). Skin swabs treated with Proteinase K (swabs AN26 to AN30, n = 5) presented with a mean DNA yield of 73.60 ± 22.63 ng/μl. This was a higher yield compared to skin swabs that were not treated with Proteinase K but stored in ATL buffer after collection (35.87 ± 36.68 ng/μl, n = 5). Both still had a higher mean DNA yield compared to swab samples not stored in ATL buffer and not treated with Proteinase K (24.61 ± 23.91 ng/μl, n = 39). The comparison between swabs with and without Proteinase K and ATL buffer pre-treatment is however limited in its statistical reliability due to low sample sizes. Though no samples failed the A260/280 ratio cut-off of 1.3, 84 swabs and 14 fin clips failed the A260/230 cut-off of 1.8. This was possibly due to issues concerning unusual flocculation in the AL buffer, which is used to further denature proteins and macromolecules, applied to some of the fin clip and swab lysates. Since flocculate could visually be separated from lysate, extractions failing the A260/280 cut-off but fulfilling all other criteria still passed iQC. eQC did not report any impurities in these samples. Nine fin clip and 31 swab samples failed eQC due to containing degraded DNA (see supplementary file 2). A revision process of samples failing eQC deemed eleven skin swab, two gill swab, and nine fin clip samples fit for sequencing despite failing eQC. Overall, 65.31% of swabs and only 6.12% of fin clips failed iQC, eQC, or revised eQC and were therefore not sequenced (Table 1). Notably, gill swabs performed worse than skin swabs with 92.31% QC failure compared to 69.39%, respectively. In the end, 17 skin swabs, 3 gill swabs, and 46 fin clips were sequenced. The resulting 16 swab-fin clip pairs were used for WGS comparison.

**Table 1:** Breakdown of DNA sample performance according to internal QC (iQC)

	Skin swabs			Gill swabs	Fin clips
	Untreated	ATL buffer	ATL buffer + Prot K		
**Number of Samples**	**39**	**5**	**5**	**39**	**49**
Final DNA concentration (ng/μL)	24.61 ± 23.91*	35.87 ± 36.68	73.60 ± 22.63*	16.27 ± 16.78*	71.21 ± 34.71*
Samples failing DNA concentration cut-off (< 20 ng/μL)	19	3	0	27	0
260/280 absorbance ratio	1.76 ± 0.19*	1.57 ± 0.17*	1.83 ± 0.05	1.80 ± 0.19*	1.86 ± 0.12*
260/230 absorbance ratio	0.71 ± 0.41*	1.26 ± 0.52	1.78 ± 0.22*	0.75 ± 0.39*	1.92 ± 0.62*
Samples failing 260/280 absorbance ratio (< 1.3)	0	0	0	0	0
Samples failing 260/230 absorbance ratio (< 1.8)	39	4	2	39	14
**Samples passing iQC for sequencing**	**20 (51.28%)**	**2 (40%)**	5 (100%)**	**12 (30.77%)**	49 (100%)**
Samples failing eQC	20	0	0	11	12
**Samples passing after revision of eQC for sequencing**^+^	**11 (55%)**	**2 (40%)**	4 (80%)**	**3 (7.69%)**	46 (94%)**
**Samples successfully sequenced**	**11 (55%)**	**2 (40%)**	4 (80%)**	**3 (7.69%)**	46 (94%)**

Means of basic DNA quality and quantity assessments were calculated after DNA isolation. Significant differences between means were assessed with Dunn’s multiple comparisons test (see supplementary file 3) after significant Kruskal–Wallis test results. Samples sufficient in quantity and quality (passing iQC) were sent for external QC (eQC) and WGS

Bold rows are meant to represent important/more relevant rows, i.e., number of samplesm samples passing iQC, samples passinf after revision of eQC, samples successfully sequenced

^*^p < 0.05

^**^ Three fin clip samples and one skin swab sample with ATL and Proteinase K treatment passed iQC but suffered from a pipetting error during the aliquotation for shipping rendering the samples not suitable for further comparisons. The total fin clip number is reduced to 46 after iQC and the respective skin swab sample number to four. Consequently, all (100%) tissue and the respective skin swab samples that did not suffer from errors during the pipetting process passed eQC and were successfully sequenced

^ +^ Although most samples did not pass eQC, they were still submitted to library preparation as Novogene QC standards are adapted for clinical samples. DNA extractions from non-clinical samples are more likely to not pass those QC standards

### Swabs match fin clips in WGS performance

Bearing in mind the small sample size for successfully sequenced gill swabs, there was only a significant difference in the percentage of retained reads after quality filtering (H(2) = 6.284, p = 0.0432, Table 2) between skin swabs and fin clips (p = 0.0389, Z = 2.484, Table 3). However, skin swabs still performed well since the percentage of retained skin swab reads (99.98 ± 0.02%) was only marginally smaller than that of fin clips (99.99 ± 0.002%, Table 2). No significant difference was found between all tissues for the percentages of reads mapping to the *P. phoxinus* reference genome (H(2) = 3.680, p = 0.159).

**Table 2:** Sequencing performance of fin clip, gill swab, and skin swab DNA extractions

	Fin clips	Skin swabs	Gill swabs
Number of samples	16	15	3
Total raw reads per sample	95,385,777 ± 3E + 7	100,620,434 ± 2E + 7	117,317,537 ± 1E + 7
Retained reads after quality filtering	95,375,602 ± 3E + 7	100,598,146 ± 2E + 7	117,304,915 ± 1E + 7
% of retained reads after filtering	99.99 ± 0.002%*	99.98 ± 0.02%*	99.99 ± 0.002%
Reads mapping to reference genome	70,226,556	71,256,195	85,762,531
% of mapped reads	73.75 ± 0.007%	70.47 ± 0.05%	73 ± 0.008%
Coverage	9.8 ± 2.8-fold	9.9 ± 1.8-fold	11.2 ± 1.2-fold
Read length	167.9 ± 3.5 bp	168.6 ± 7.3 bp	163.8 ± 2.0 bp

Means and standard deviation of seven different Qualimap summary statistics from the 16 fin clip-swab pairs: summaries from 16 fin clip, 3 gill swab, and 15 skin swab samples are presented. A significant p-value from Dunn’s multiple comparisons test (see Table 3) comparing the percentage of retained reads after quality filtering of skin swabs, gill swabs and fin clips is indicated with an asterisk. Only the comparison between skin swab and fin clip was significant

^*^p < 0.05

**Table 3:** Dunn’s multiple comparisons test results for WGS data

% of retained reads			
	Z	P.unadj	P.adj
Gill-fin	0.365774	0.714534	1.0000000
Skin-fin	2.484420	0.012976	0.0389288
Skin-gill	1.047927	0.294672	0.8840166

Differences between skin swabs, gill swabs, and fin clips in percentage of retained reads after quality filtering were investigated with Dunn’s multiple comparisons test after significant Kruskal–Wallis test results

## Discussion

### Swabs as a viable alternative to fin clips with improved extraction protocol

Fin clips generally outperformed skin and gill swabs in DNA yield, which matches results from Breacker et al. [9]. Although manufacturer instructions specified that swabs did not require storage in buffer after swabbing, DNA concentrations obtained from skin swabs treated with Proteinase K and stored in ATL buffer after collection were notably higher compared to those without standard treatment. These pre-treated samples met the concentration cut-off required for sequencing in 100% of cases. The positive effect of Proteinase K treatment on DNA concentration matches reports by Tsuji et al. [19] with a similar problem of low DNA yields from environmental DNA water samples. Since Eurasian minnows do not produce a lot of mucus after death and DNA water samples often contain intracellular DNA, including from fish skin mucus, results by Tsuji et al. [19] are comparable to the swabs taken here. An improved extraction protocol employing direct storage of swabs in lysis buffer and including Proteinase K during lysis could therefore make skin swabs a viable alternative to fin clips for WGS evidenced by the comparable performance of swabs and fin clips in WGS data mapping to the *P. phoxinus* reference genome. The only major issue for both swab and fin clip lysates was the high rate of failure for the A260/230 ratio, potentially due to AL buffer flocculation. An additional measure to avoid flocculation could be routinely heating the AL buffer to 56 °C before usage [20].

Tilley et al. [10] demonstrated skin swabs were less invasive than fin clips by causing fewer changes in behaviour and physiology but this is unlikely to be the case for gill swabs. Here, the swabs used for sample collection were too large to fit more than the tip under the operculum of very small fish (< 5 cm). The large size of the tips caused a lot of bleeding and tissue damage within the gills, rendering them highly invasive [21]. Additionally, since only the swab tip came in contact with the specimen, little genetic material could be extracted from gill swabs. This may explain why gill swabs performed worse than skin swabs in QCs. Smaller swabs would probably have allowed for a higher yield of DNA from the gills of small fish without invasive side effects. Testing the performance of smaller swabs for WGS data quality and quantity may reveal that gill swabs could be just as suitable for WGS as skin swabs have proven [11]. Generally, gill swabs are harder to retrieve and we generally advise against their collection, especially considering DNA from skin swabs was of equal quality contrary to initial expectation.

Overall, skin swabs may be used as a less-invasive alternative to conventional fin clipping for WGS in Eurasian minnows and fishes of similar size and similar skin composition. Recommended improvements to the DNA extraction process should be followed to match the performance of fin clips in WGS.

## Limitations

This study was performed on a single fish species from freshwater. Extractions from skin swabs from species with a thicker or less thick mucus layer might perform differently during sequencing. Only one DNA extraction protocol was employed and only one swab size and type were tested. Samples collected with different swabs and extracted with different extraction protocols may yield different DNA extraction results and sequencing performances. Sample sizes were limited for the comparison between samples pre-treated with Proteinase K and ATL (5 and 5) buffer and are therefore statistically unreliable. We also did not test whether freezing of swabs stored in ATL would have an effect on sequencing performance.

## Supplementary Information

Supplementary material 1. Summary of individual sample notes throughout DNA extraction and WGS sequencing process.

Supplementary material 2. DNA integrity test results for gill and skin swab, and fin clip DNA samples. DNA molecule lengthis described on the x-axis in base pairsand measured quantitatively in relative fluorescence unitsin supplementary figures 1 to 29. Electrophoresis results are presented for each sample on the right with standard DNA fragment sizes indicated.If a sample failed external QCsuch was indicated in the legend.

Supplementary material 3: Table 1. Dunn’s multiple comparisons test results for DNA quality data post DNA extraction. Differences in 260/280 ratio, 260/230 ratio, and final DNA concentration were investigated between fin clips, gill swabs, skin swabs, and skin swabs pre-treated with ATL buffer and/or Proteinase K. A Dunn’s multiple comparisons test was employed for the comparison after significant Kruskal-Wallis test results.

## Data Availability

All TOO scripts used for bioinformatic analysis are available at https://doi.org/10.5281/zenodo.13838368. The datasets generated and analysed during the current study are available in the NCBI GenBank repository, under BioProject ID PRJNA1166076.

## Abbreviations

WGS: Whole genome sequencing
QC: Quality control
iQC: Internal quality control
eQC: External quality control
ATL: Tissue lysis buffer, Qiagen®
