# Supplementary material for: Comparison of whole genome sequencing performance from fish swabs and fin clips
Source: BMC Res Notes. 2025 Jan 15;18:15. doi: 10.1186/s13104-024-07075-1 (PMC11734550; doi:10.1186/s13104-024-07075-1)
Supplement: Supplementary file 2 — Supplementary material 2. DNA integrity test results for gill and skin swab, and fin clip DNA samples. DNA molecule lengthis described on the x-axis in base pairsand measured quantitatively in relative fluorescence unitsin supplementary figures 1 to 29. Electrophoresis results are presented for each sample on the right with standard DNA fragment sizes indicated.If a sample failed external QCsuch was indicated in the legend. [file 13104_2024_7075_MOESM2_ESM.docx]

**Supplementary file 2. DNA integrity test results for gill and skin swabs, and fin clip DNA samples.** DNA molecule length (measured on an Agilent 5400 fragment analyser is described on the x-axis in base pairs (bp) and measured quantitatively in relative fluorescence units (RFU). Gel electrophoresis results are presented for each sample on the right with standard DNA fragment sizes indicated. If a sample failed external QC (eQC) such was indicated in the legend.


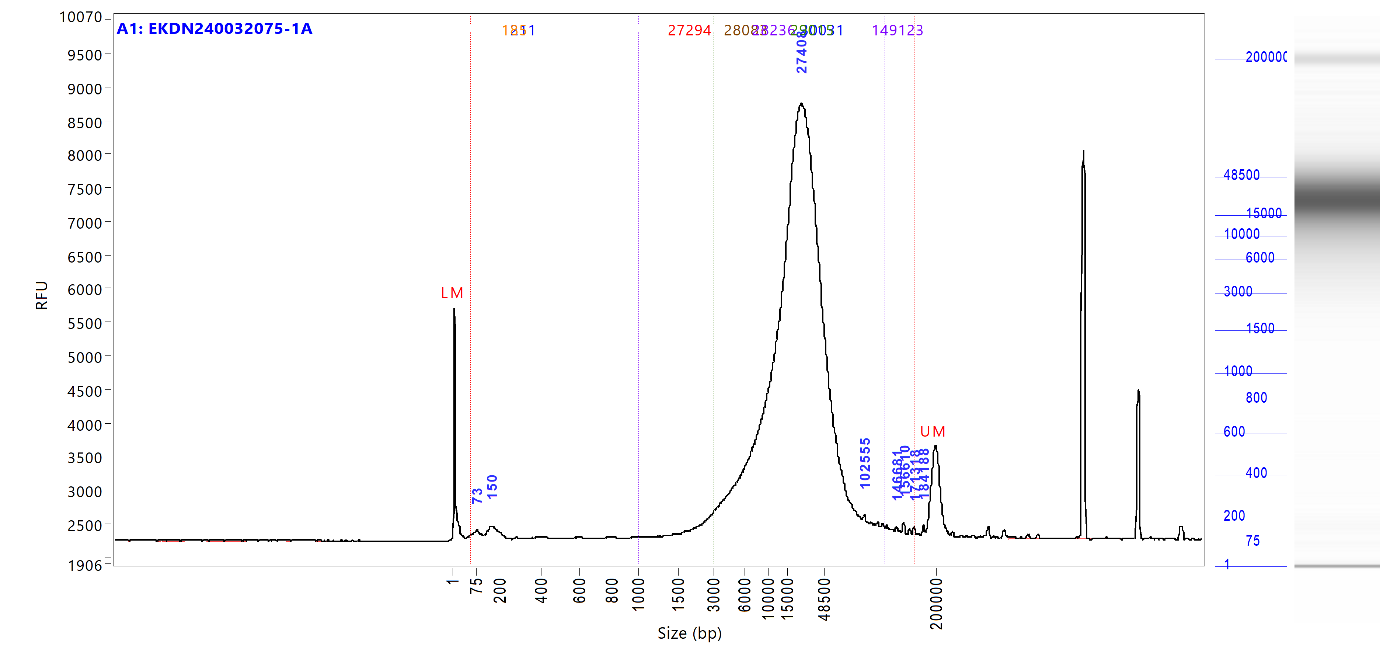

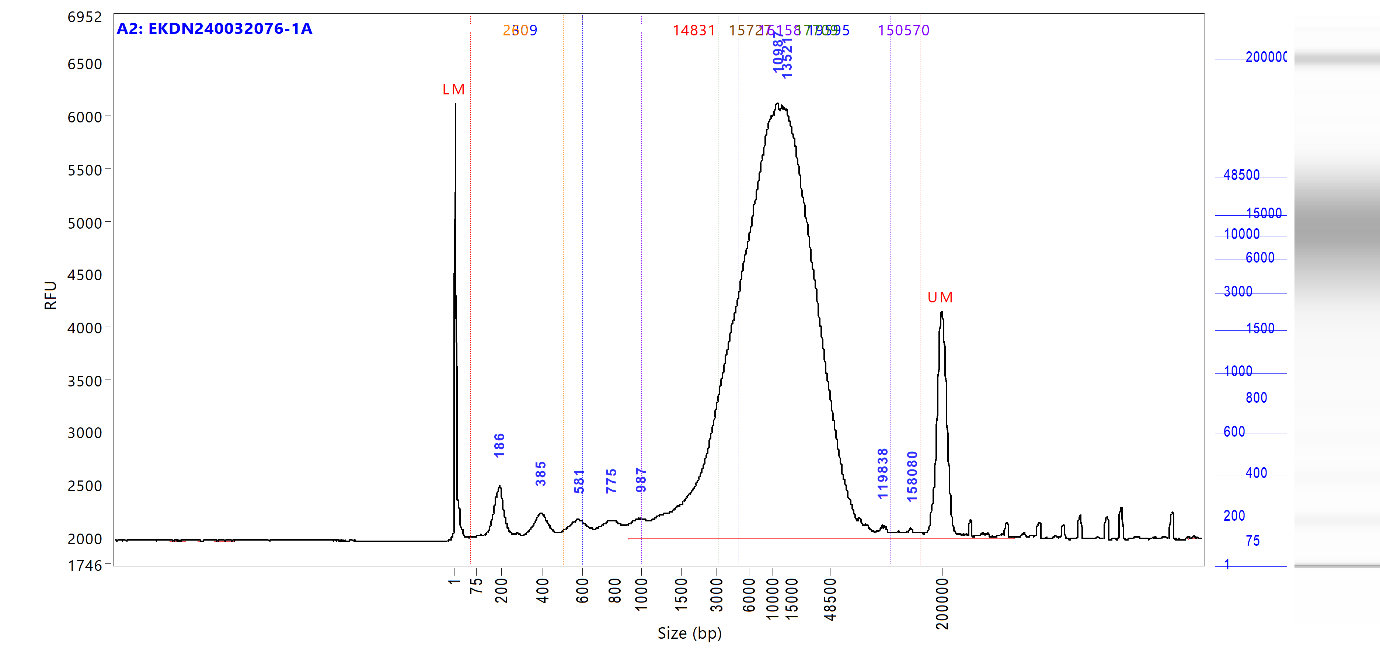


**Supplementary Figure 1. Integrity test results for samples AN35_fin (top) and AN34_fin (bottom).**


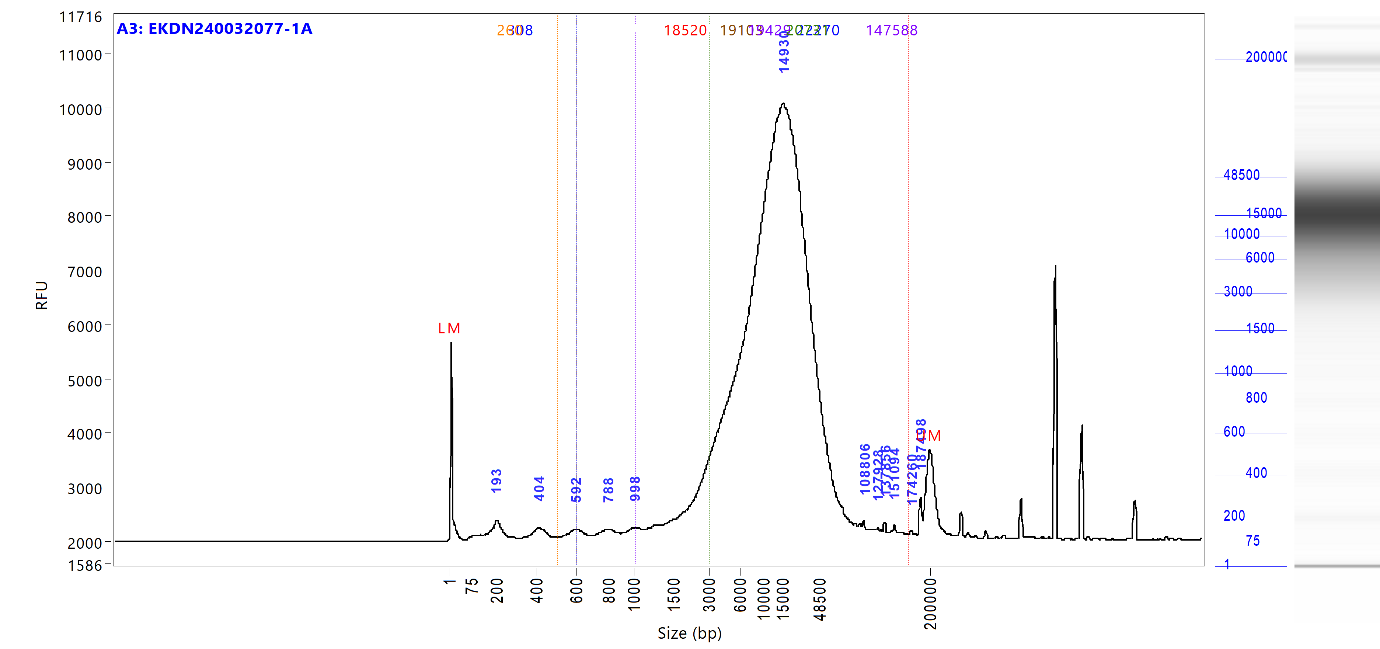

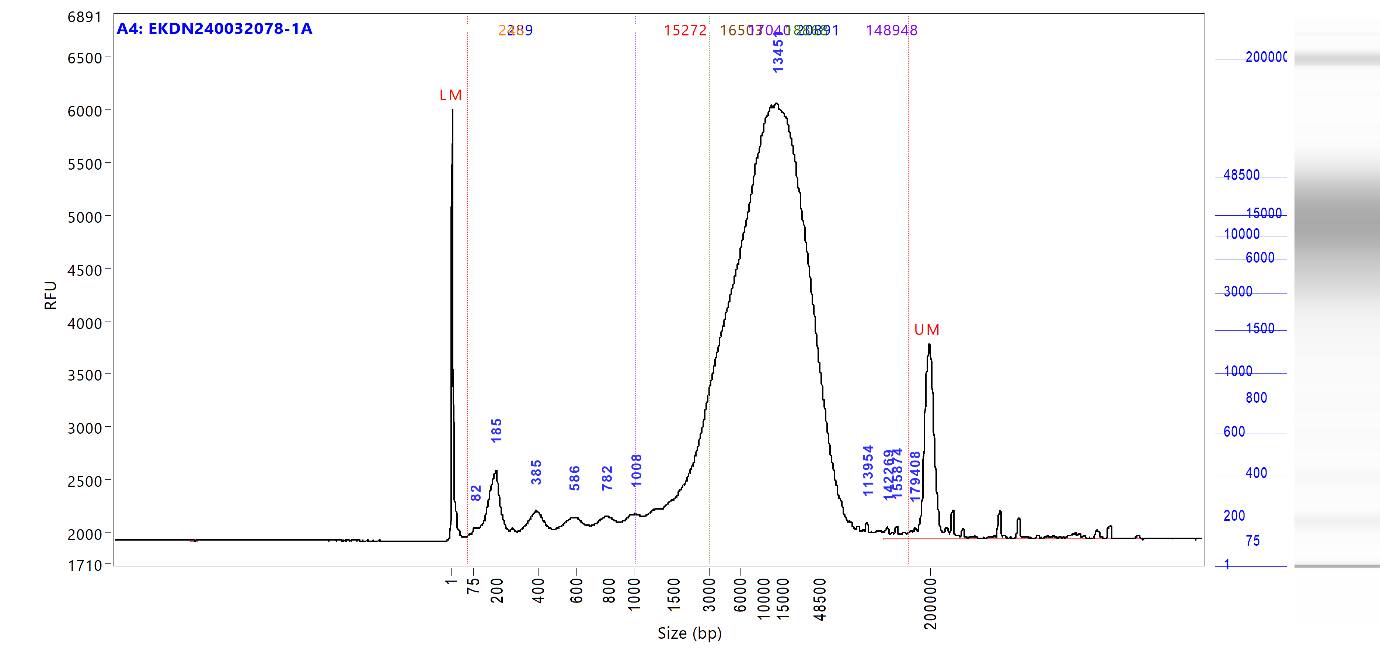

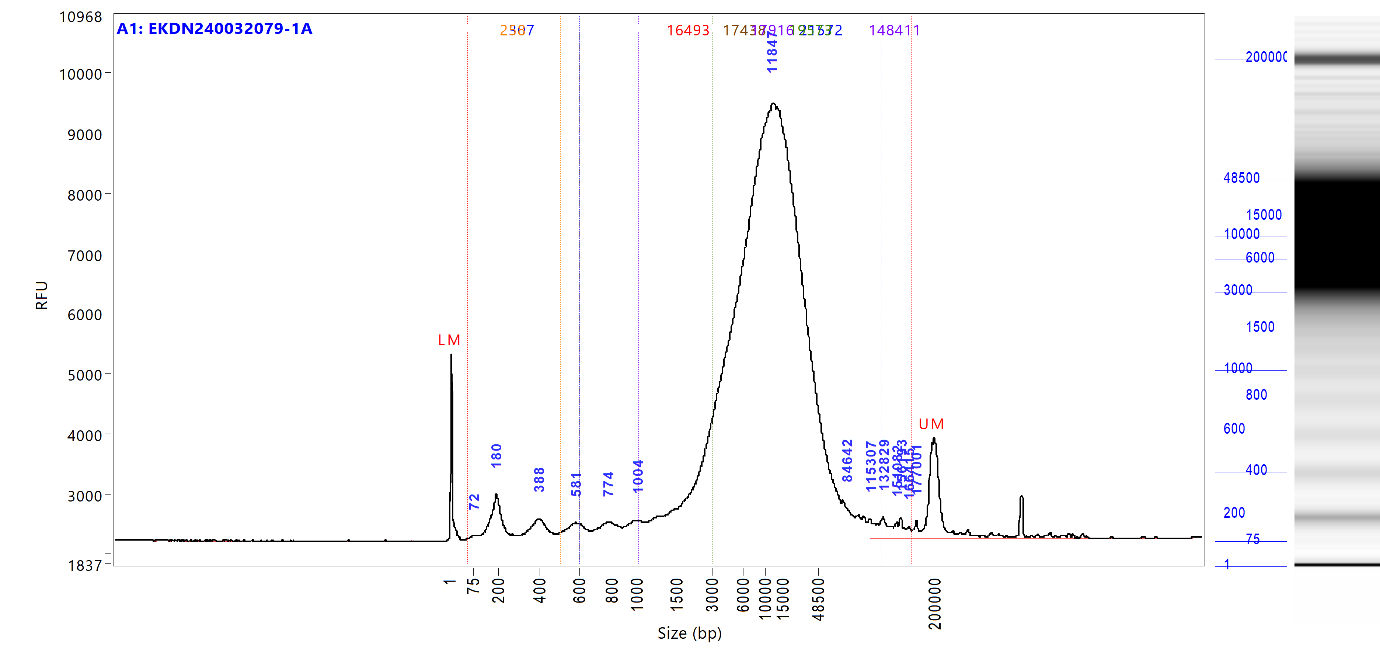


**Supplementary Figure 2. Integrity test results for samples AN33_fin (top), AN32_fin (middle) and AN31_fin (bottom).**


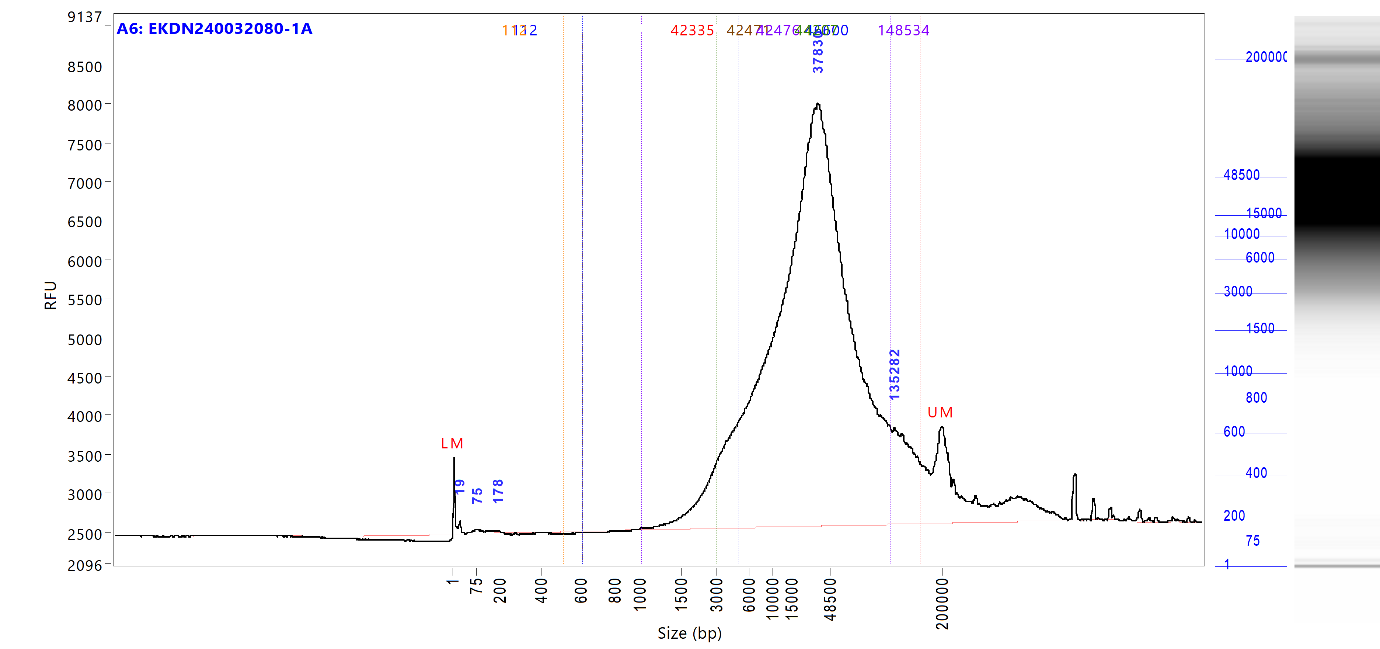

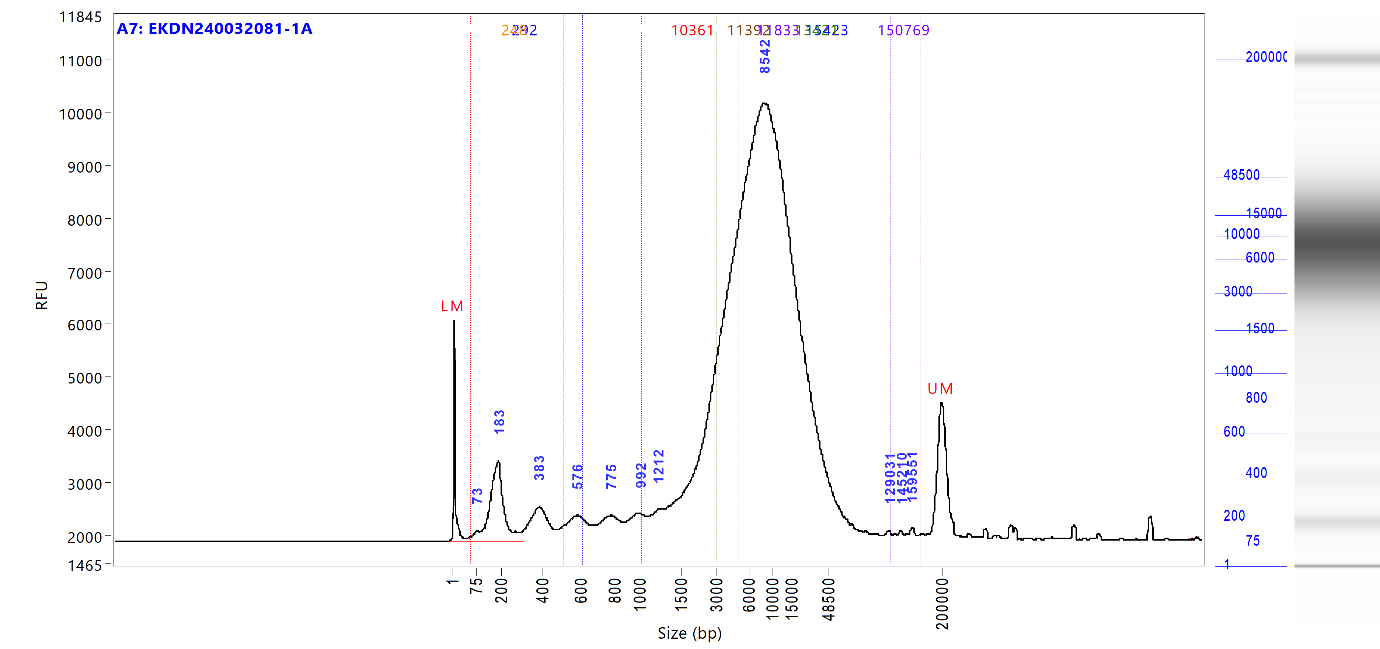

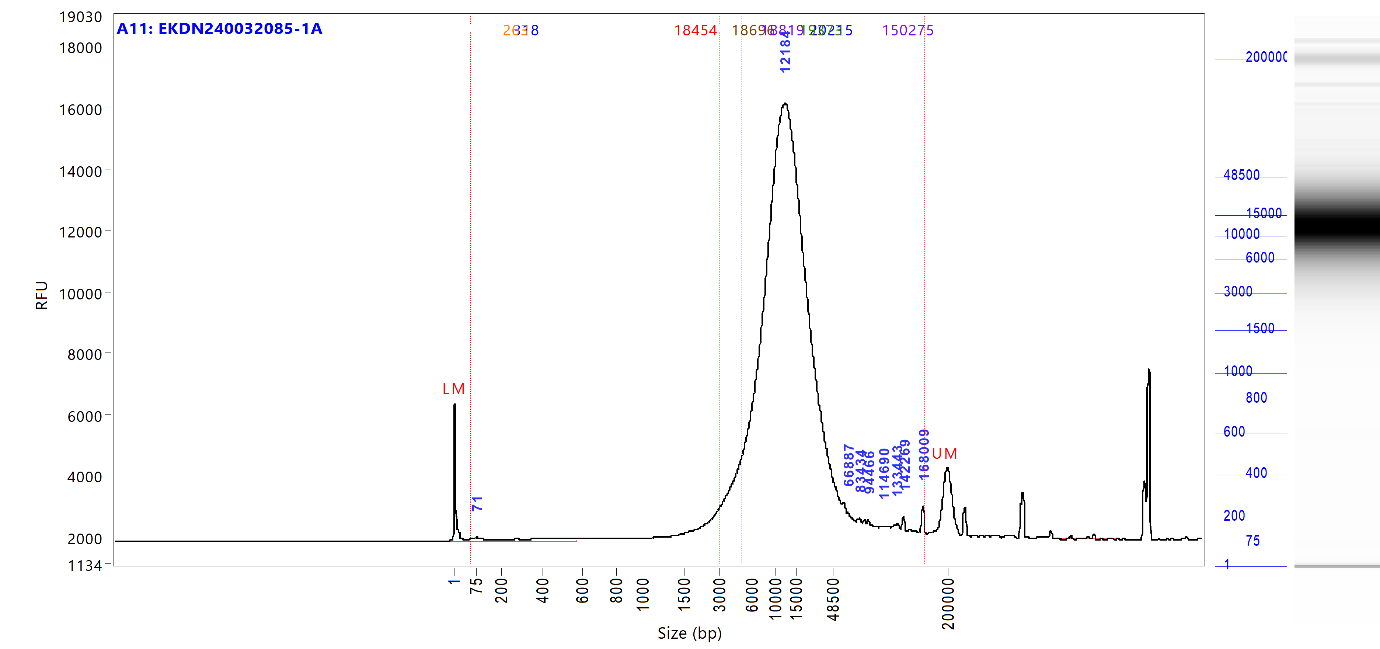


**Supplementary Figure 3. Integrity test results for samples AN30_fin (top), AN29_fin (middle) and AN33_skin (bottom).**


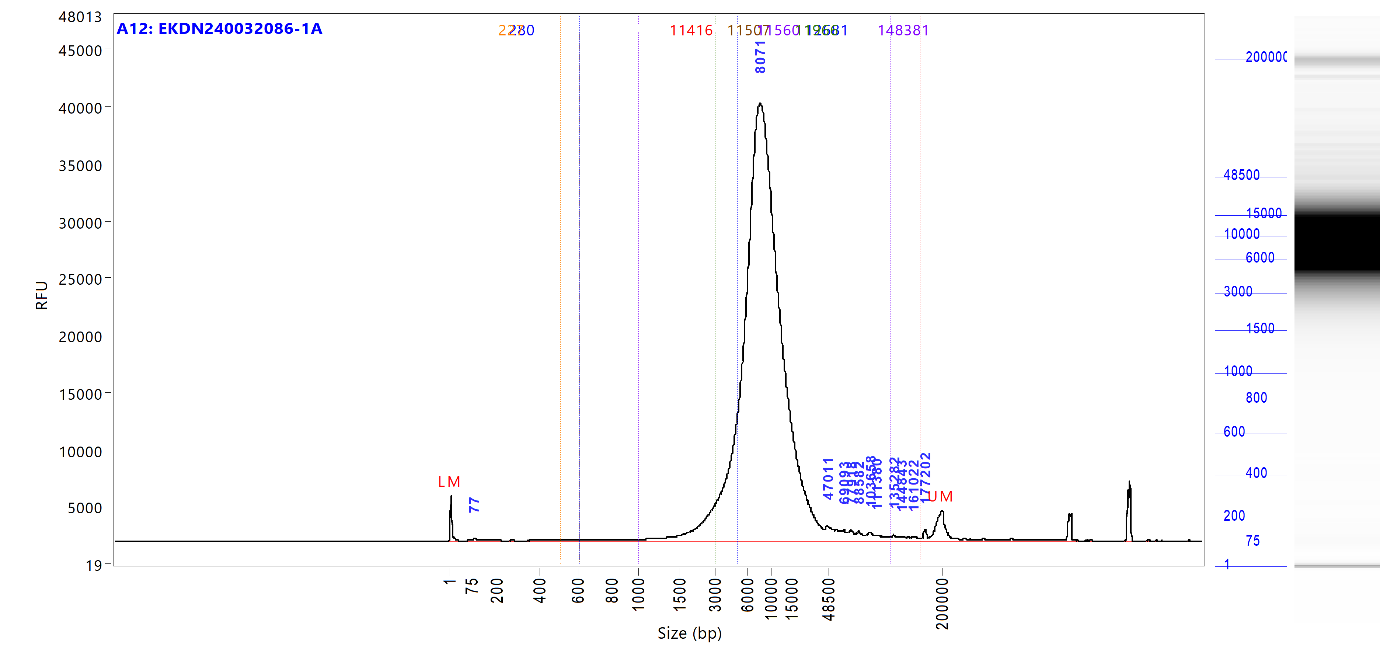

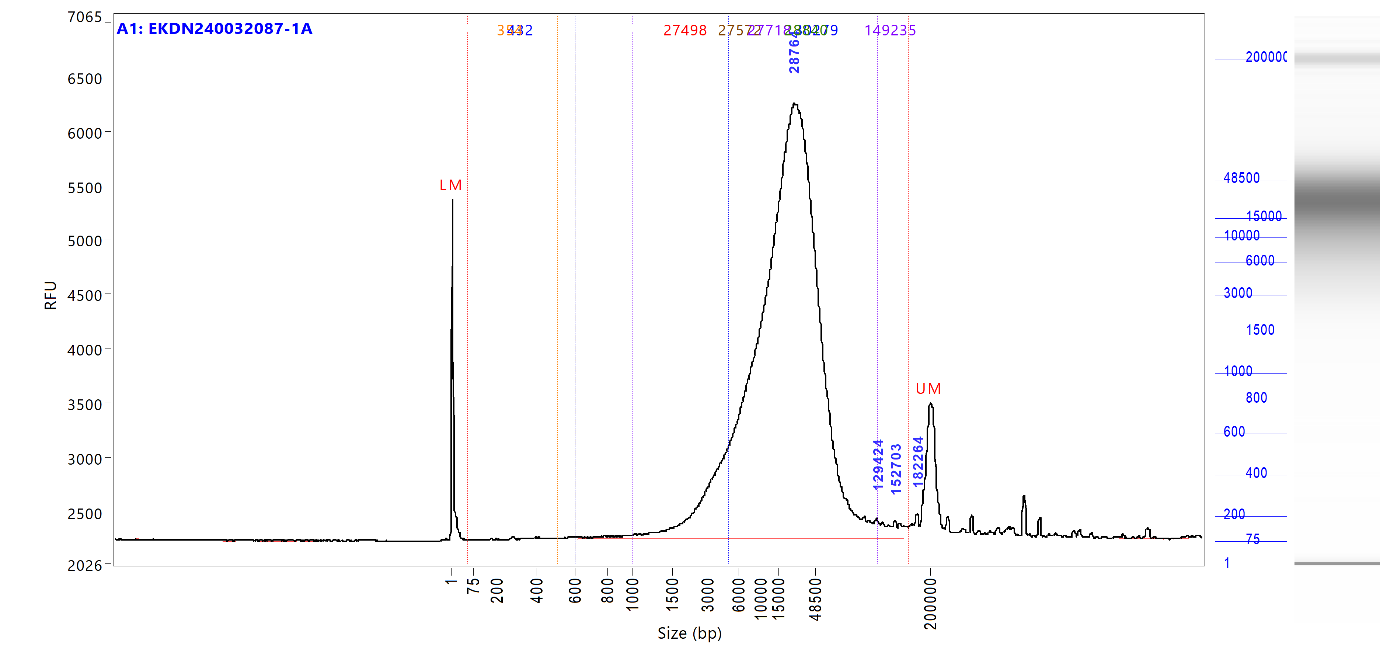

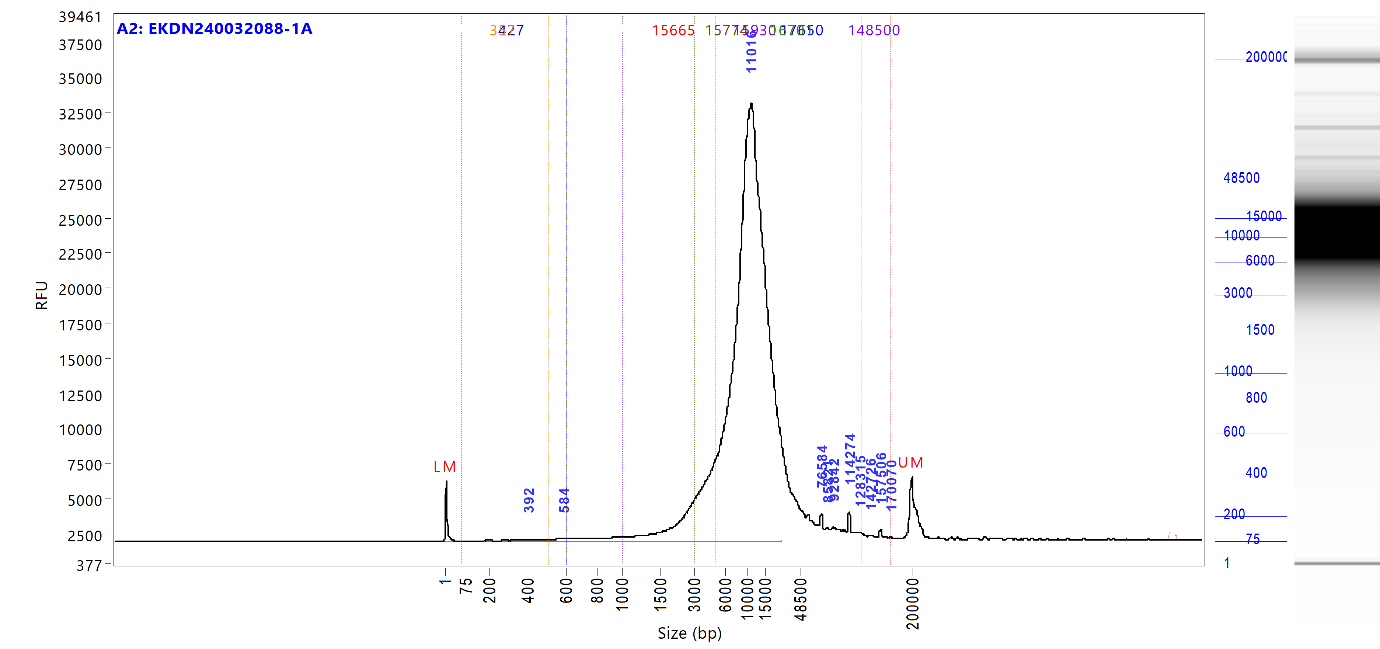


**Supplementary Figure 4. Integrity test results for samples AN32_skin (top), AN30_skin (middle) and AN29_skin (bottom).**


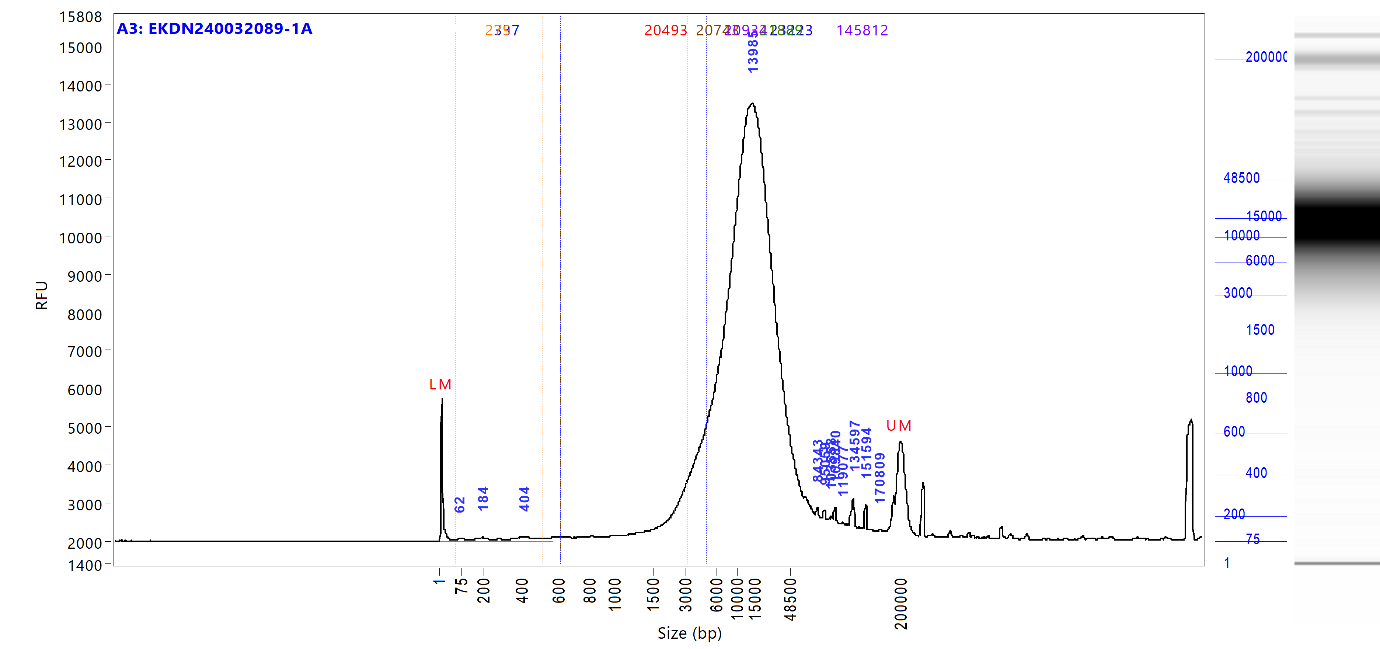

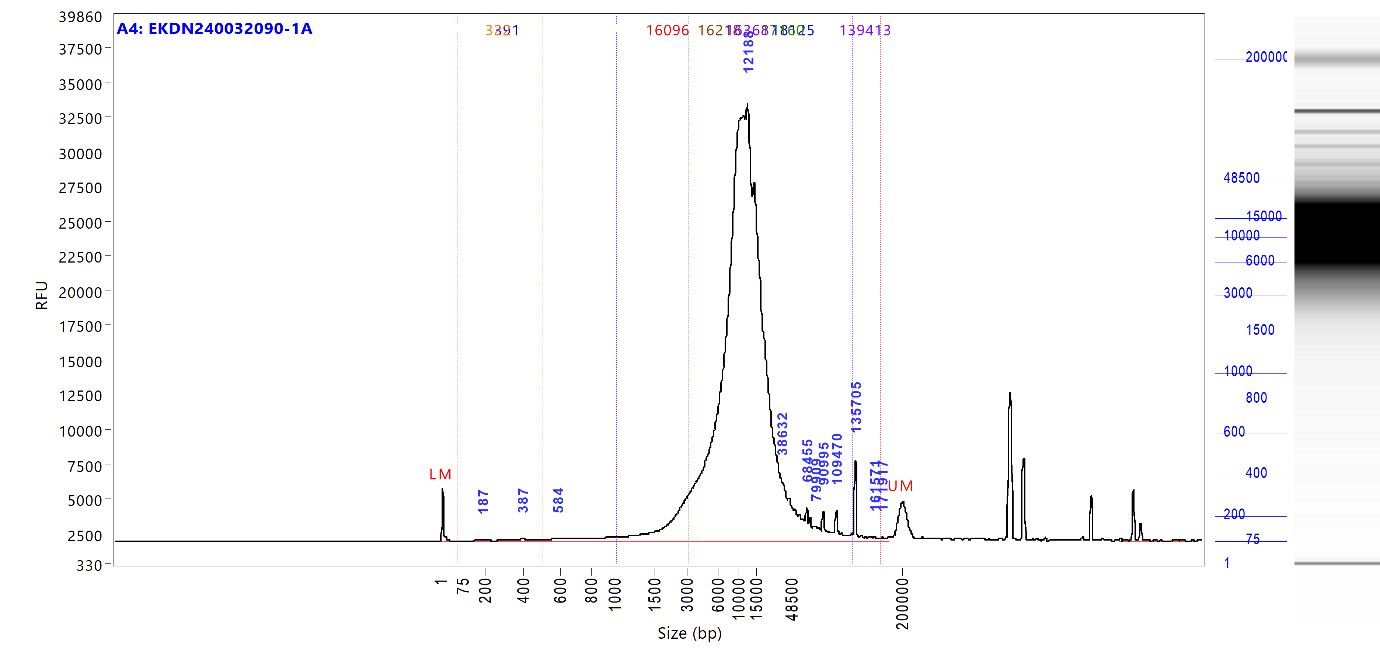

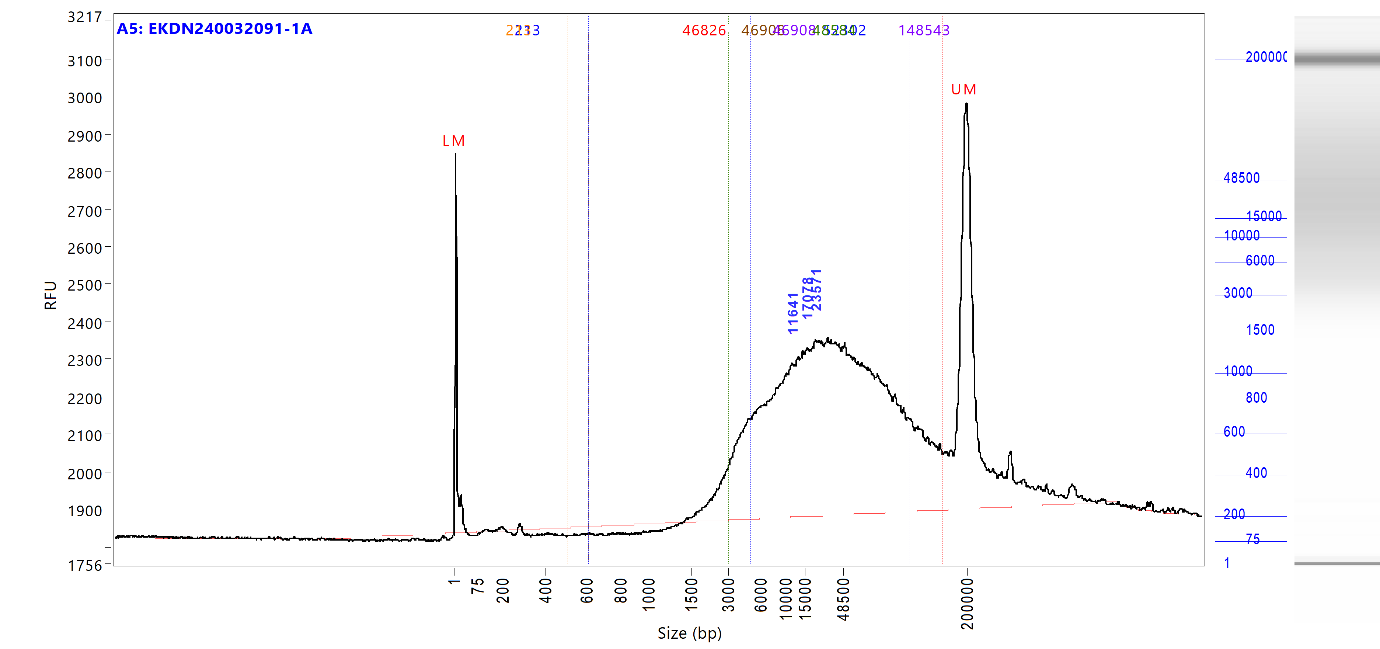


**Supplementary Figure 5. Integrity test results for samples AN27_skin (top), AN26_skin (middle) and AN25_fin (bottom, failed eQC).**


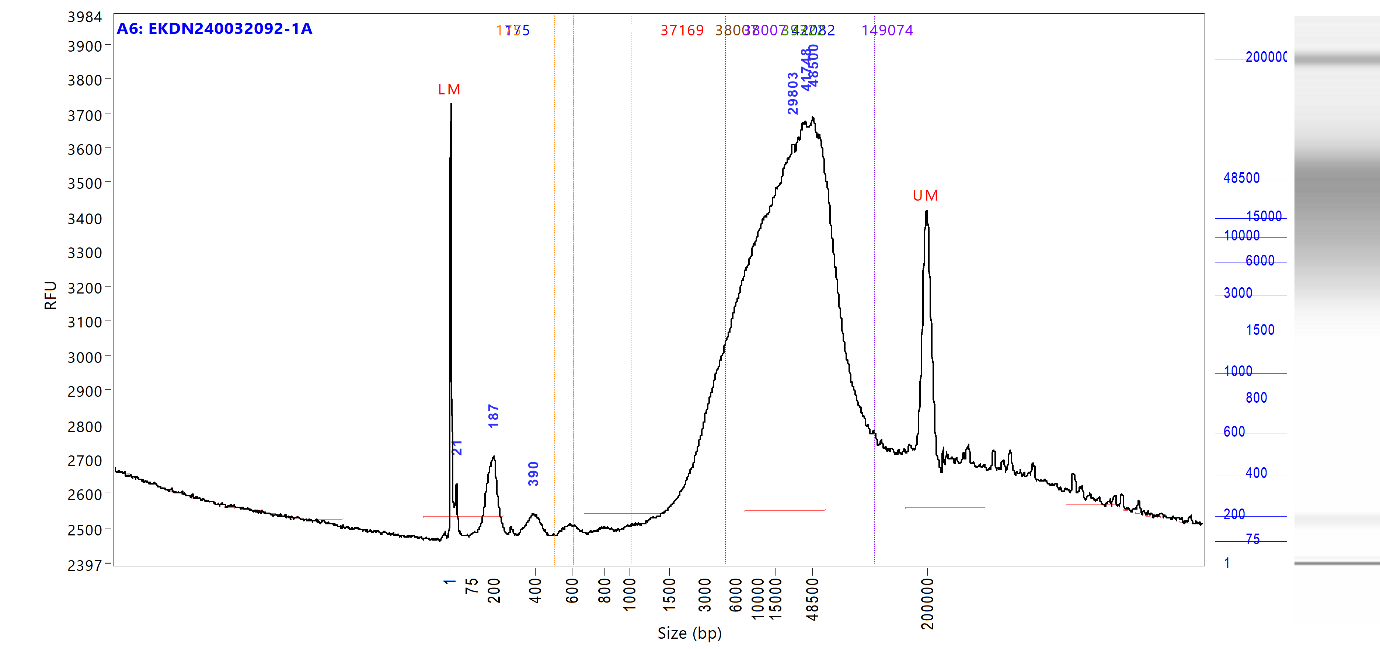

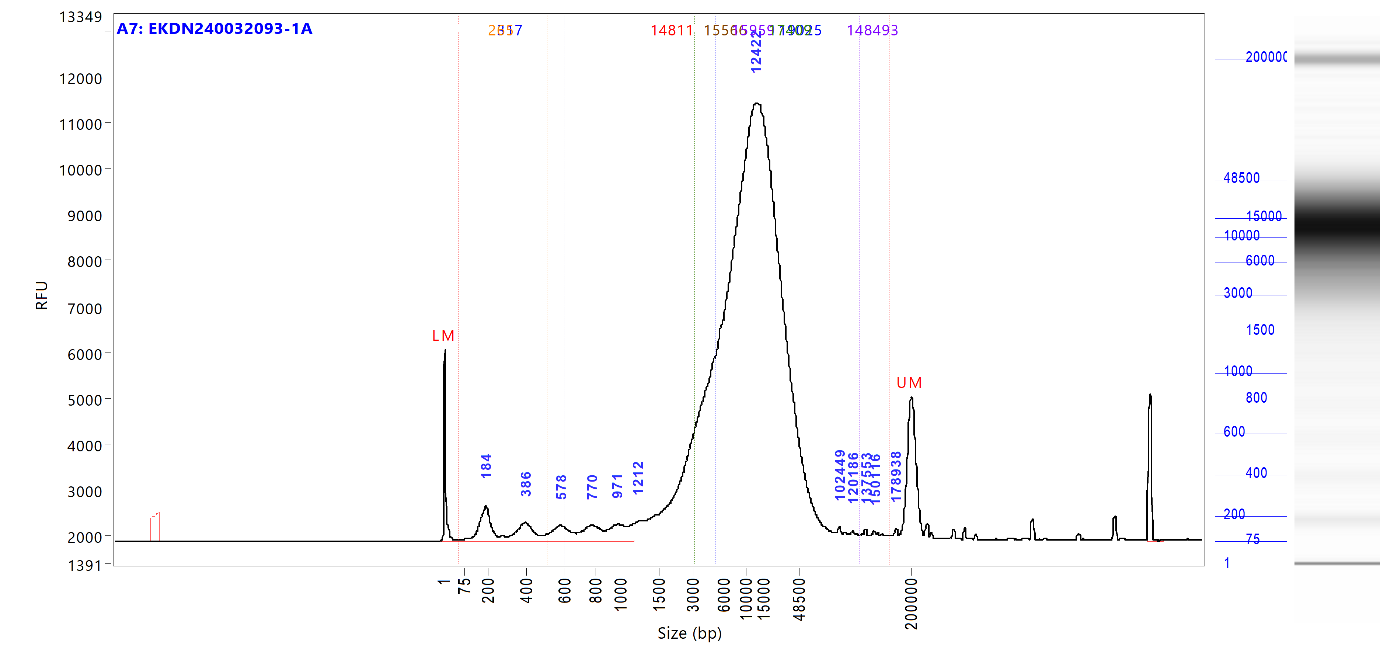

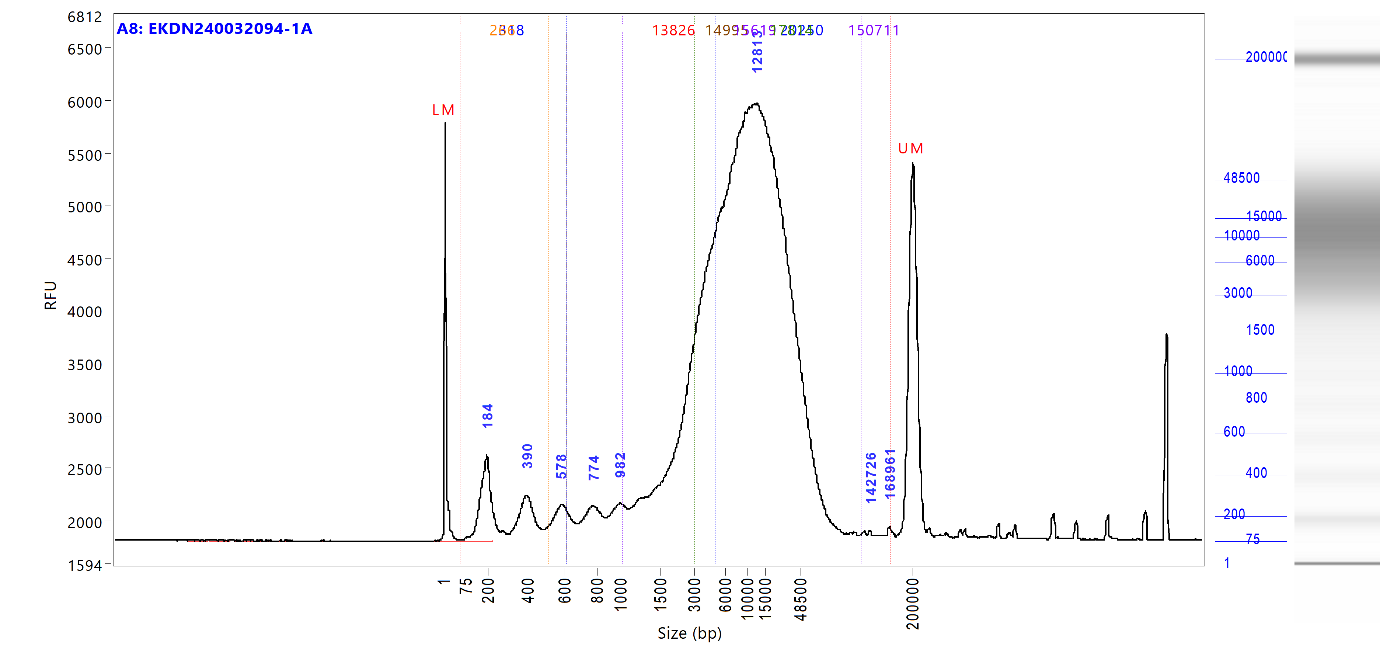


**Supplementary Figure 6. Integrity test results for samples AN24_fin (top), AN23_fin (middle) and AN22_fin (bottom).**


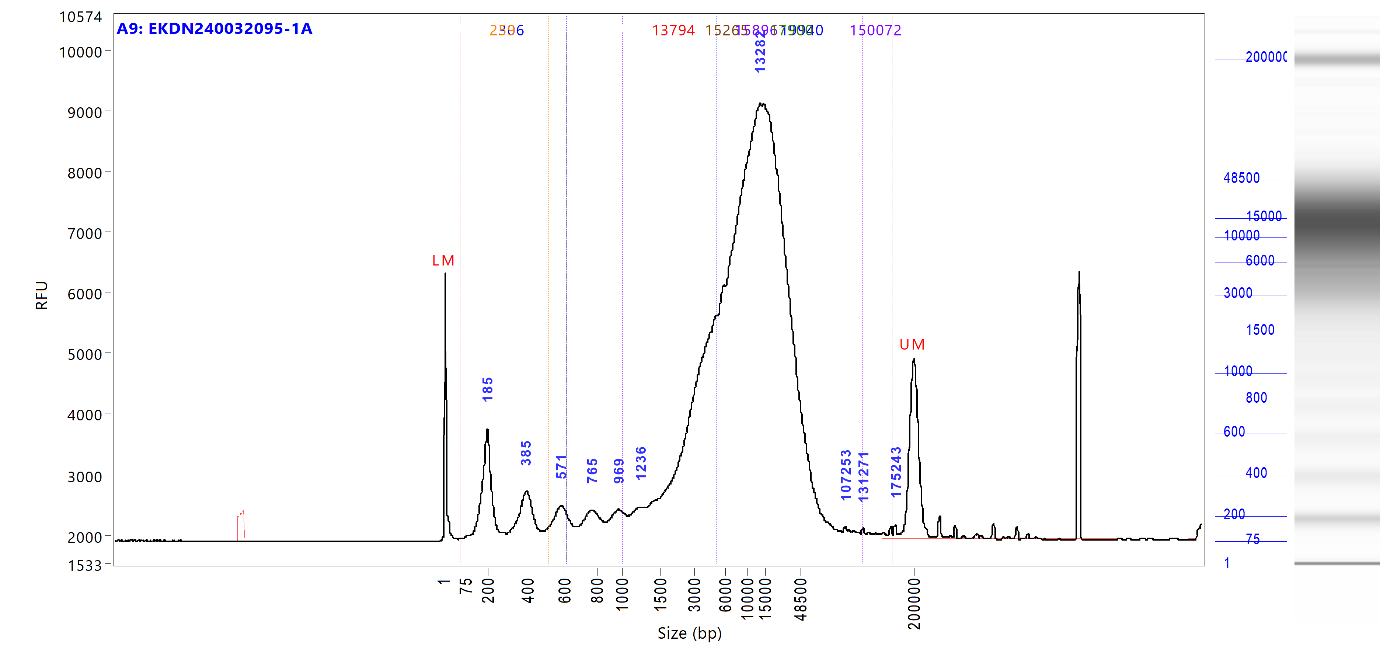

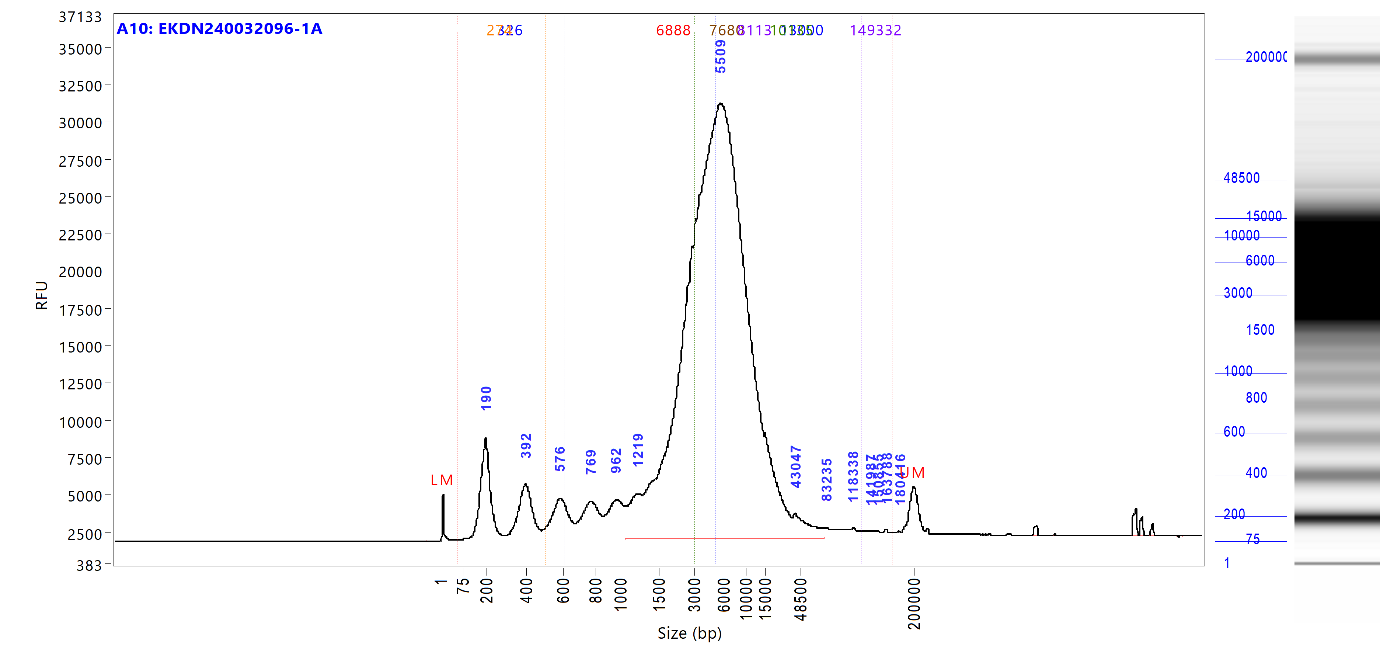

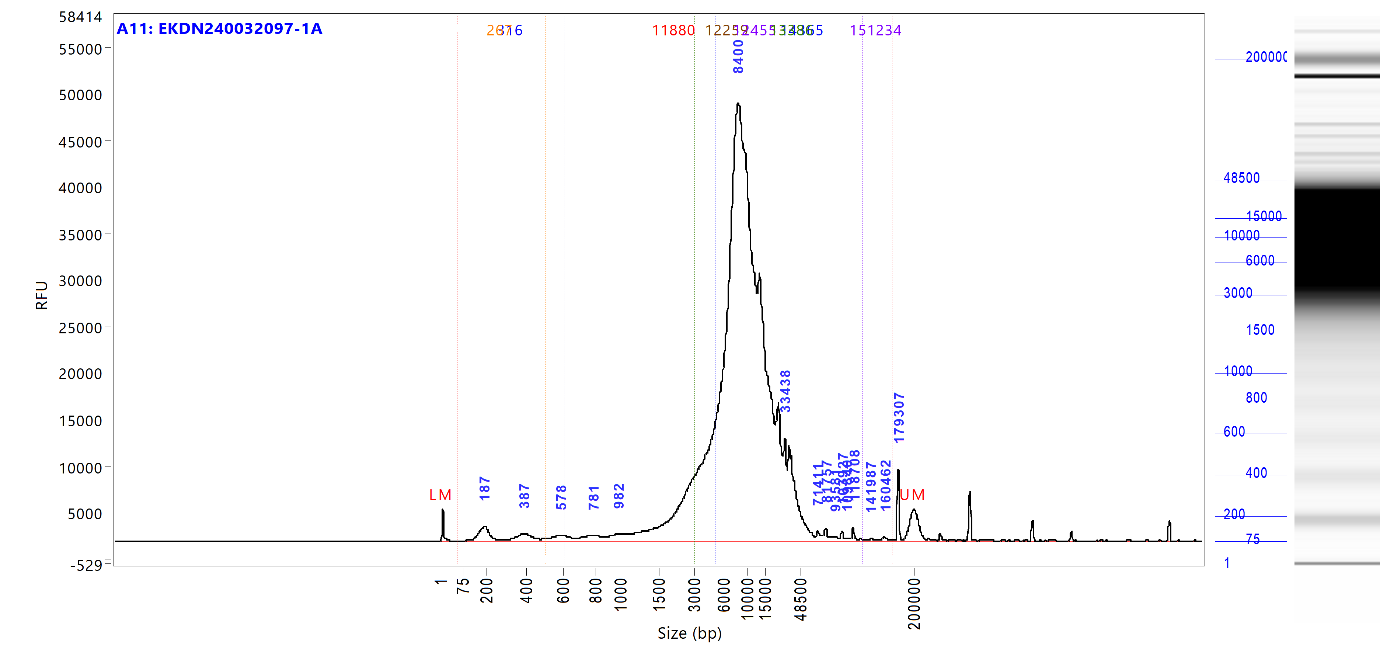


**Supplementary Figure 7. Integrity test results for samples AN21_fin (top), AN20_fin (middle, failed eQC) and AN19_fin (bottom).**


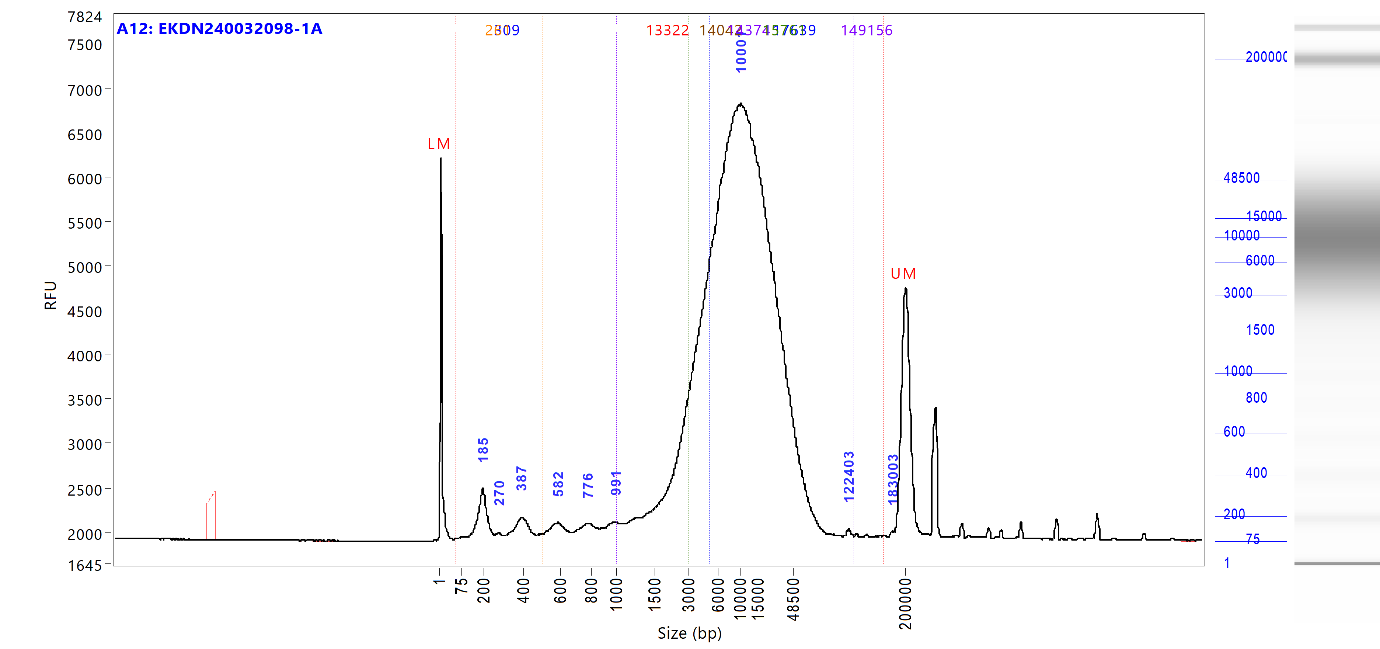

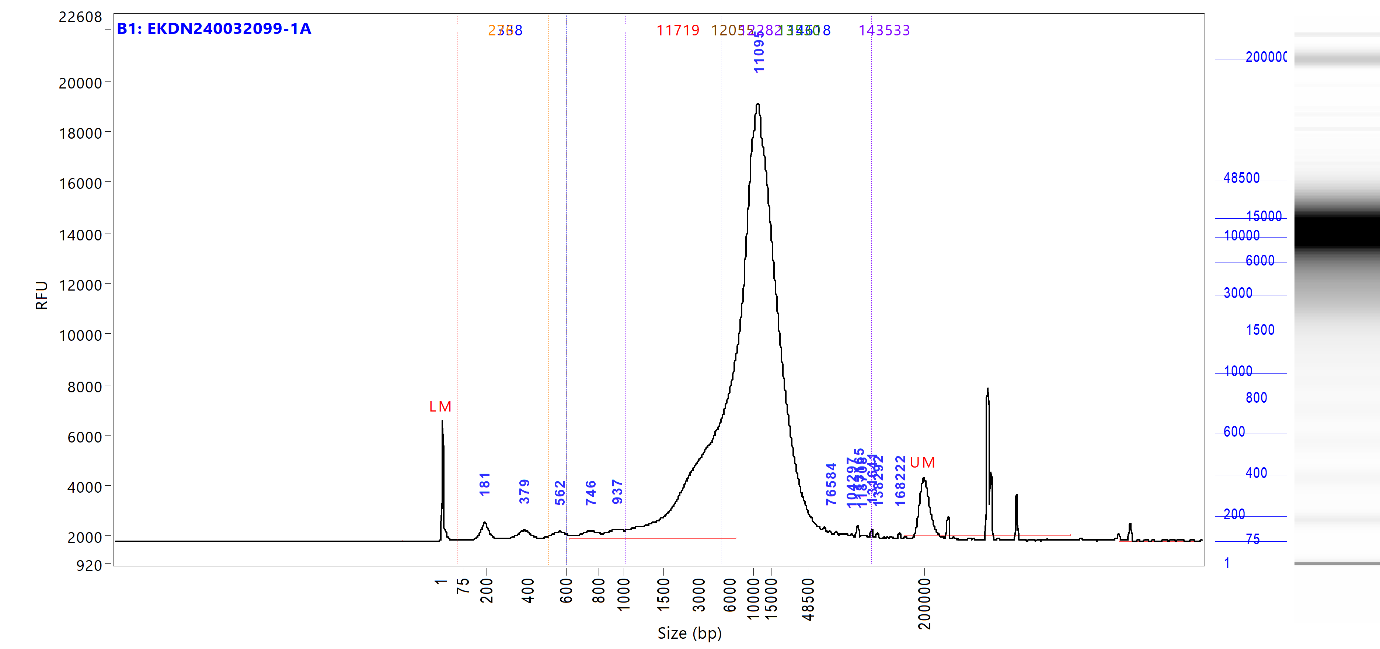

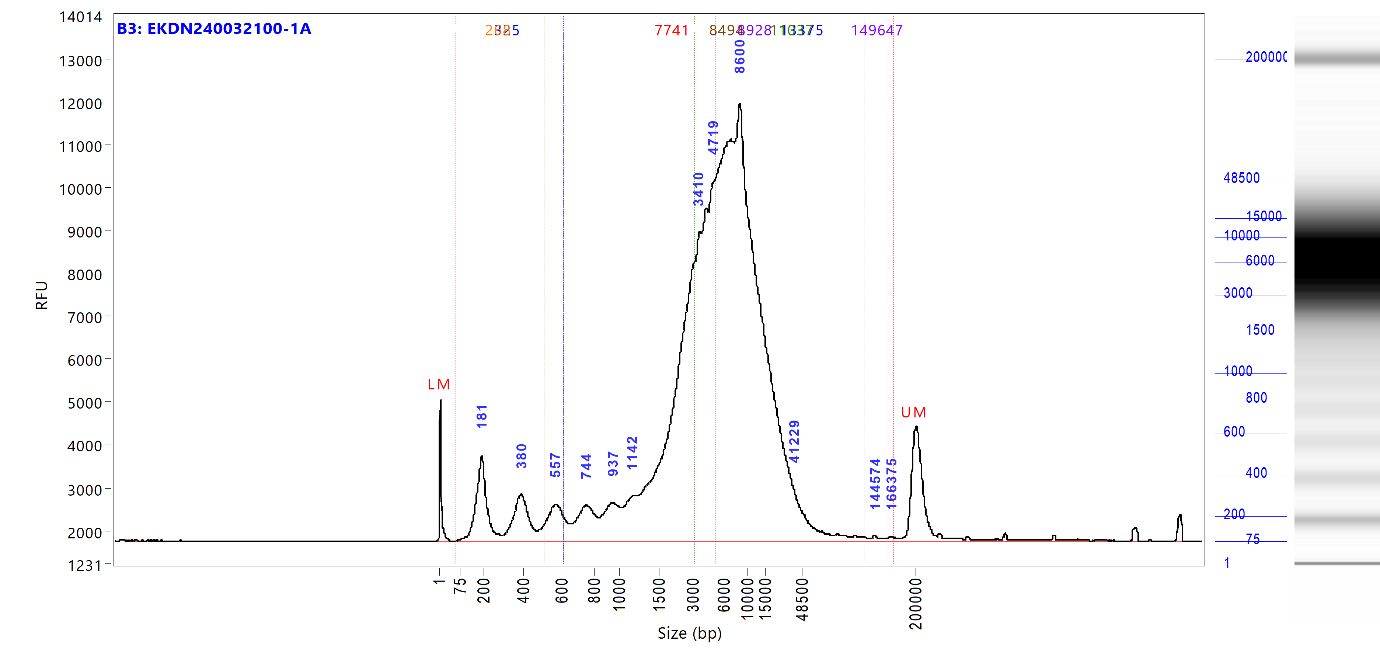


**Supplementary Figure 8. Integrity test results for samples AN18_fin (top), AN17_fin (middle) and AN16_fin (bottom, failed eQC).**


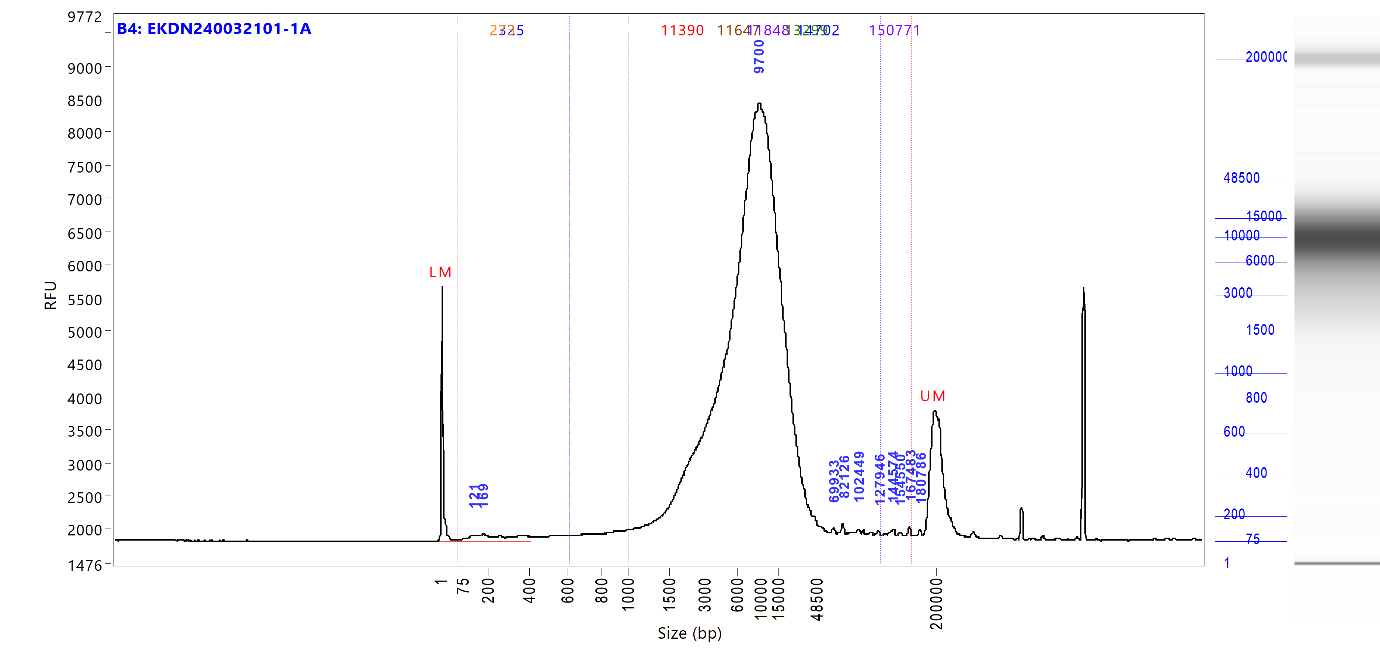

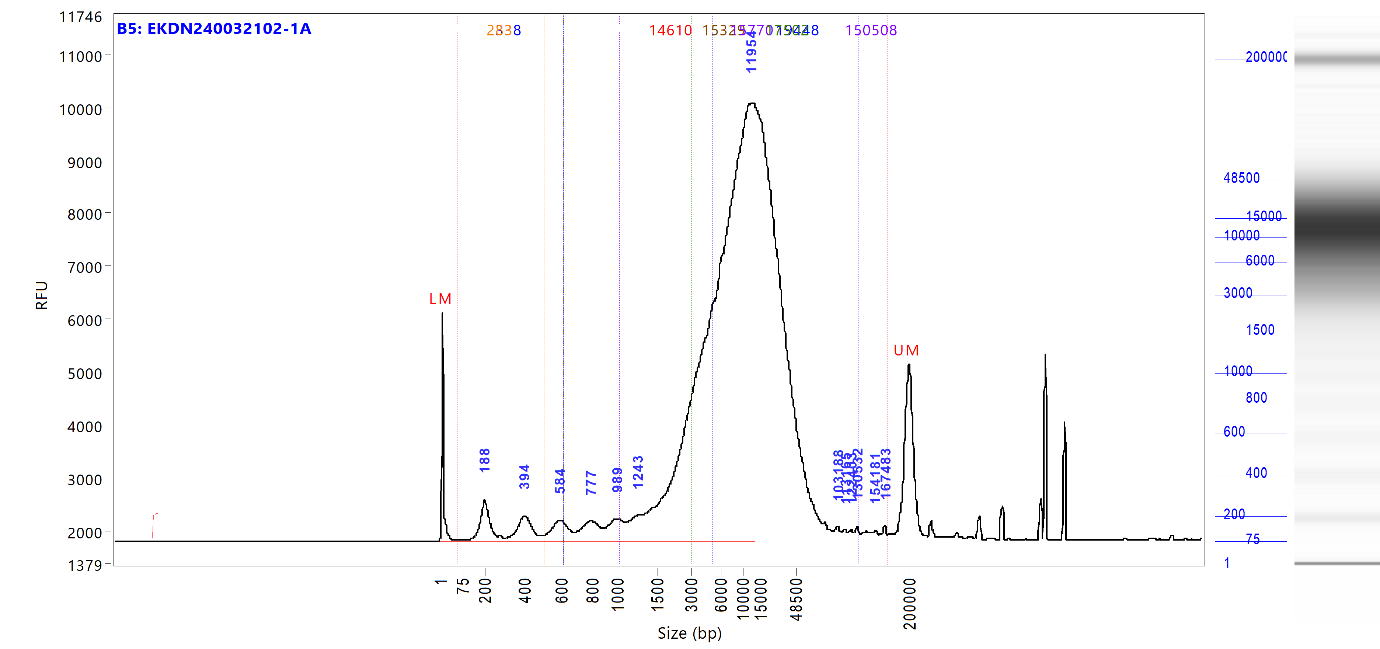

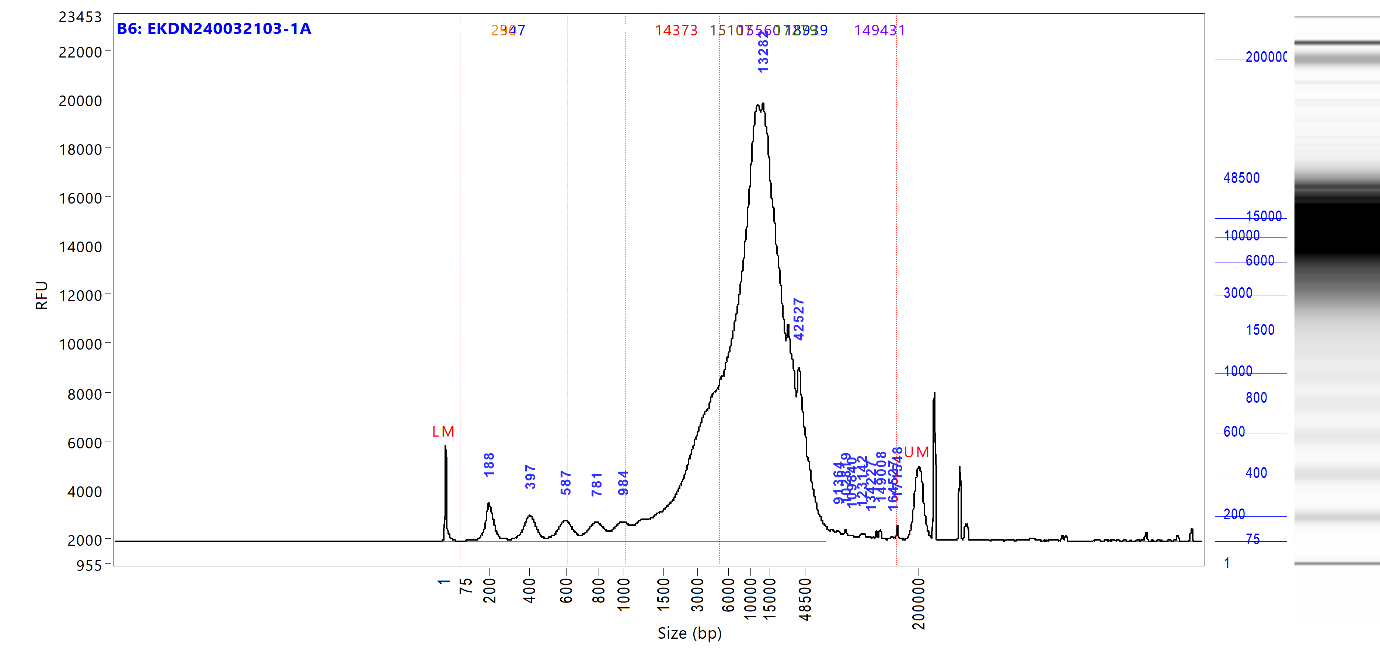


**Supplementary Figure 9. Integrity test results for samples AN15_fin (top), AN14_fin (middle) and AN13_fin (bottom).**


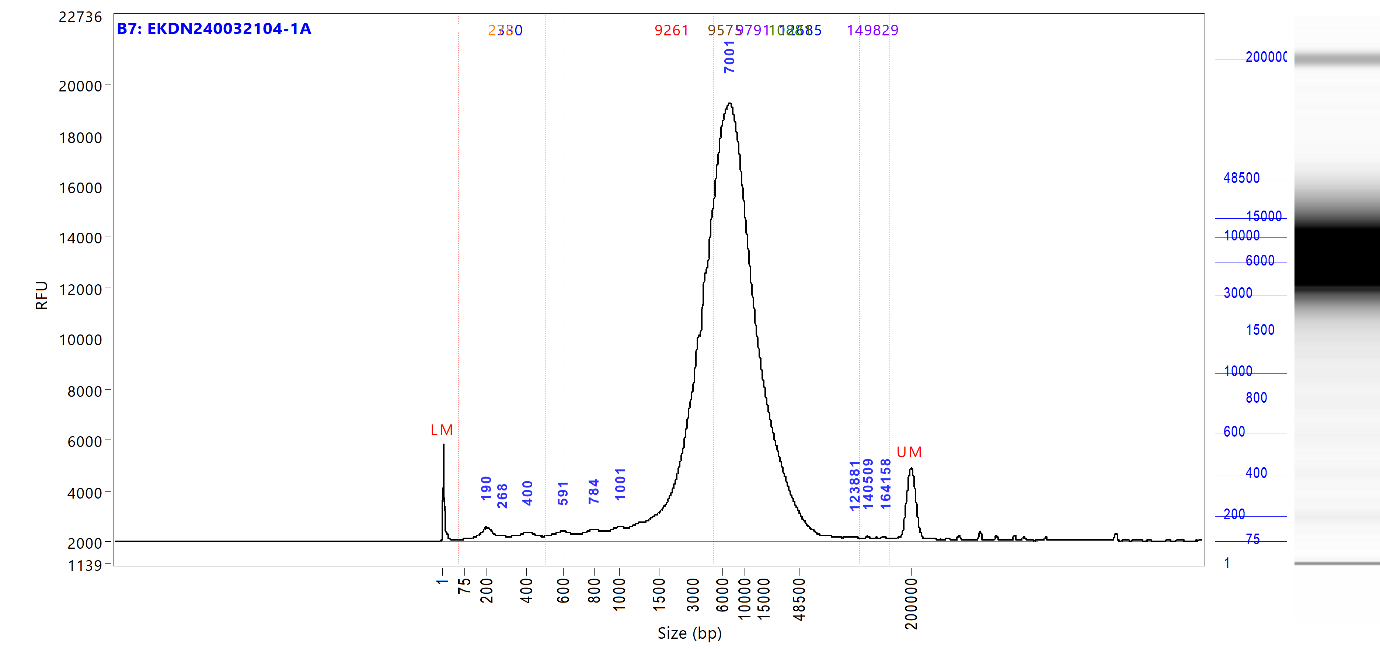

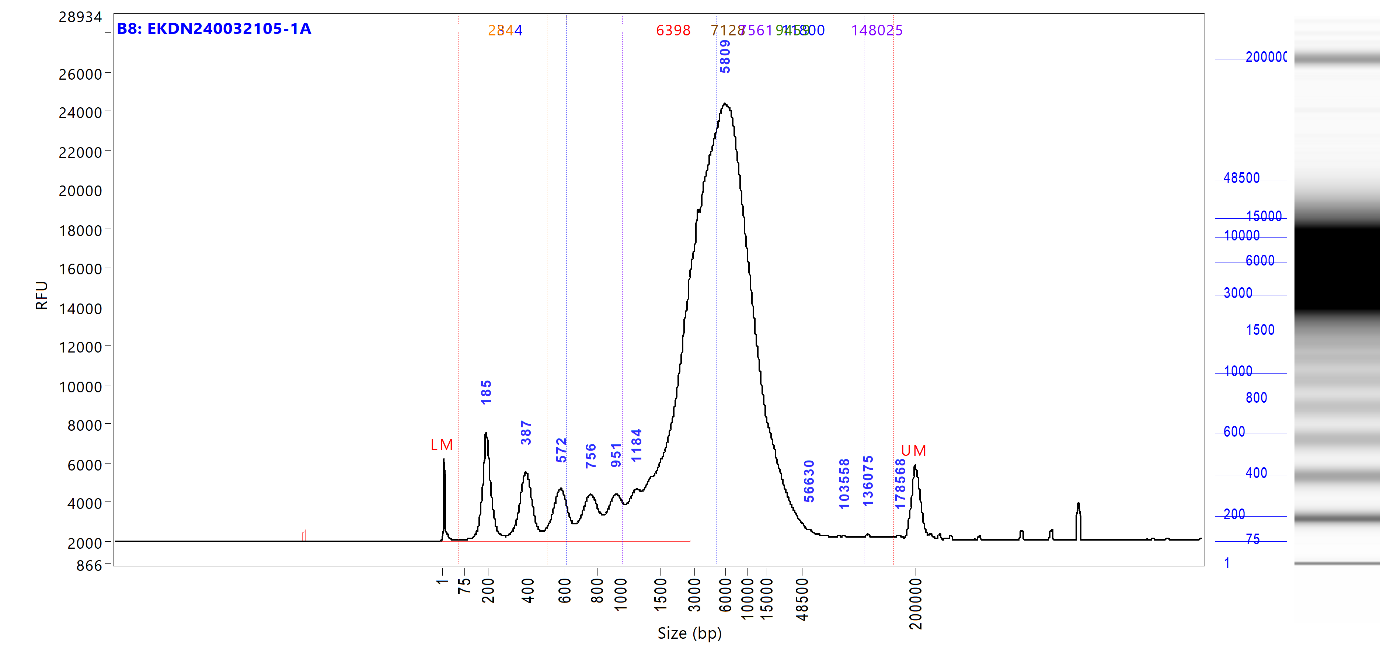

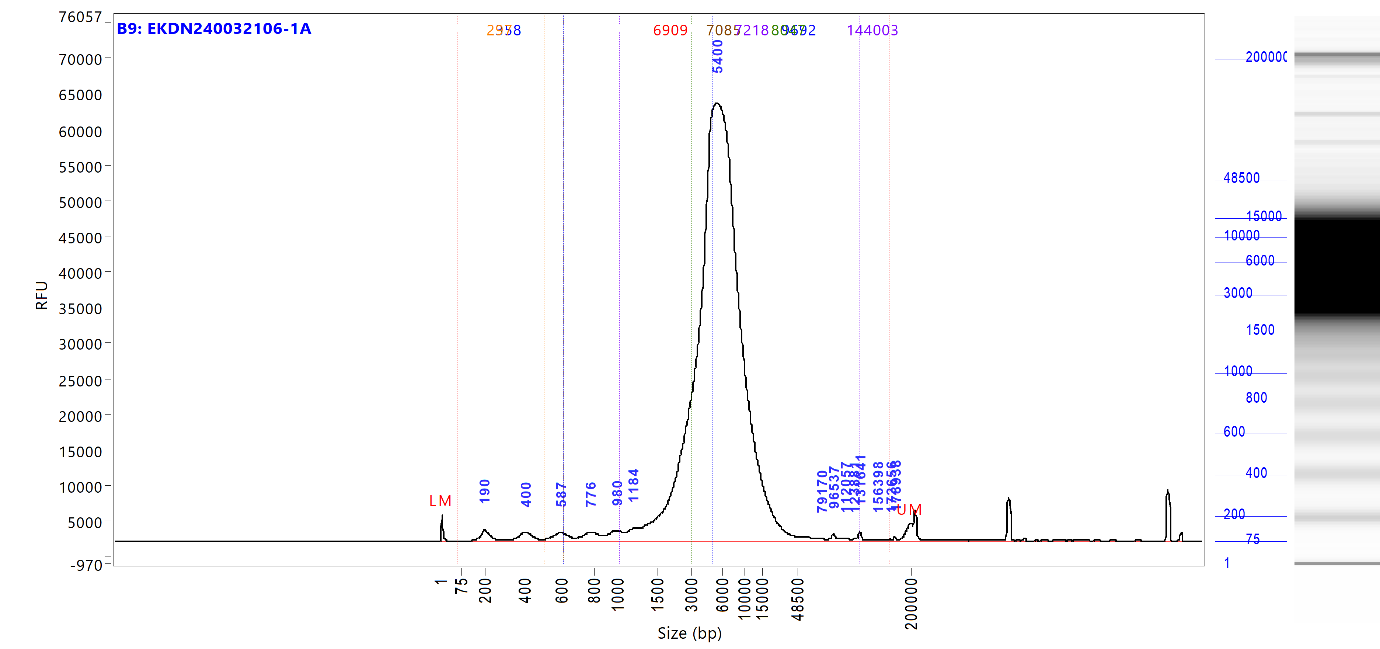


**Supplementary Figure 10. Integrity test results for samples AN12_fin (top), AN11_fin (middle, failed eQC) and AN10_fin (bottom, failed eQC).**


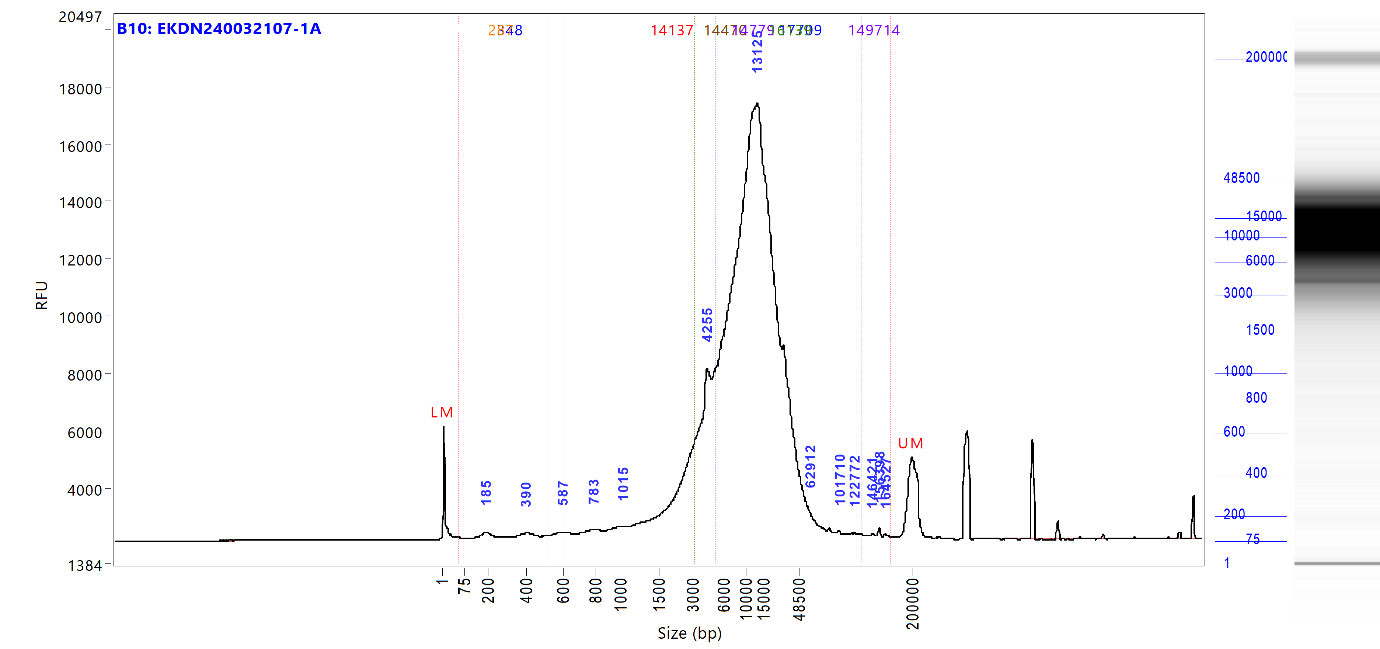

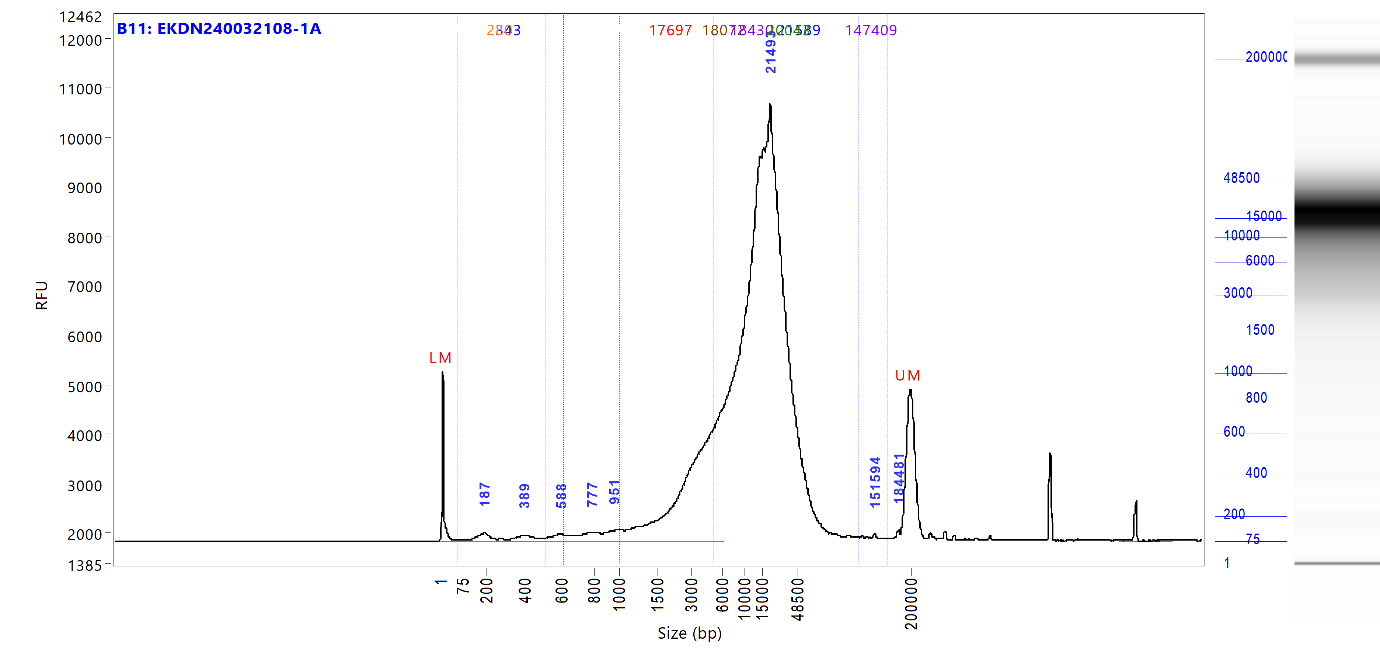

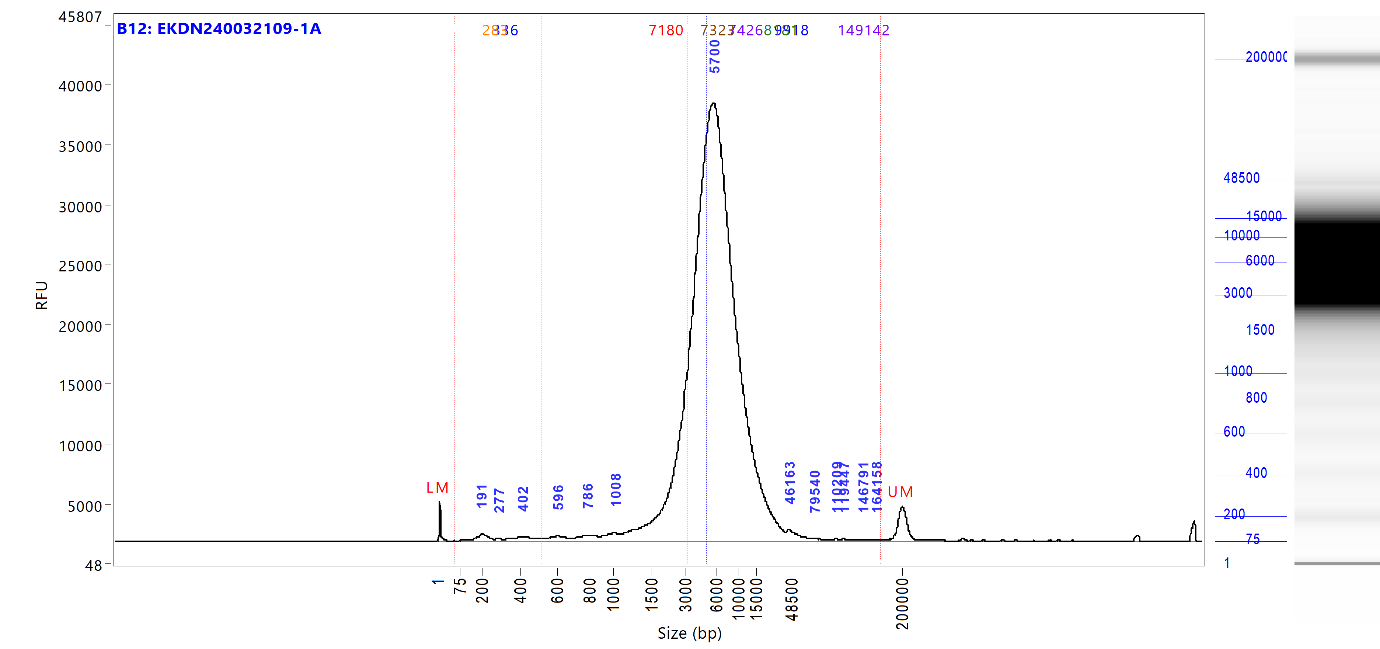


**Supplementary Figure 11. Integrity test results for samples AN09_fin (top), AN08_fin (middle) and AN07_fin (bottom).**


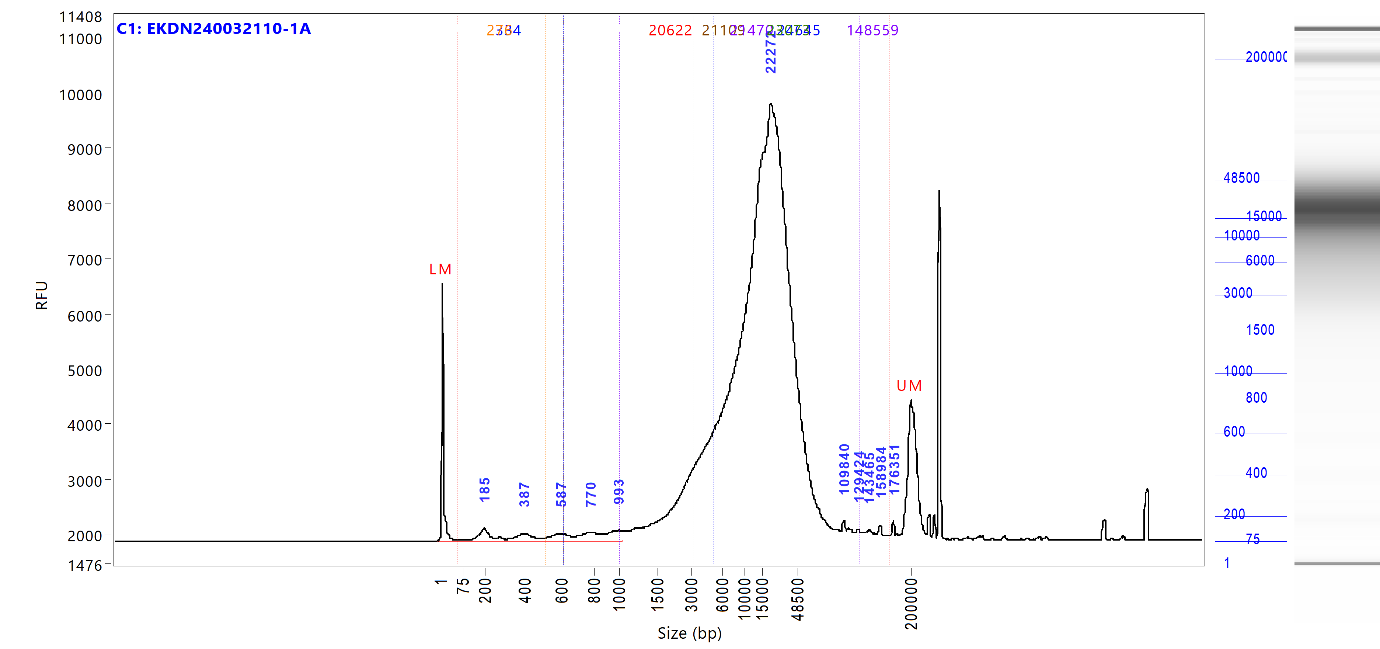

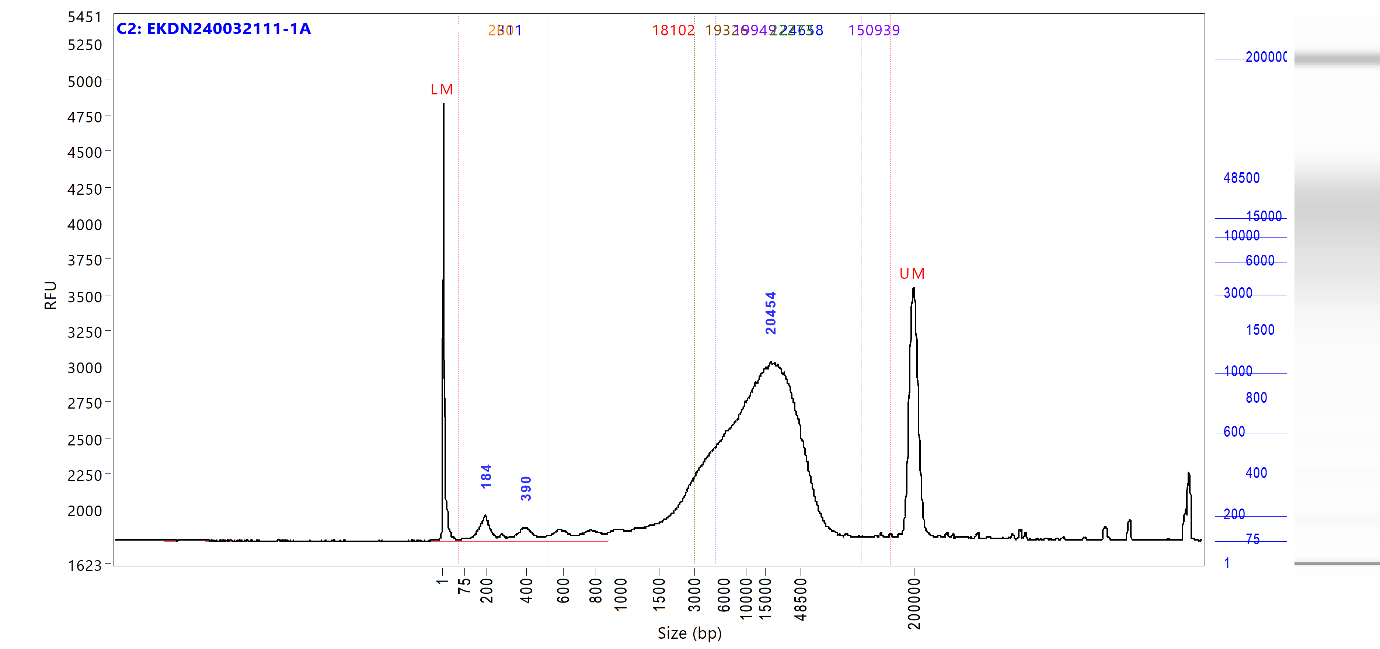

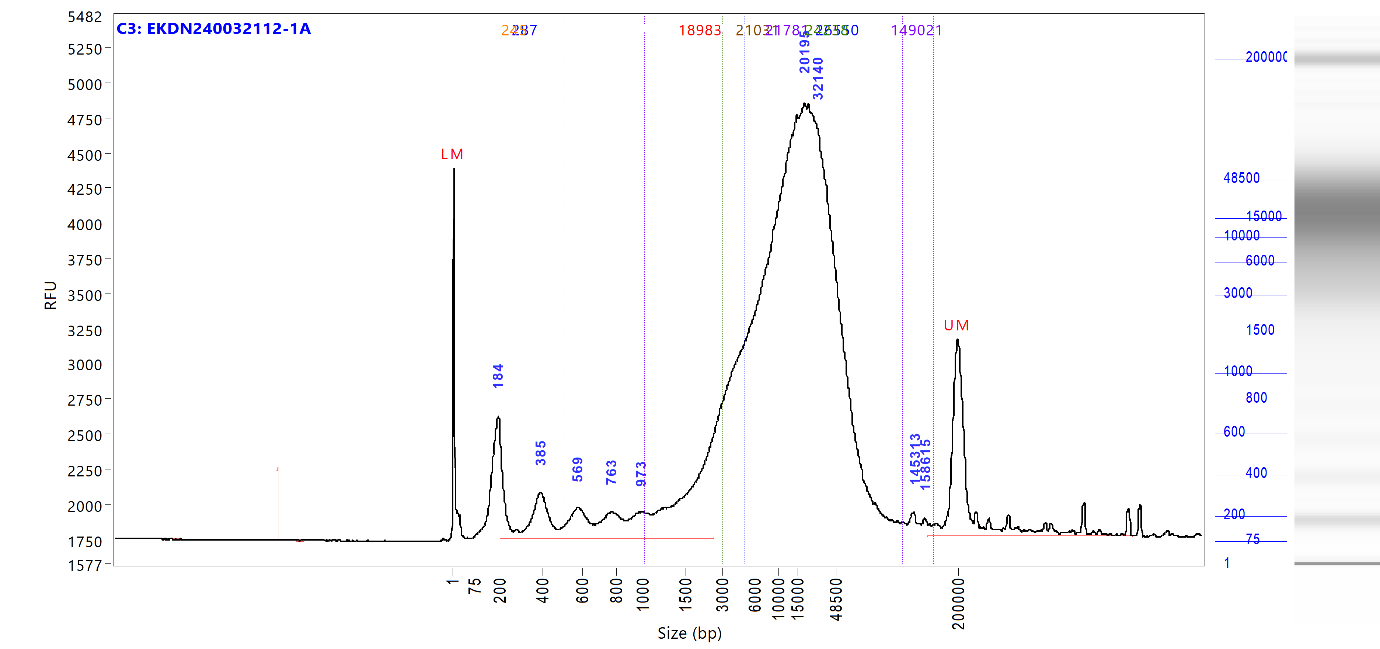


**Supplementary Figure 12. Integrity test results for samples AN06_fin (top), AN05_fin (middle, failed eQC) and AN04_fin (bottom).**


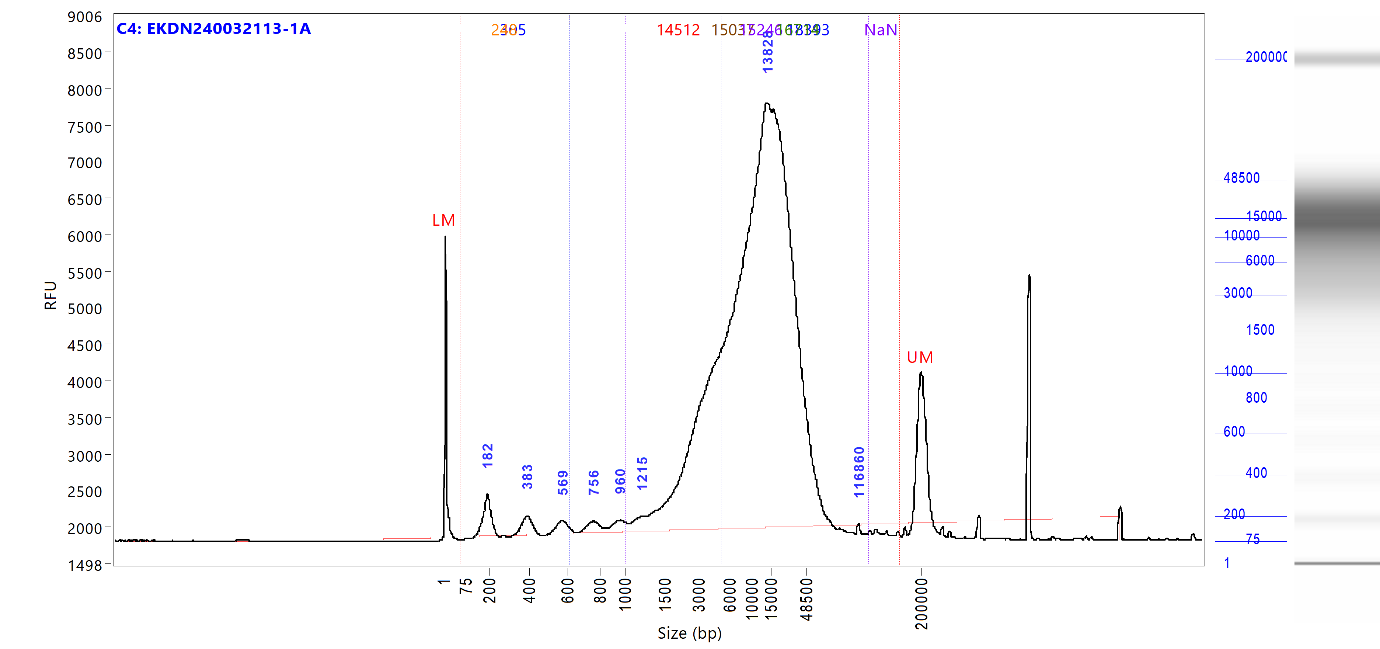

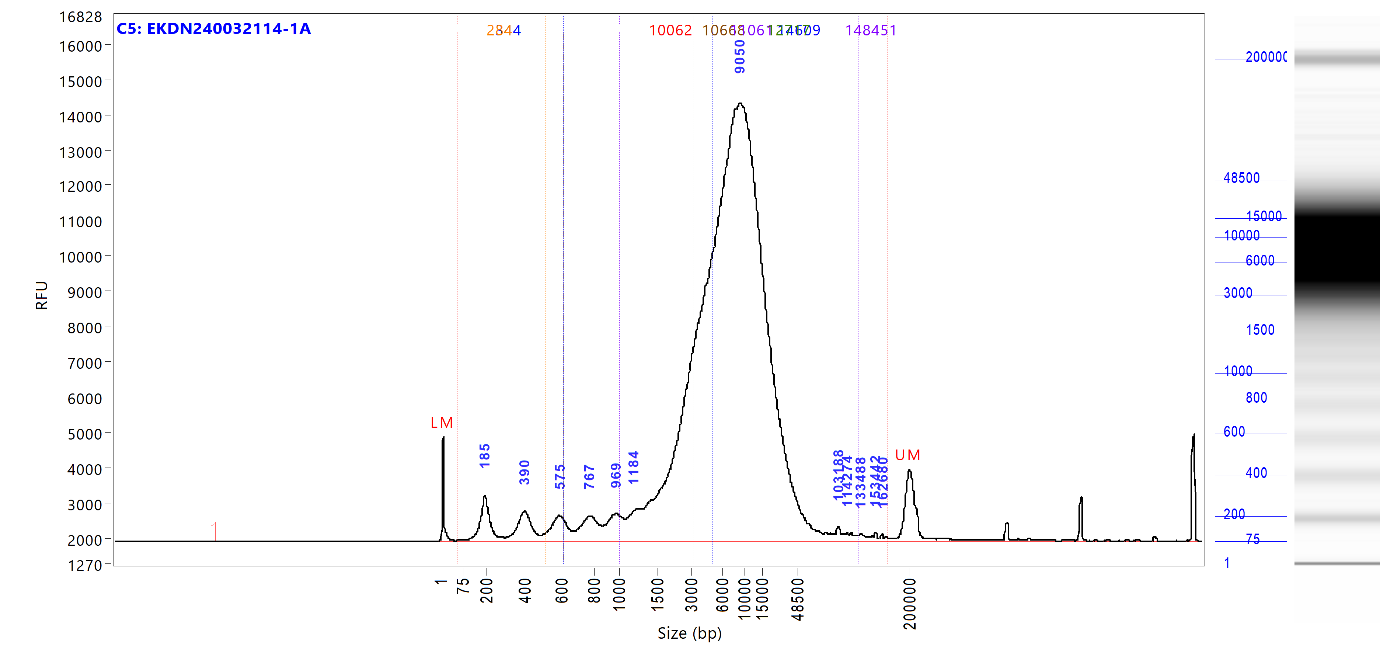

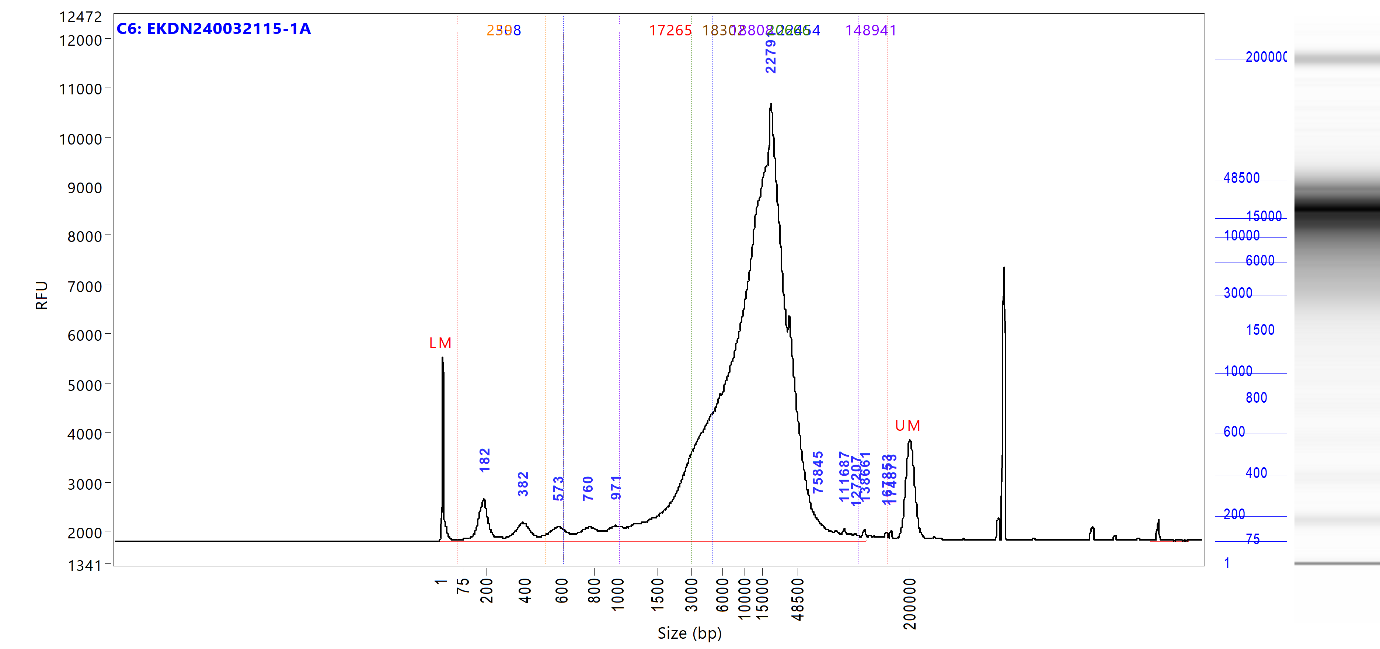


**Supplementary Figure 13. Integrity test results for samples AN03_fin (top), AN02_fin (middle) and AN01_fin (bottom).**


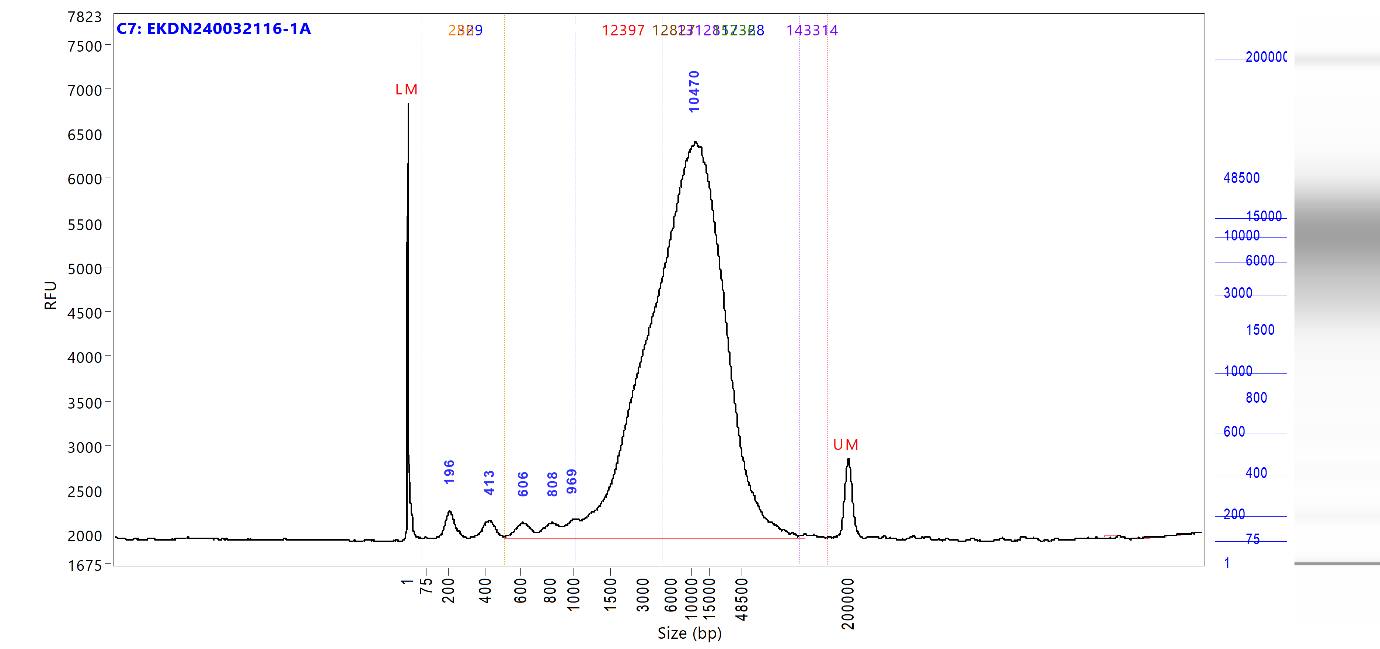

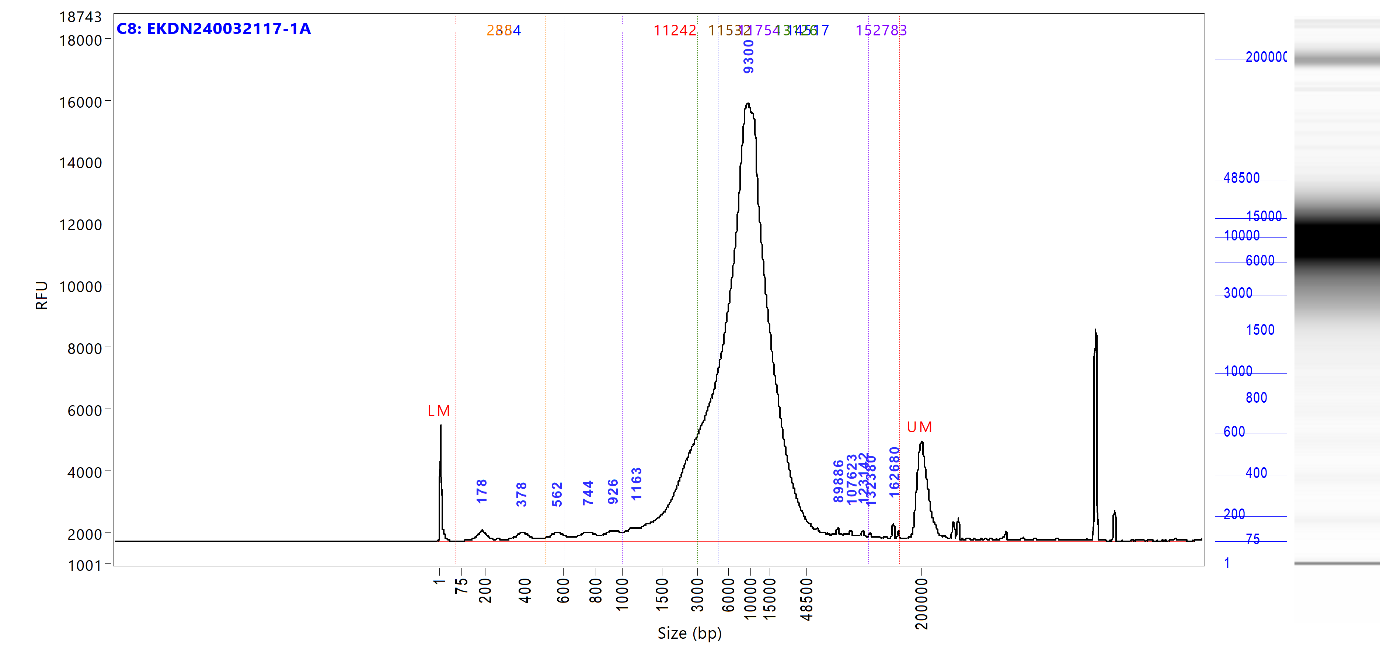

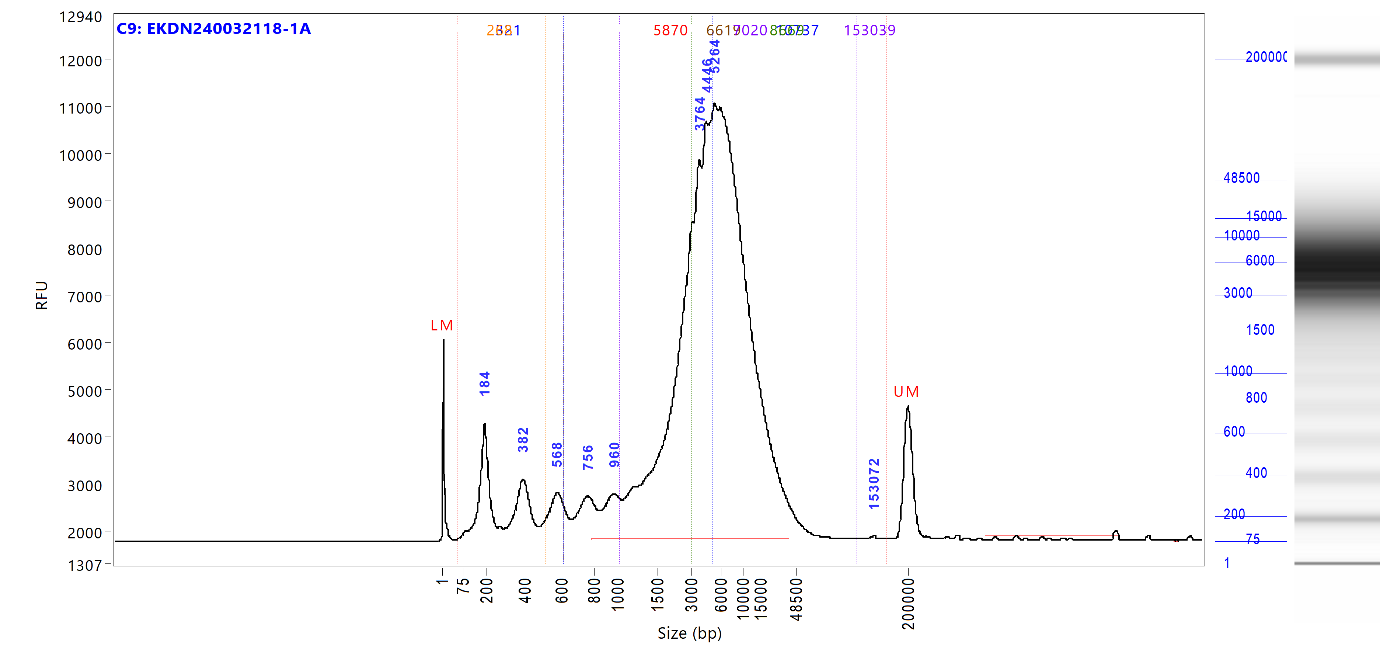


**Supplementary Figure 14. Integrity test results for samples AN00_fin (top), AM99_fin (middle) and AM98_fin (bottom, failed eQC).**


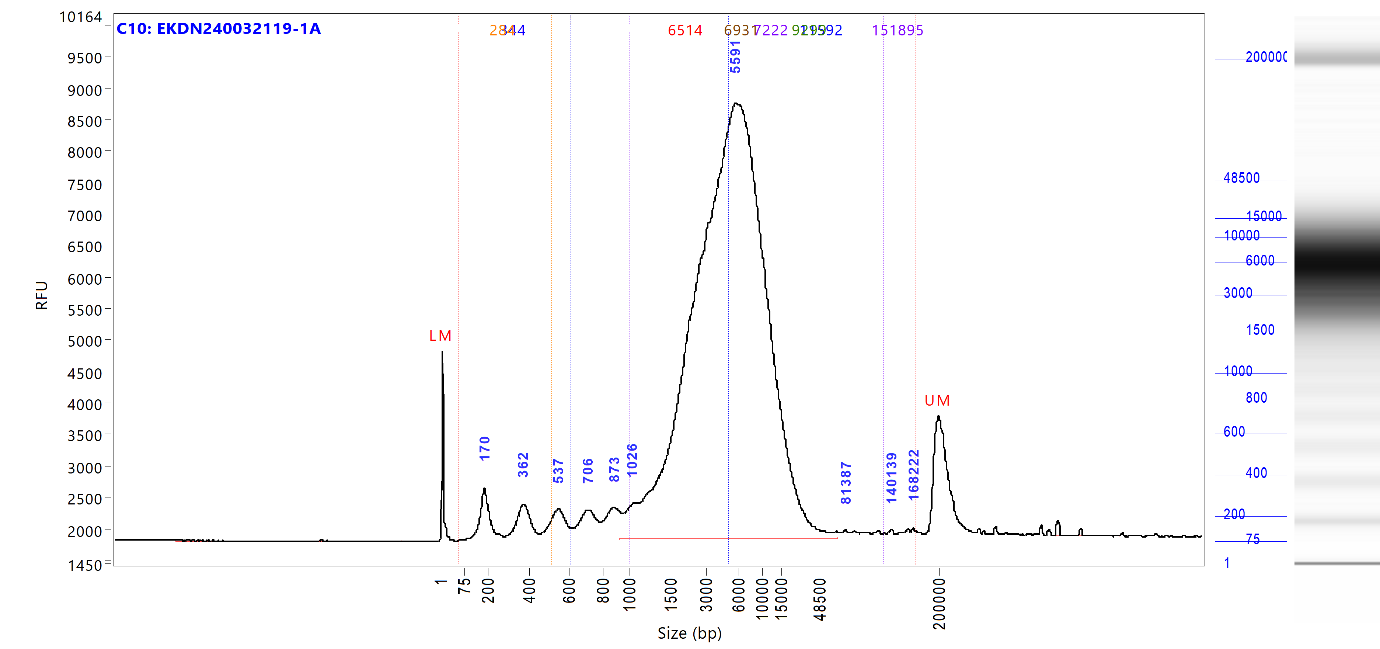

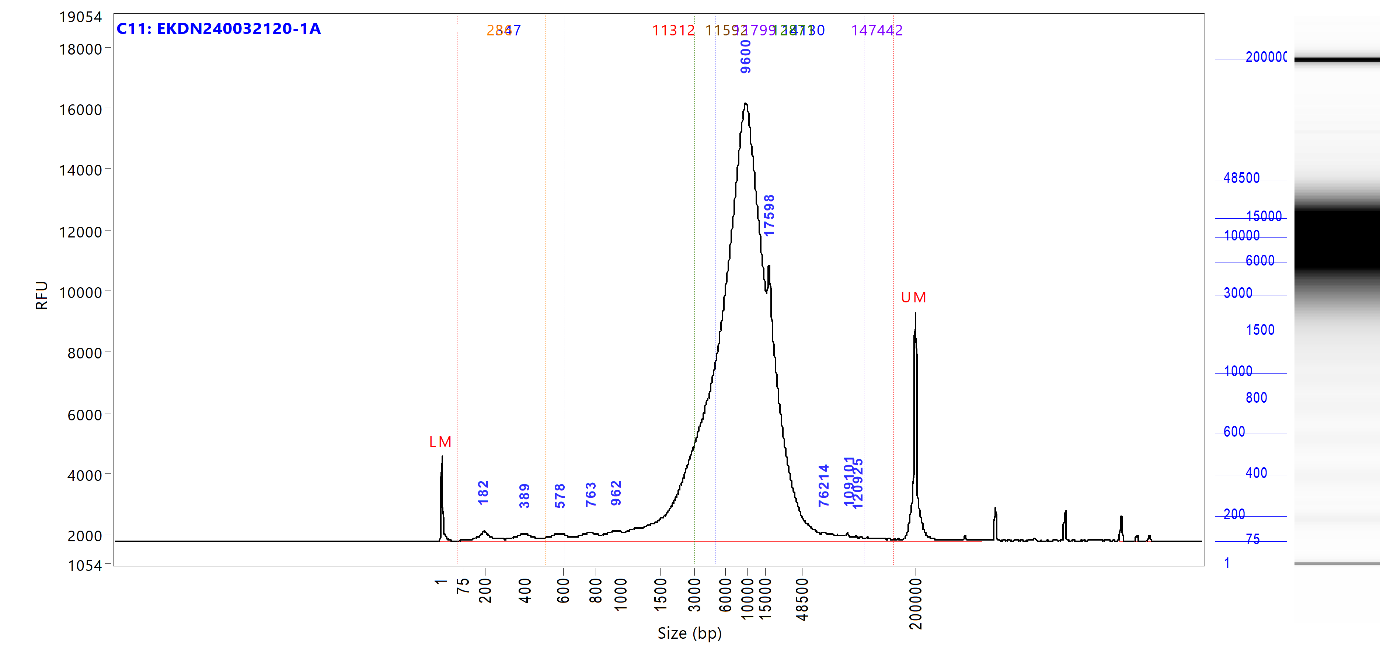

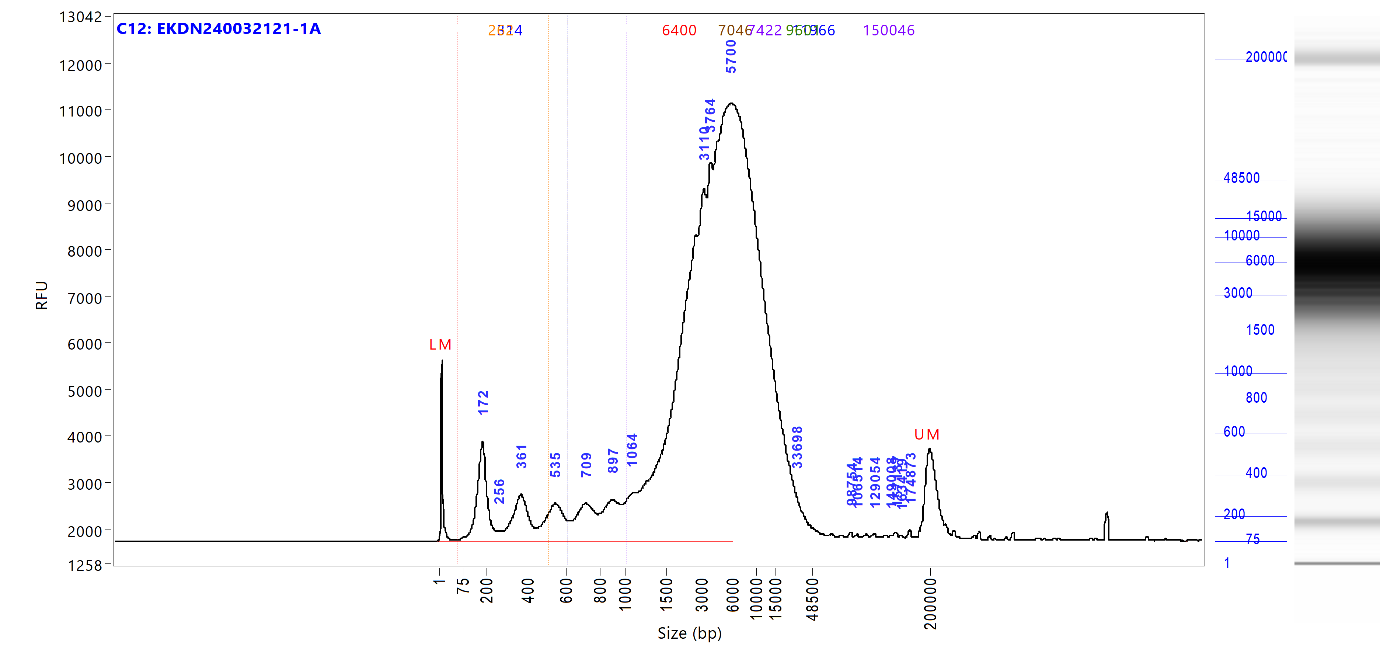


**Supplementary Figure 15. Integrity test results for samples AM97_fin (top, failed eQC), AM96_fin (middle) and AM95_fin (bottom, failed eQC).**


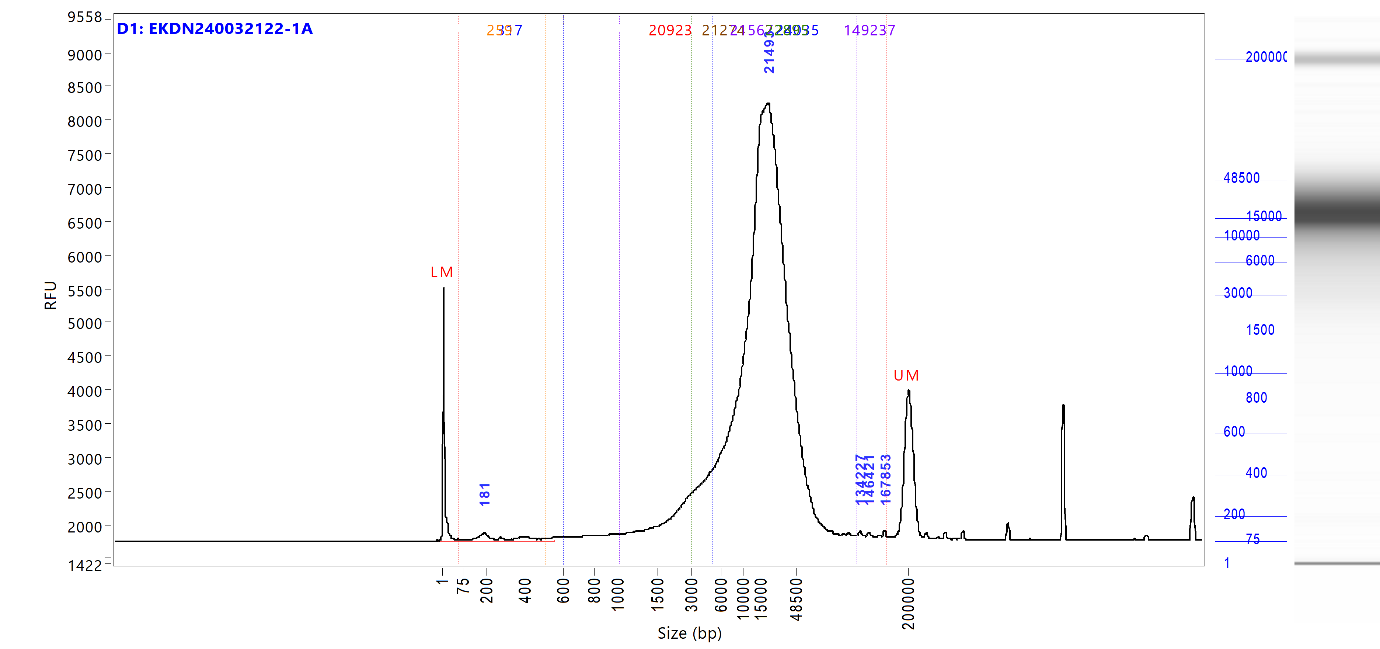

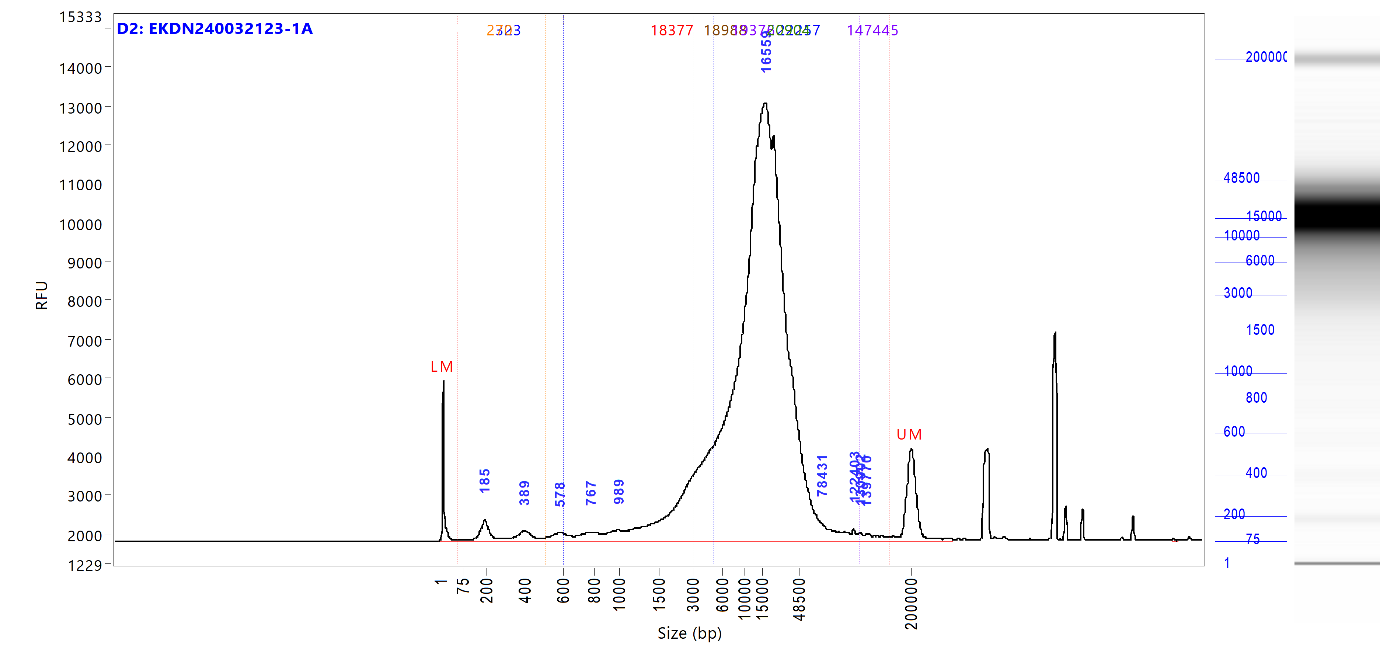

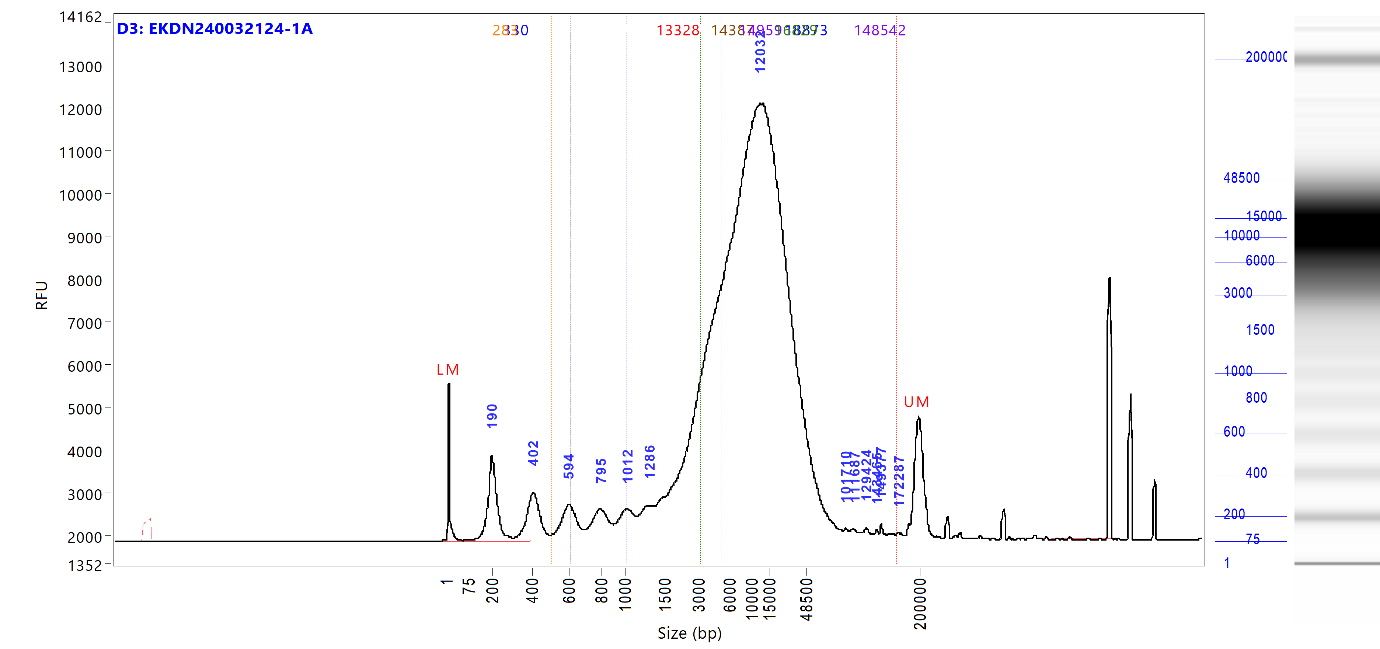


**Supplementary Figure 16. Integrity test results for samples AM94_fin (top), AM93_fin (middle) and AM92_fin (bottom).**


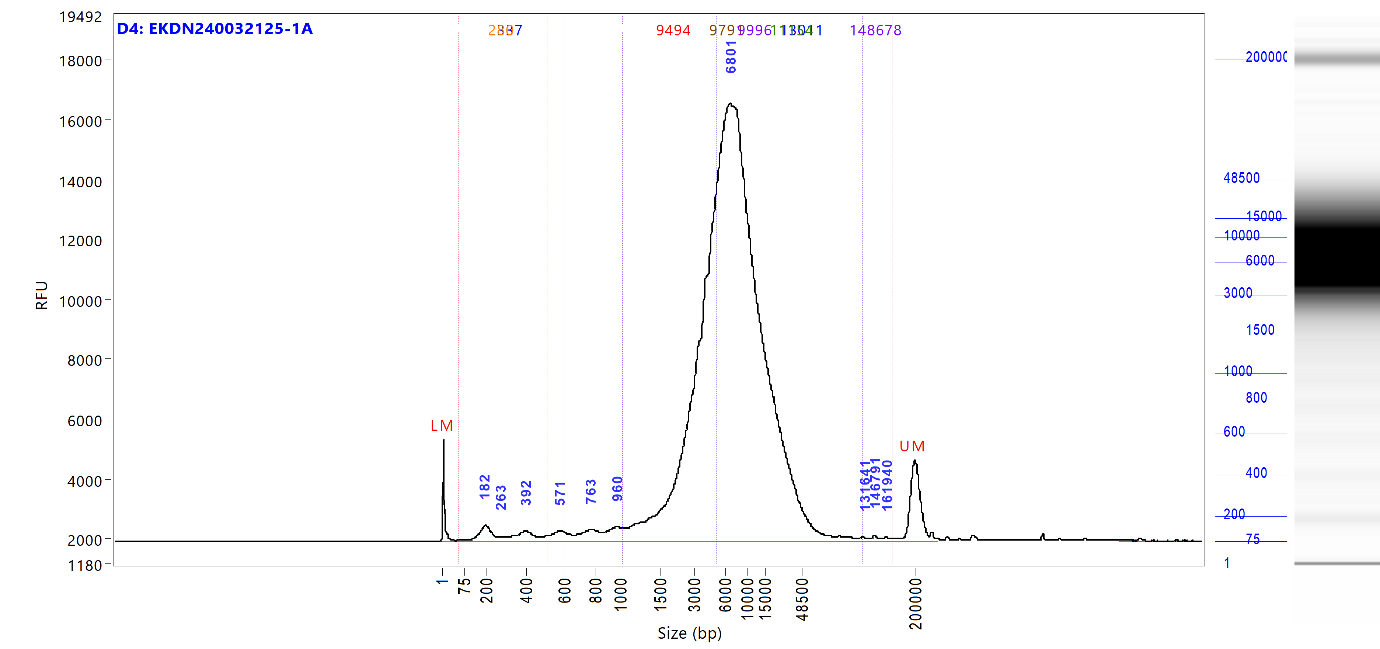

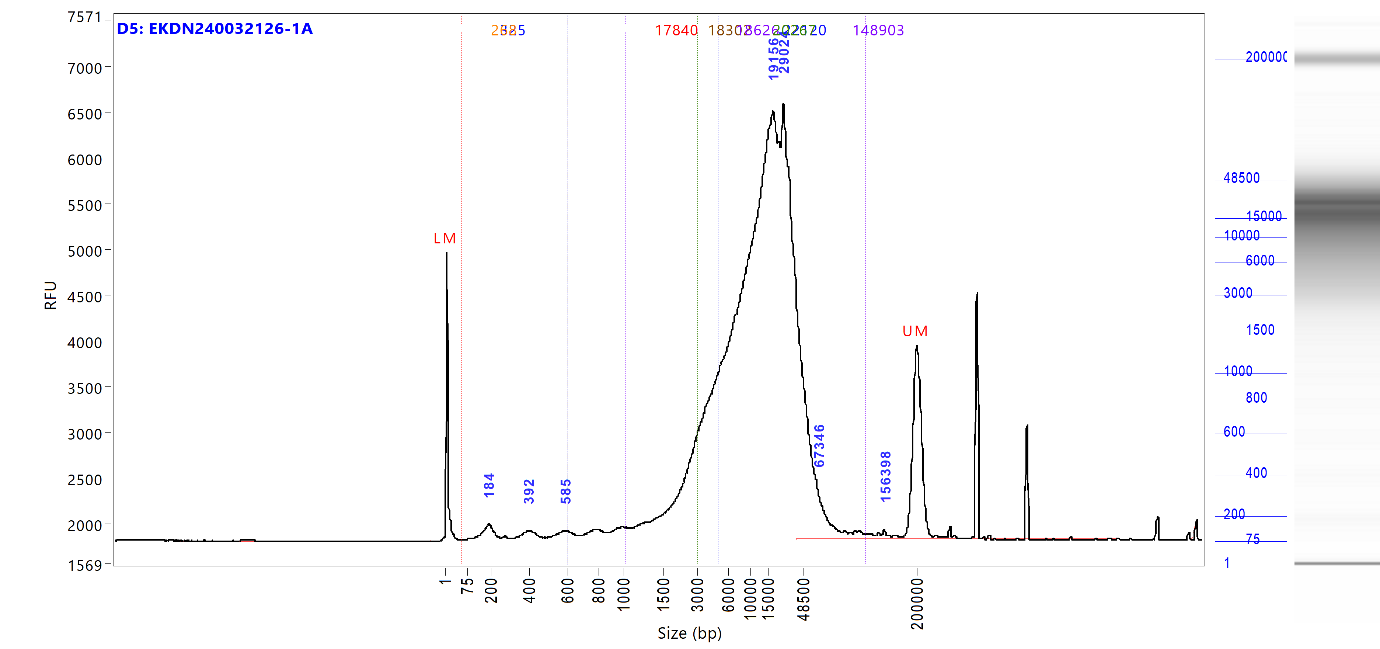

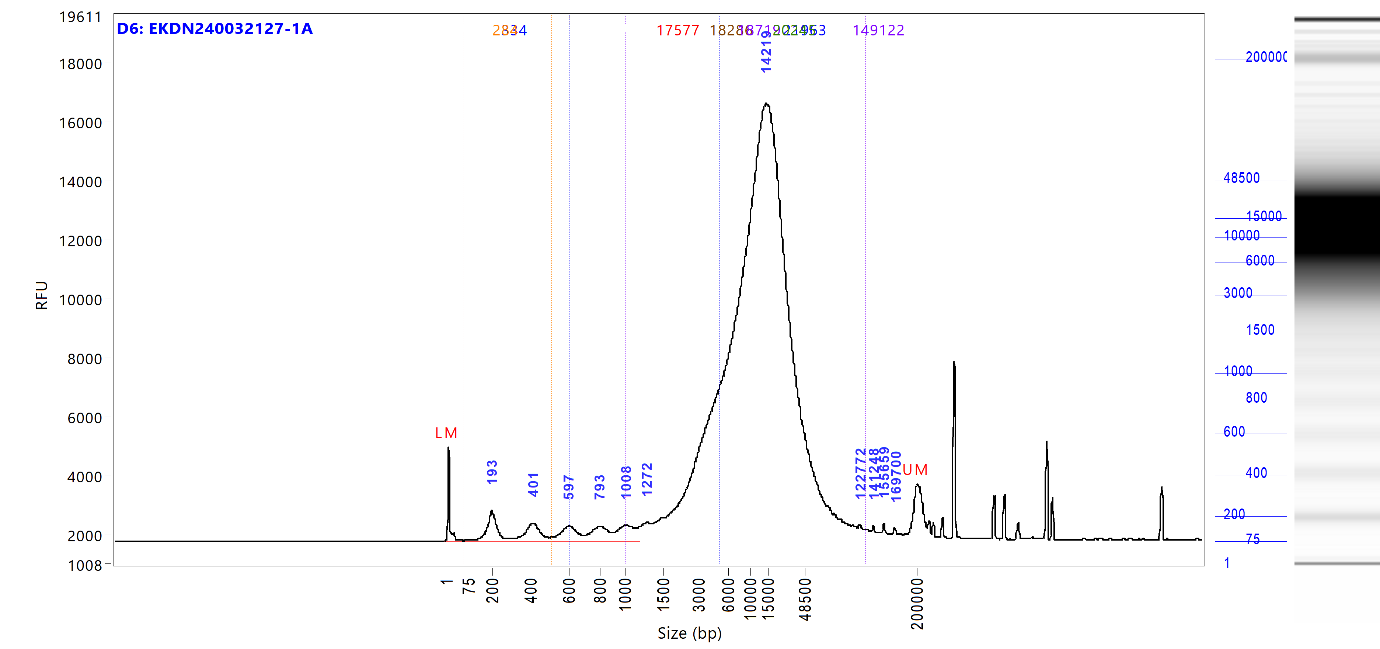


**Supplementary Figure 17. Integrity test results for samples AM91_fin (top), AM90_fin (middle) and AM89_fin (bottom).**


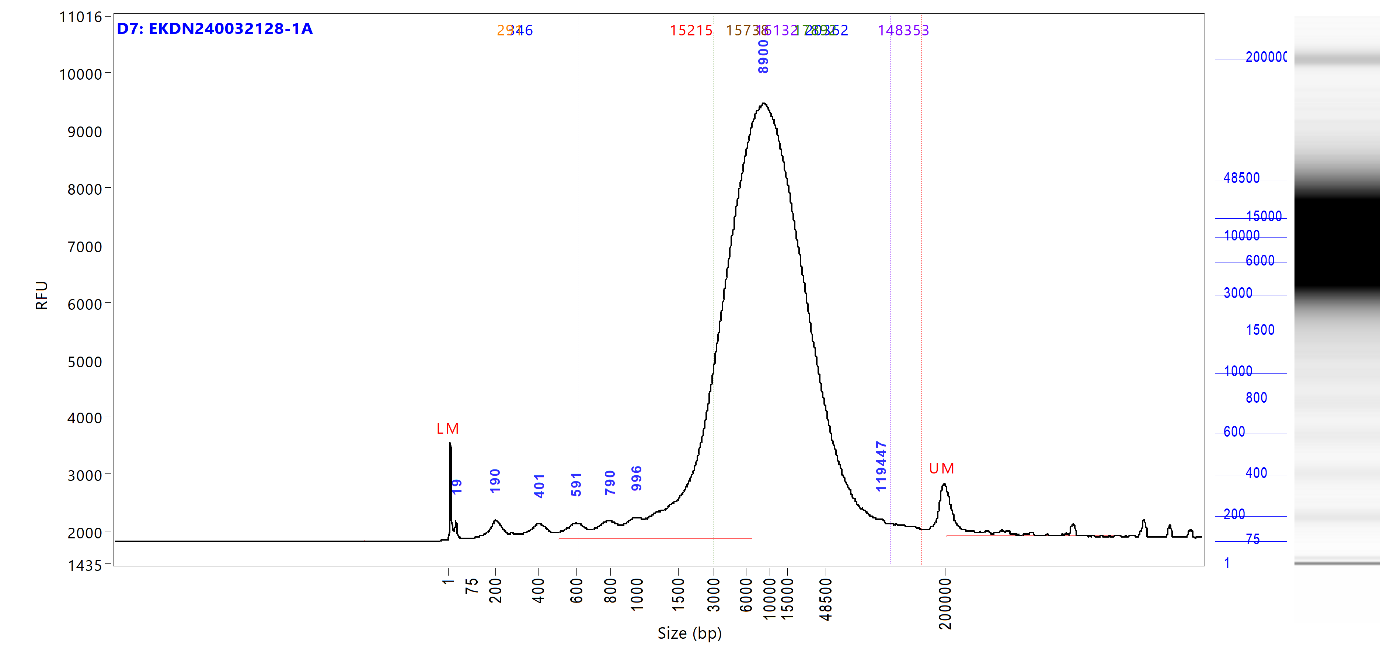

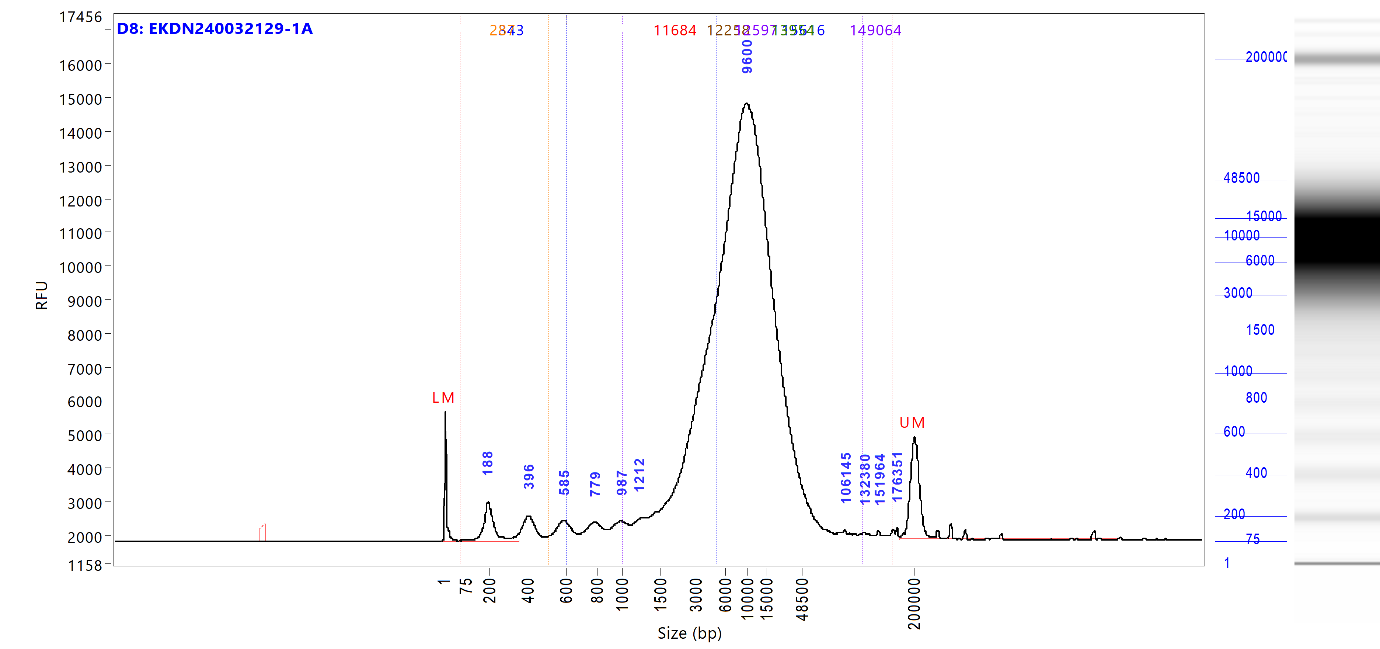

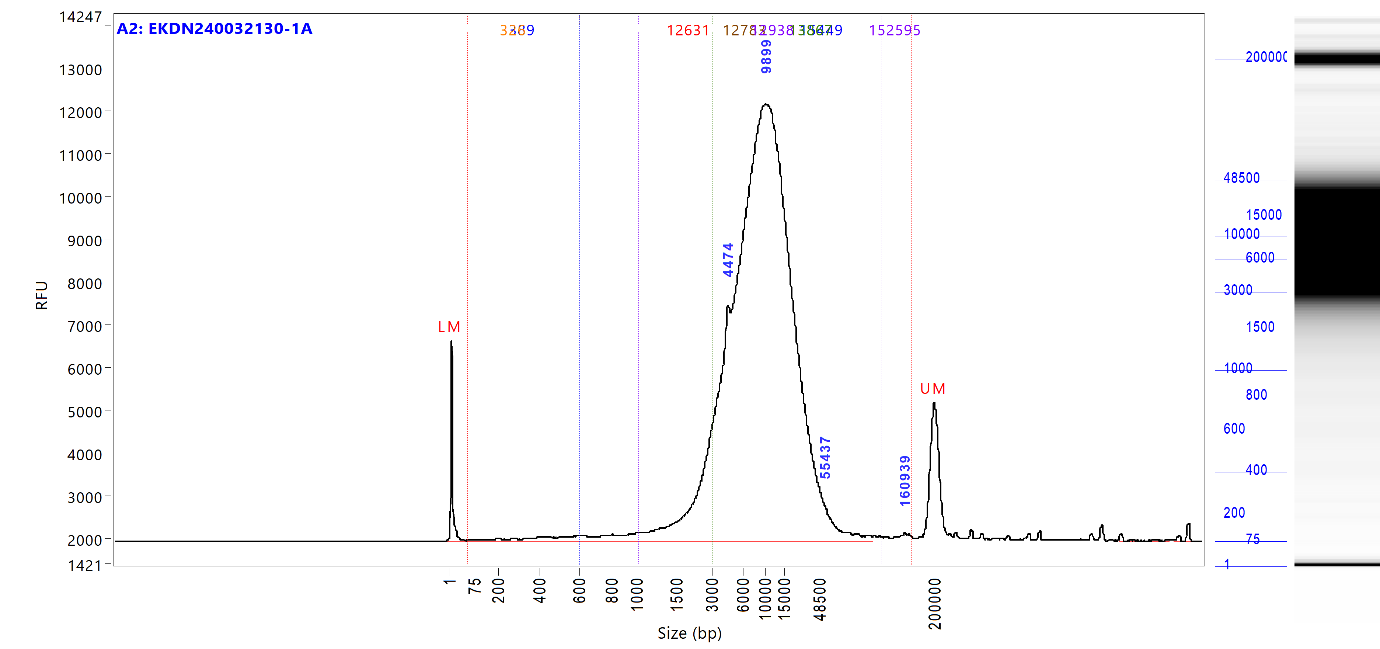


**Supplementary Figure 18. Integrity test results for samples AM88_fin (top), AM87_fin (middle) and AN25_gill (bottom).**


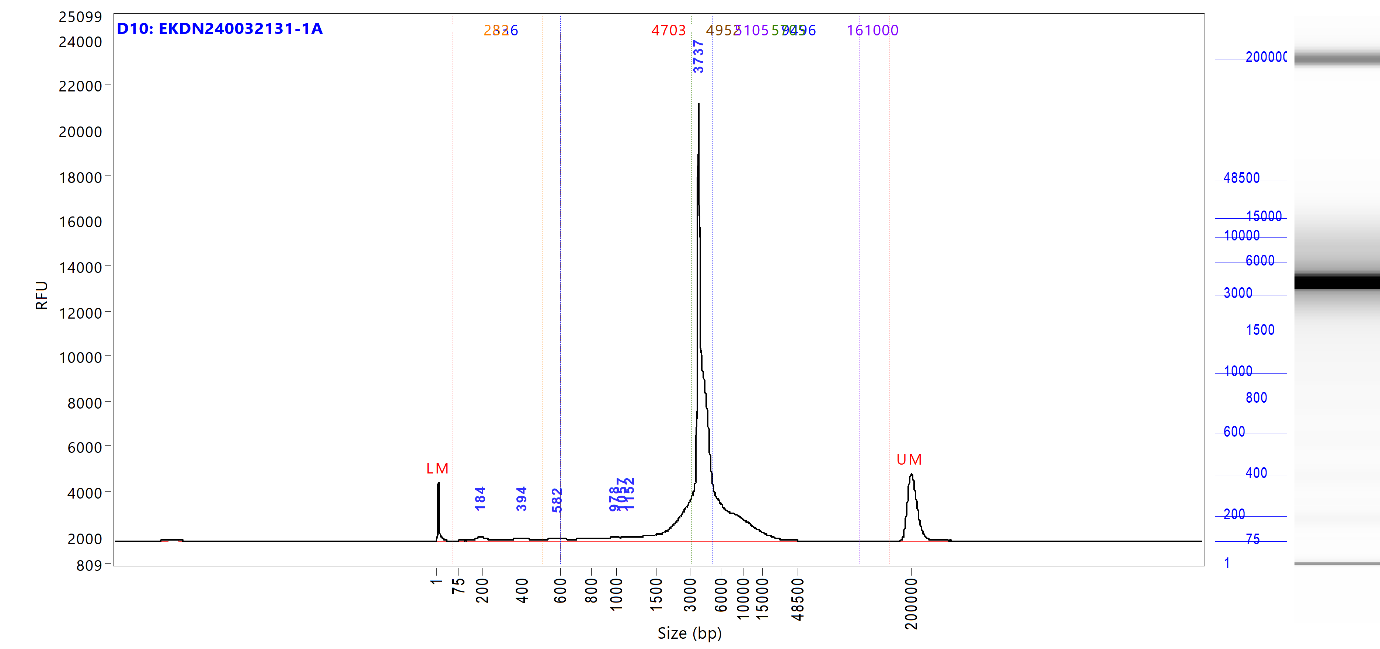

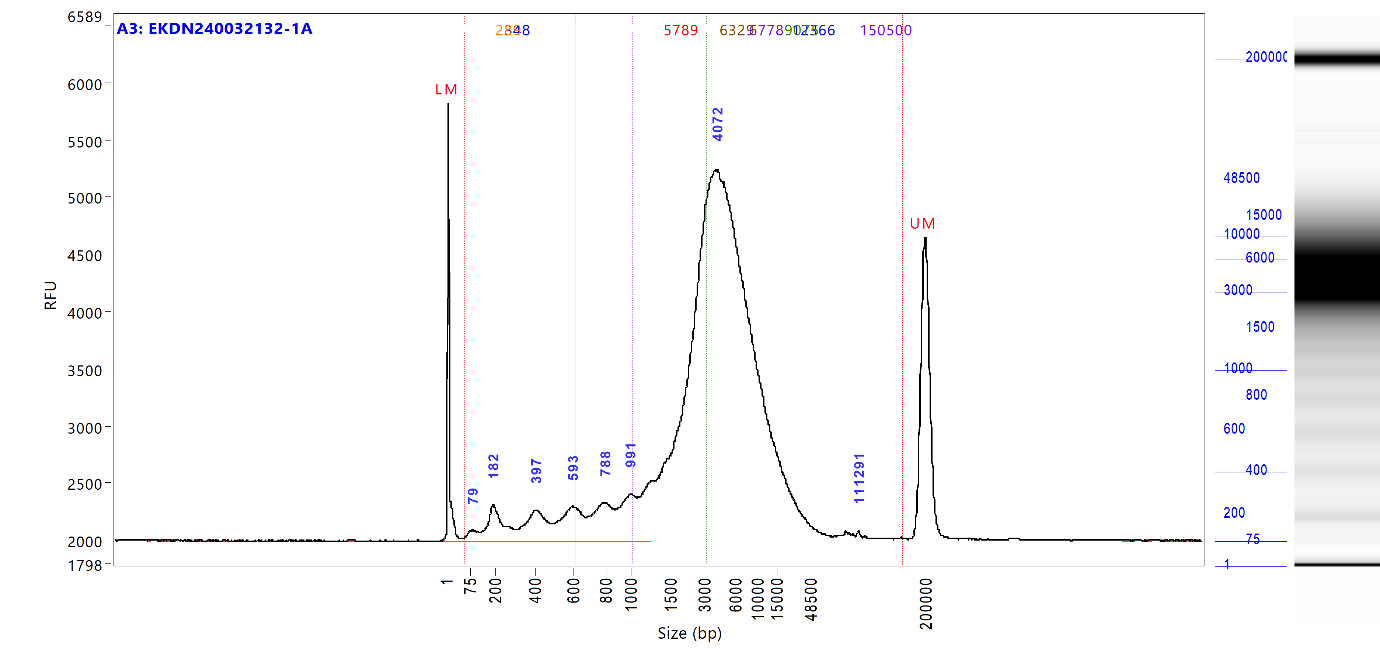

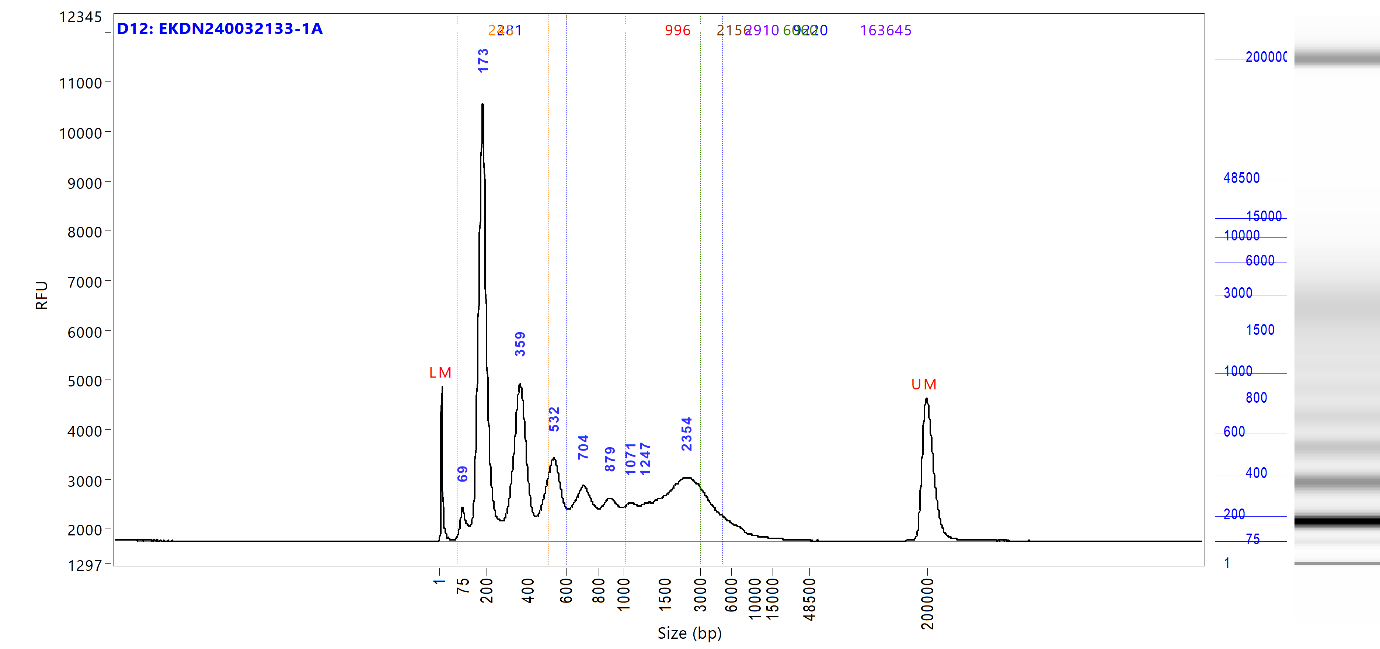


**Supplementary Figure 19. Integrity test results for samples AN25_skin (top, failed eQC), AN24_gill (middle, failed eQC) and AN24_skin (bottom, failed eQC).**


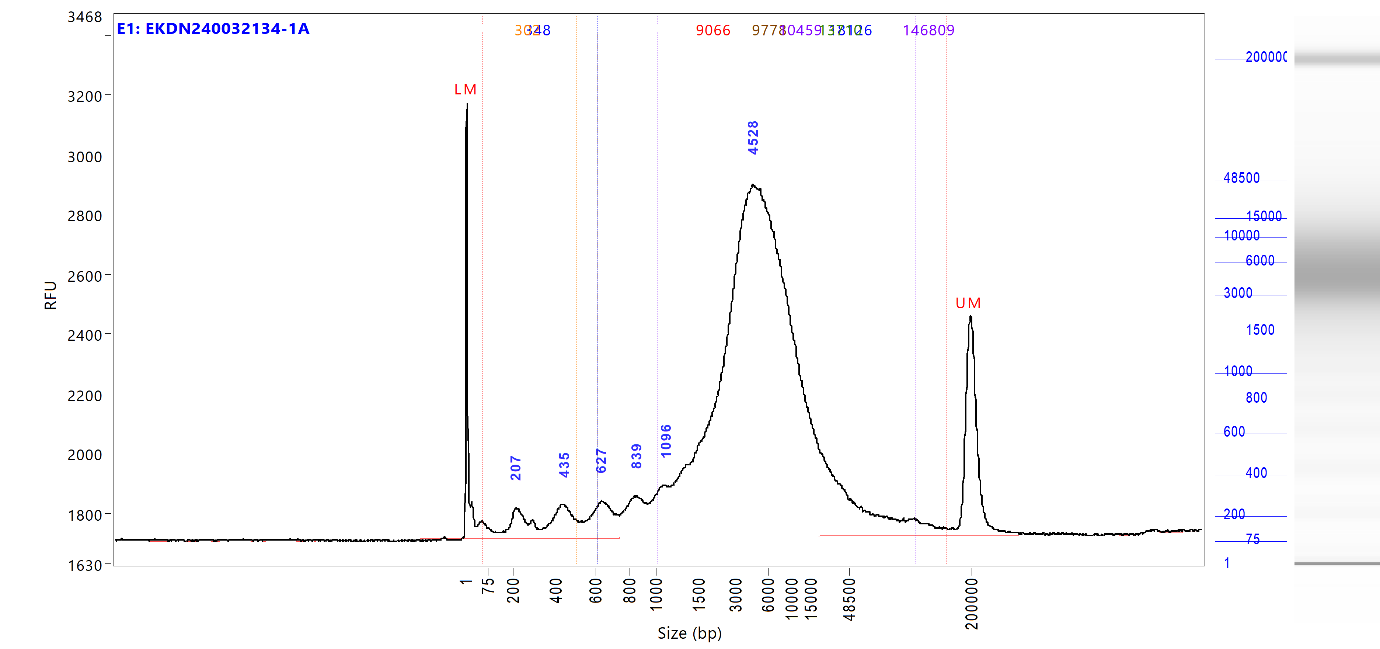

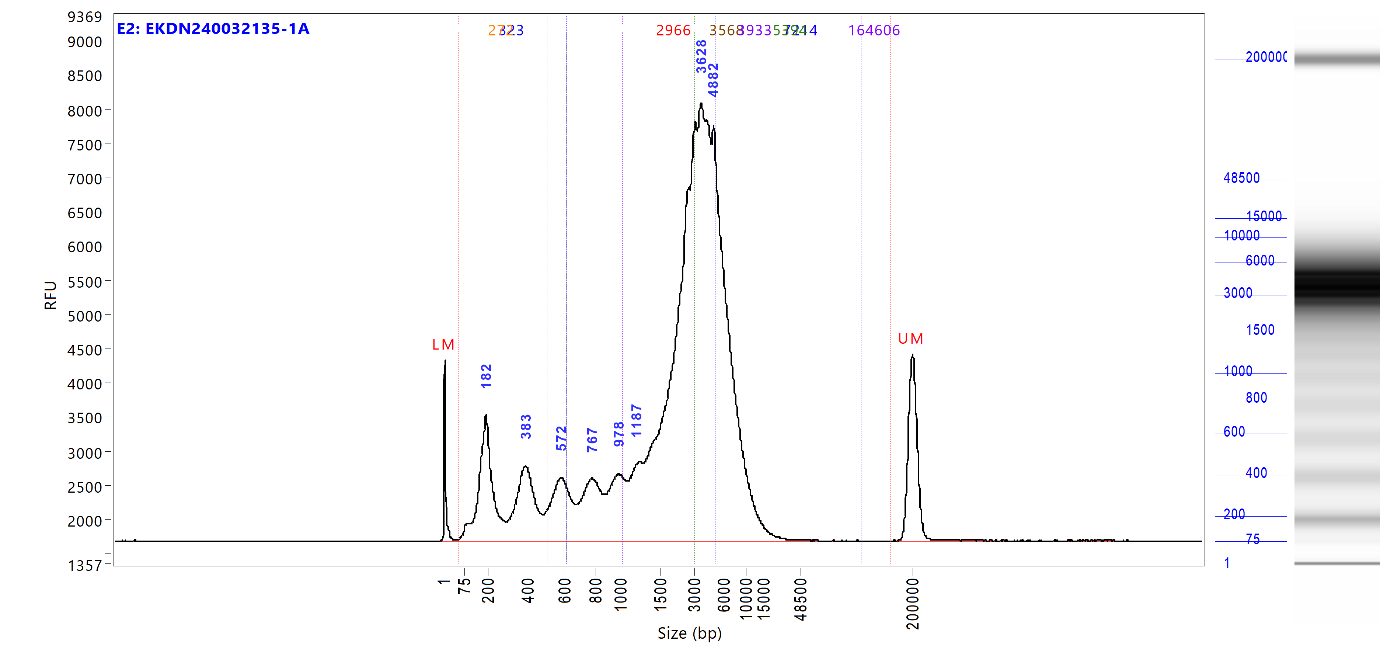

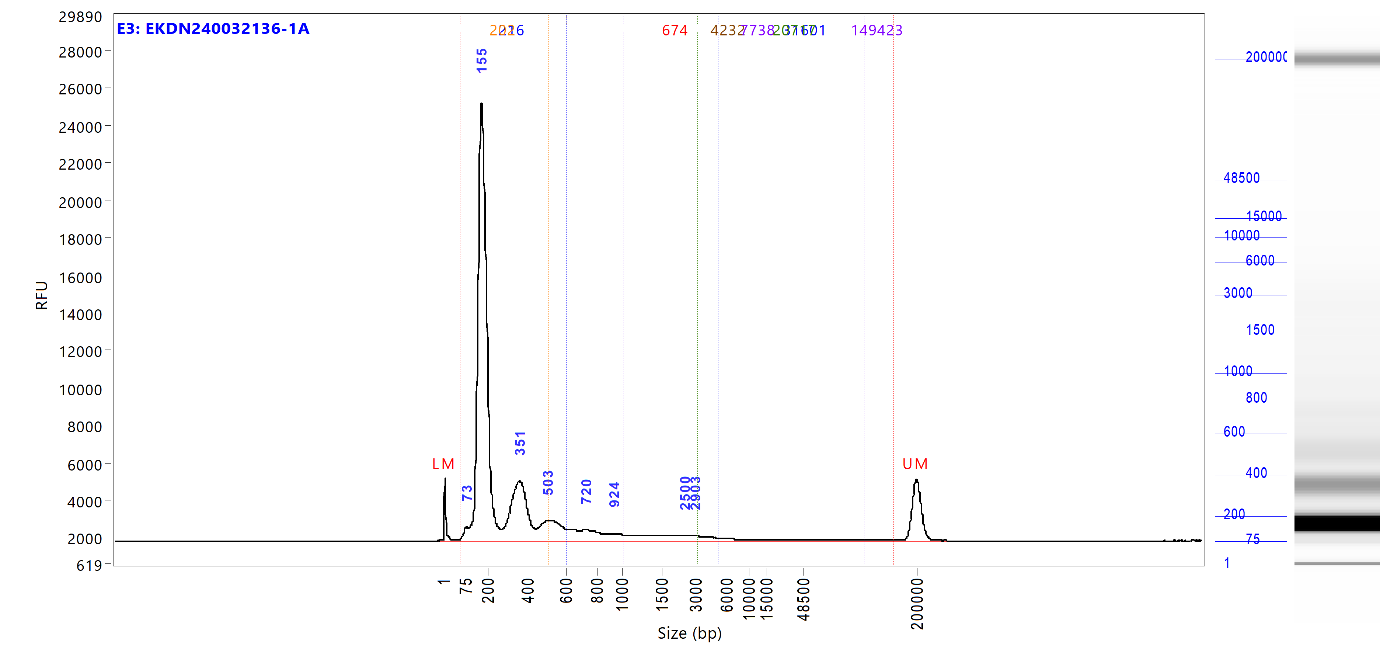


**Supplementary Figure 20. Integrity test results for samples AN23_gill (top, failed eQC), AN23_skin (middle, failed eQC) and AN21_skin (bottom, failed eQC).**


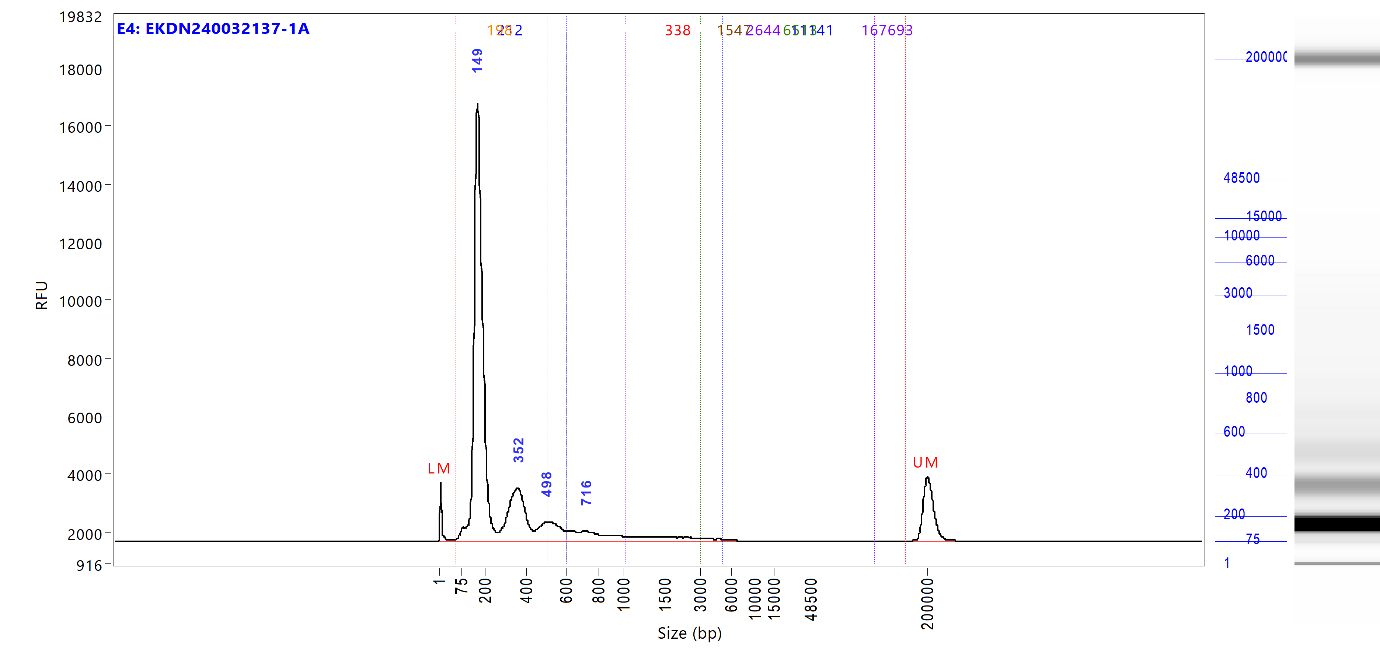

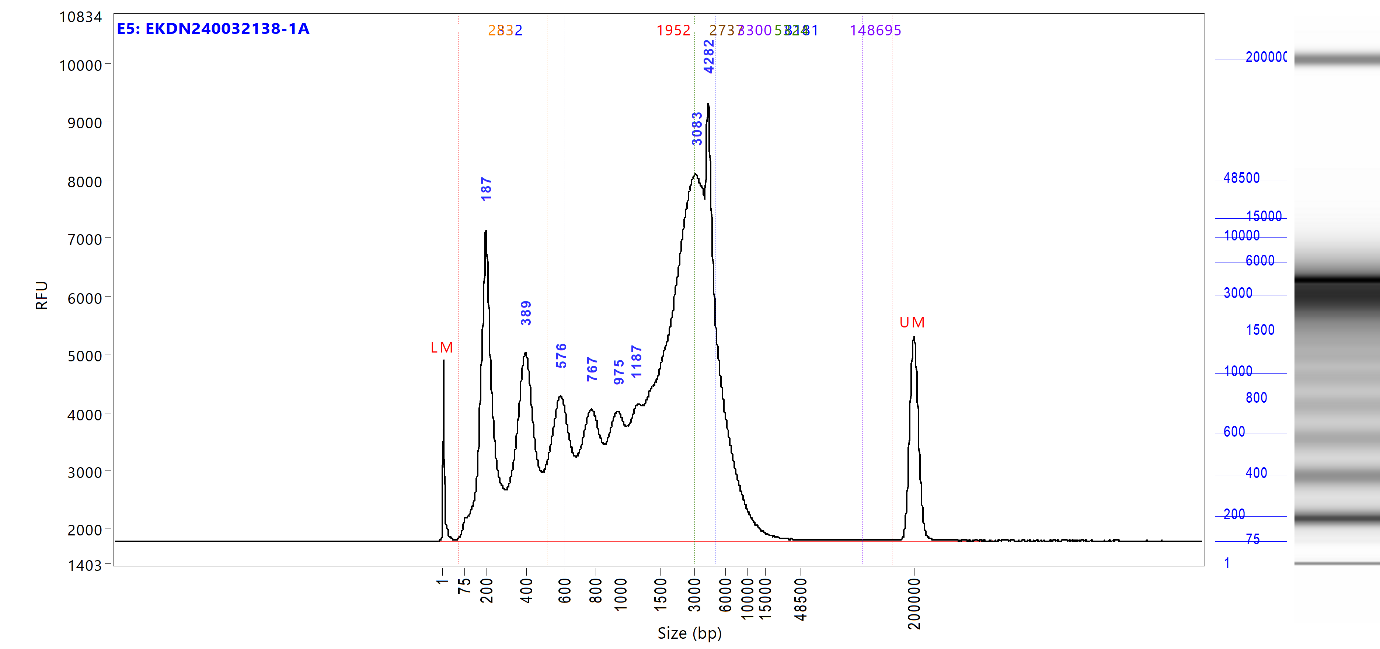

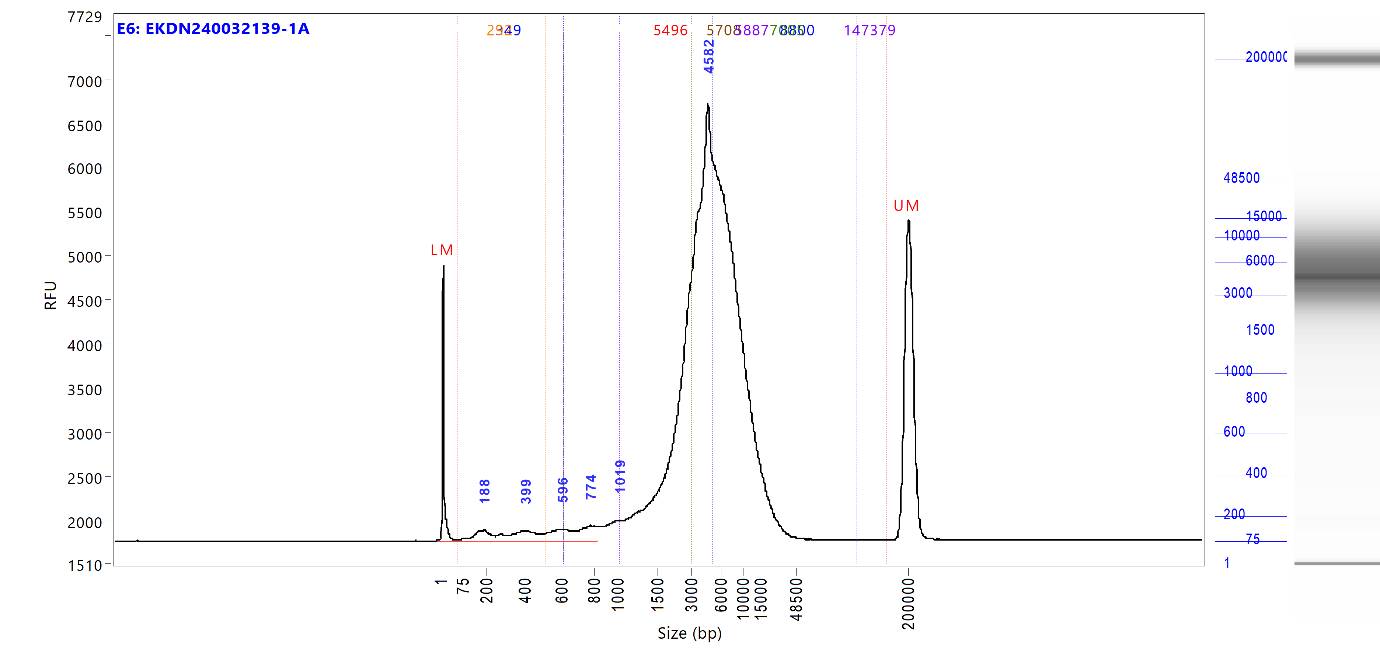


**Supplementary Figure 21. Integrity test results for samples AN19_skin (top, failed eQC), AN18_gill (middle, failed eQC) and AN18_skin (bottom, failed eQC).**


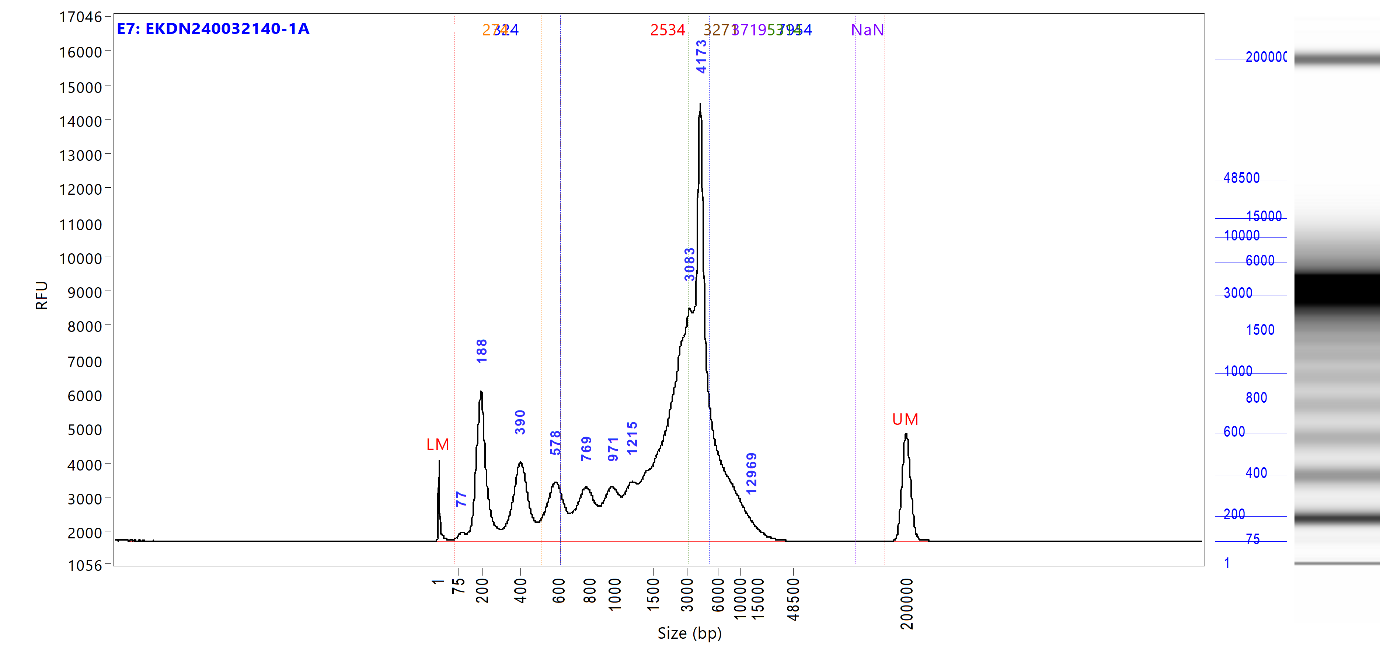

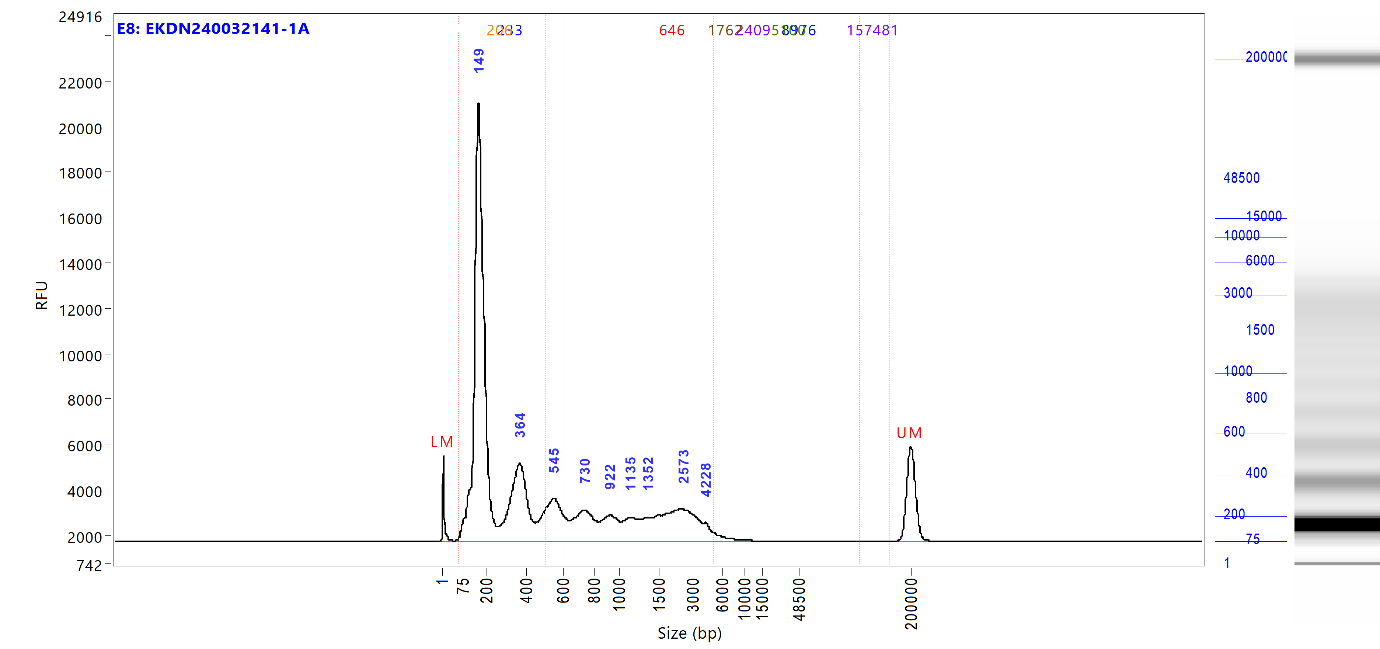

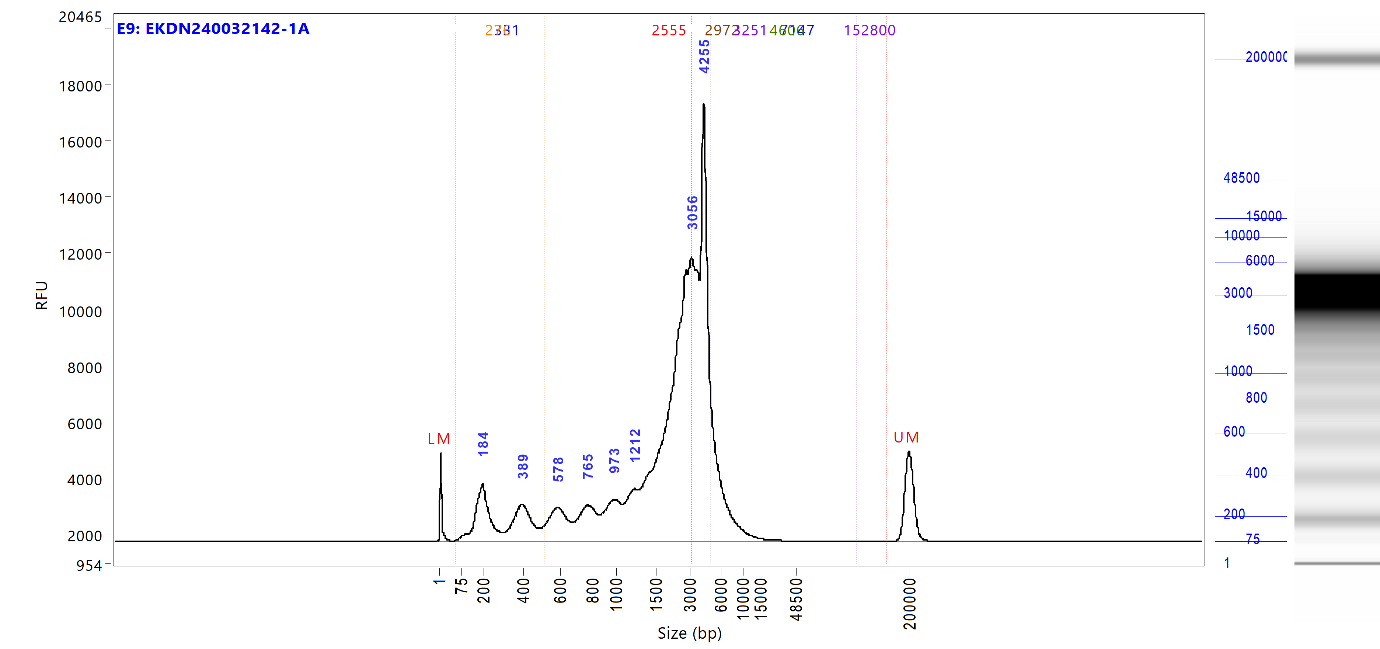


**Supplementary Figure 22. Integrity test results for samples AN17_skin (top, failed eQC), AN15_skin (middle, failed eQC) and AN14_skin (bottom, failed eQC).**


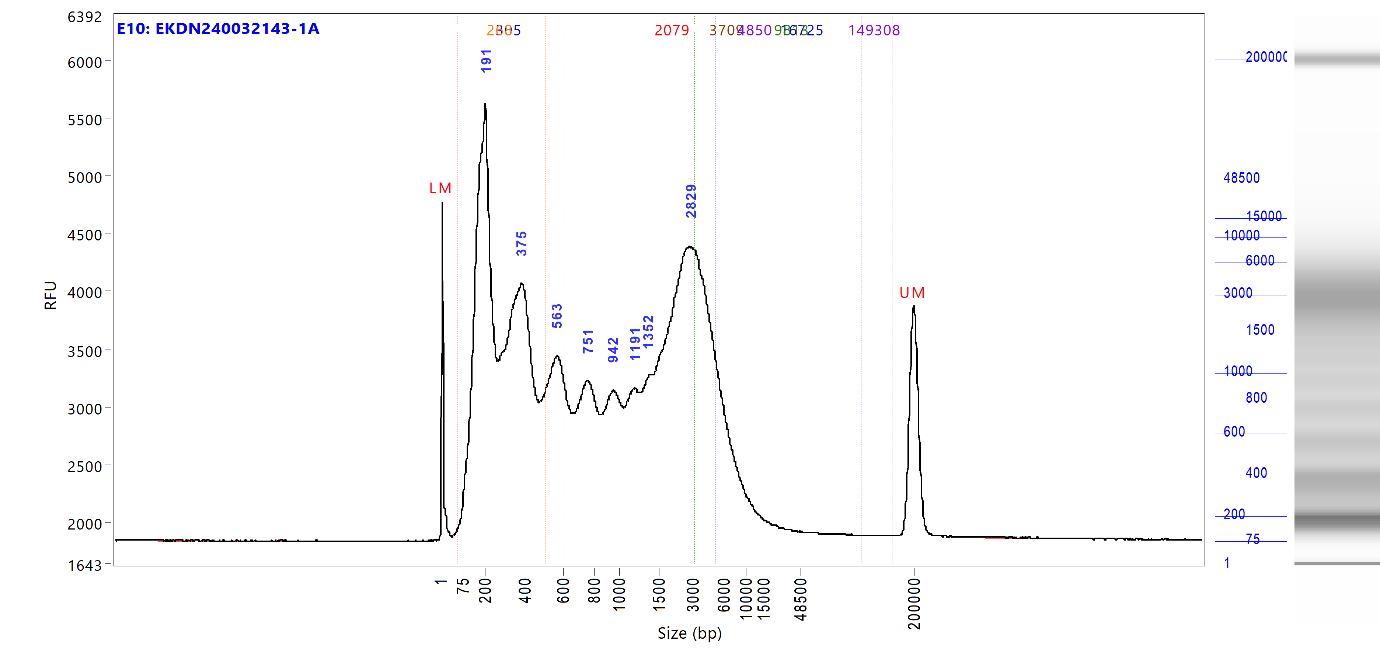

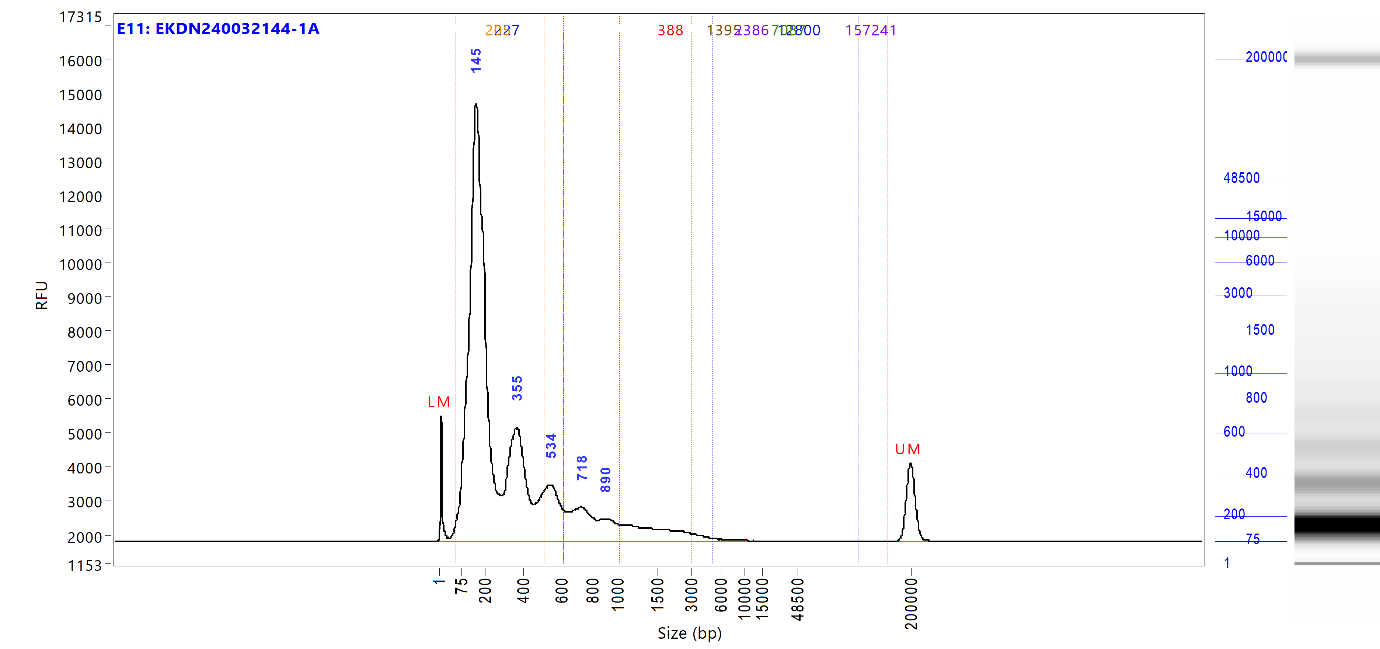

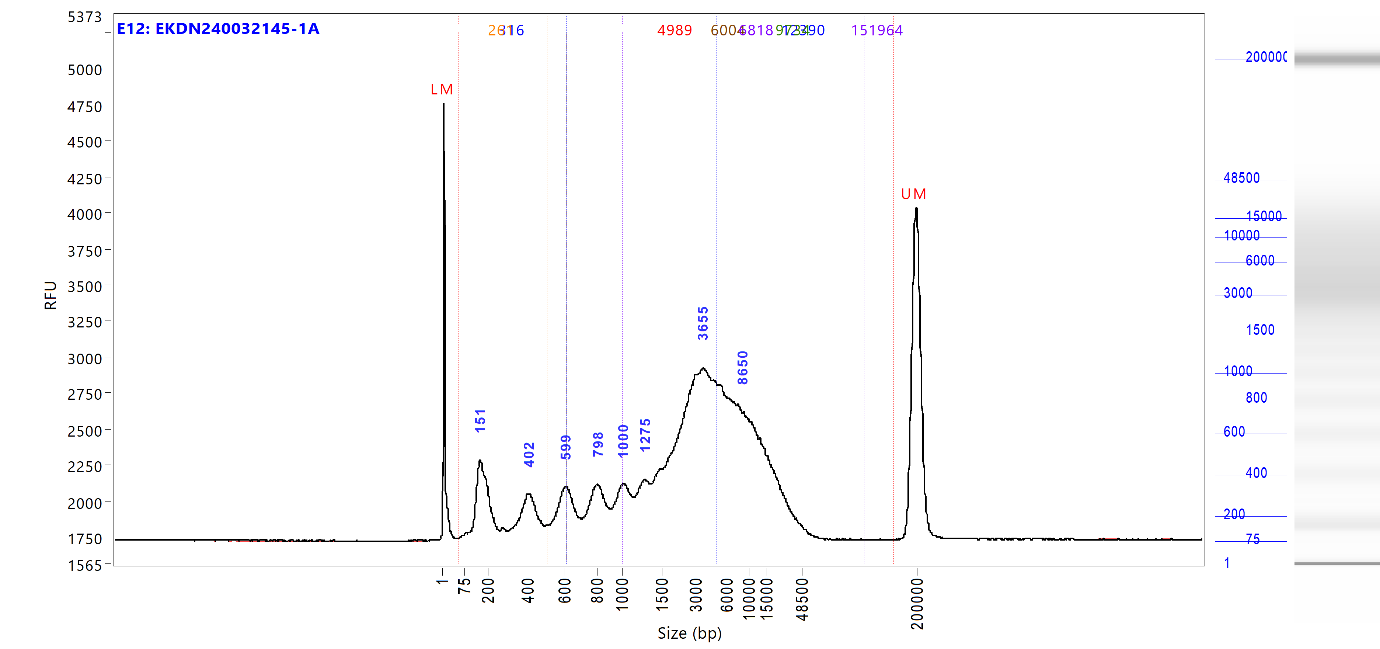


**Supplementary Figure 23. Integrity test results for samples AN13_skin (top, failed eQC), AN12_skin (middle, failed eQC) and AN11_gill (bottom, failed eQC).**


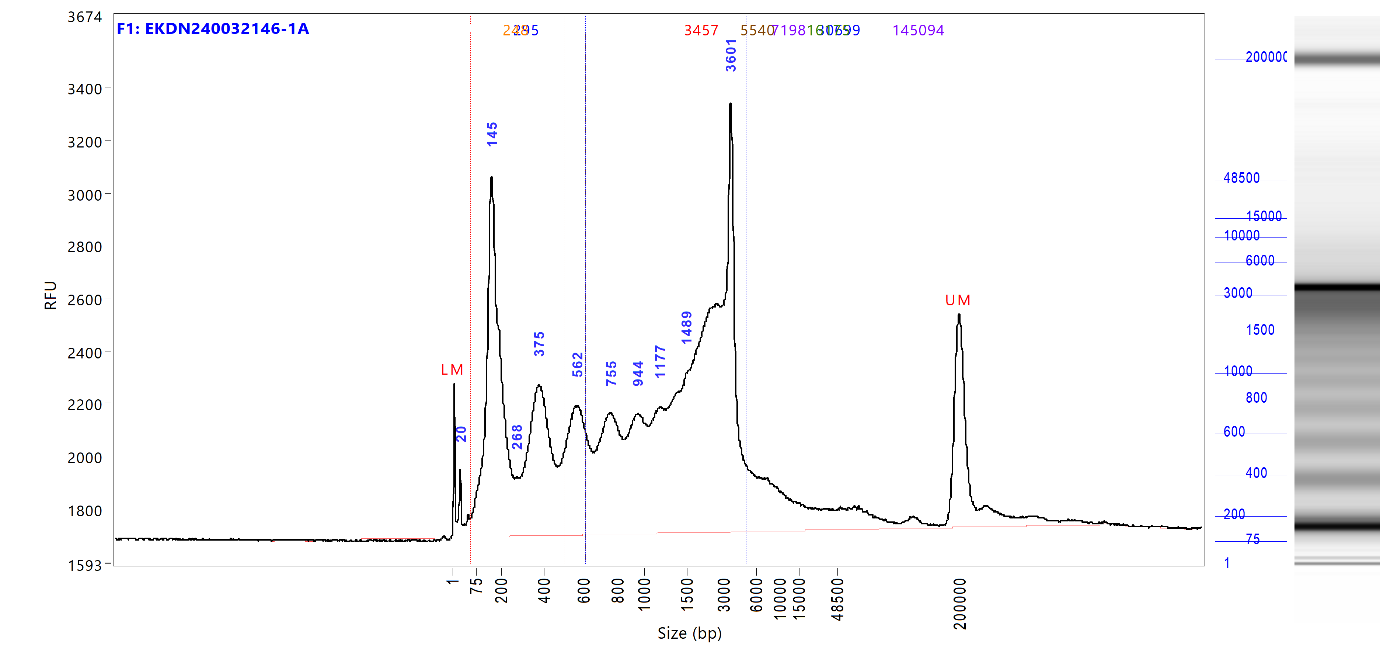

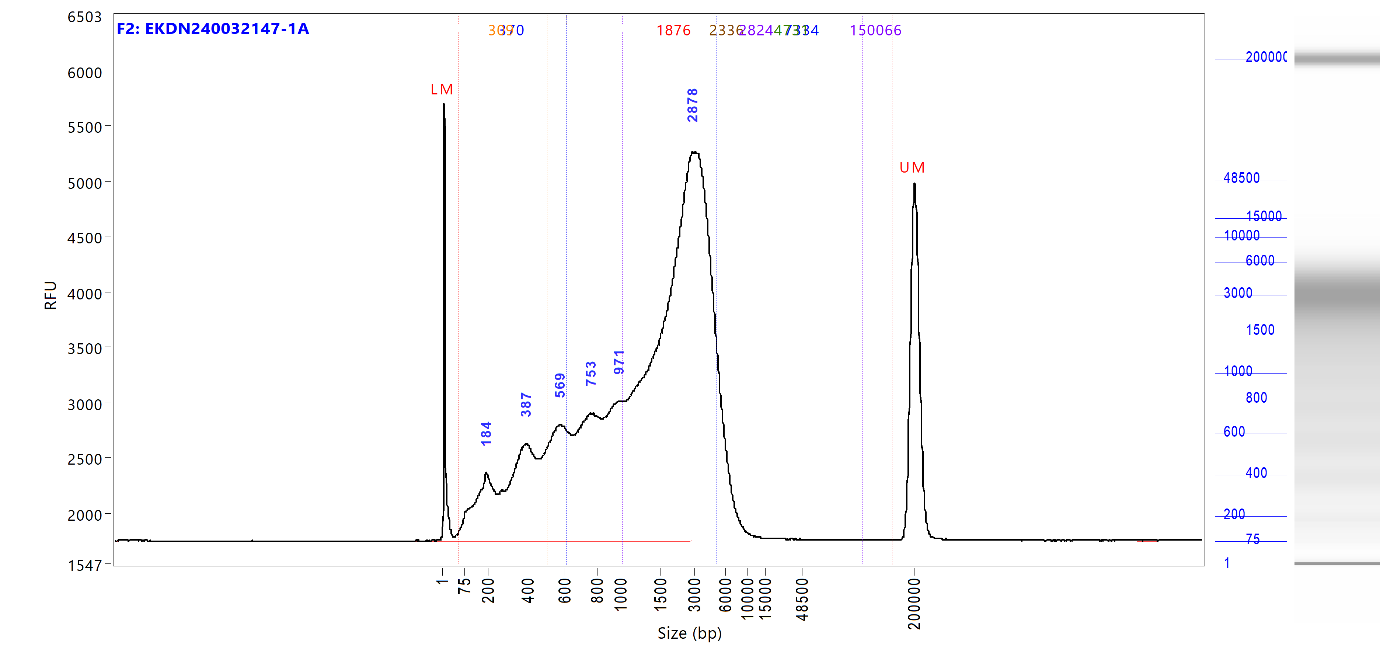

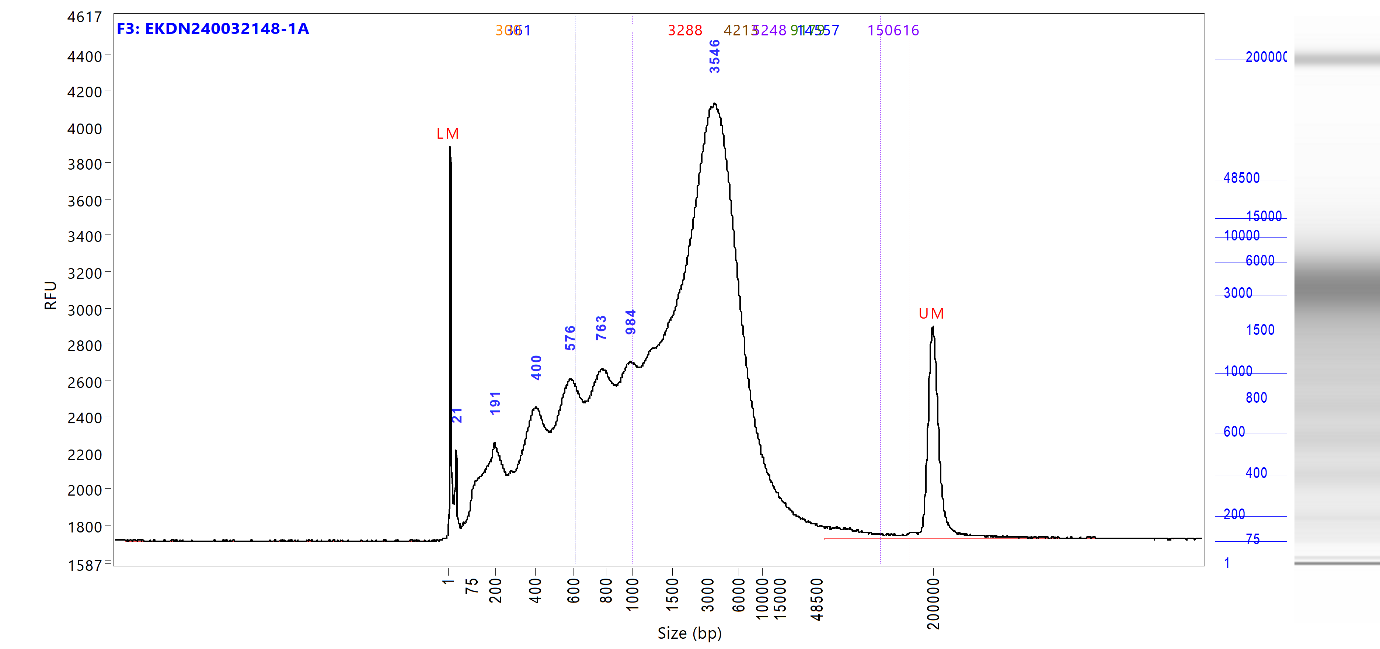


**Supplementary Figure 24. Integrity test results for samples AN11_skin (top, failed eQC), AN10_skin (middle, failed eQC) and AN09_skin (bottom, failed eQC).**


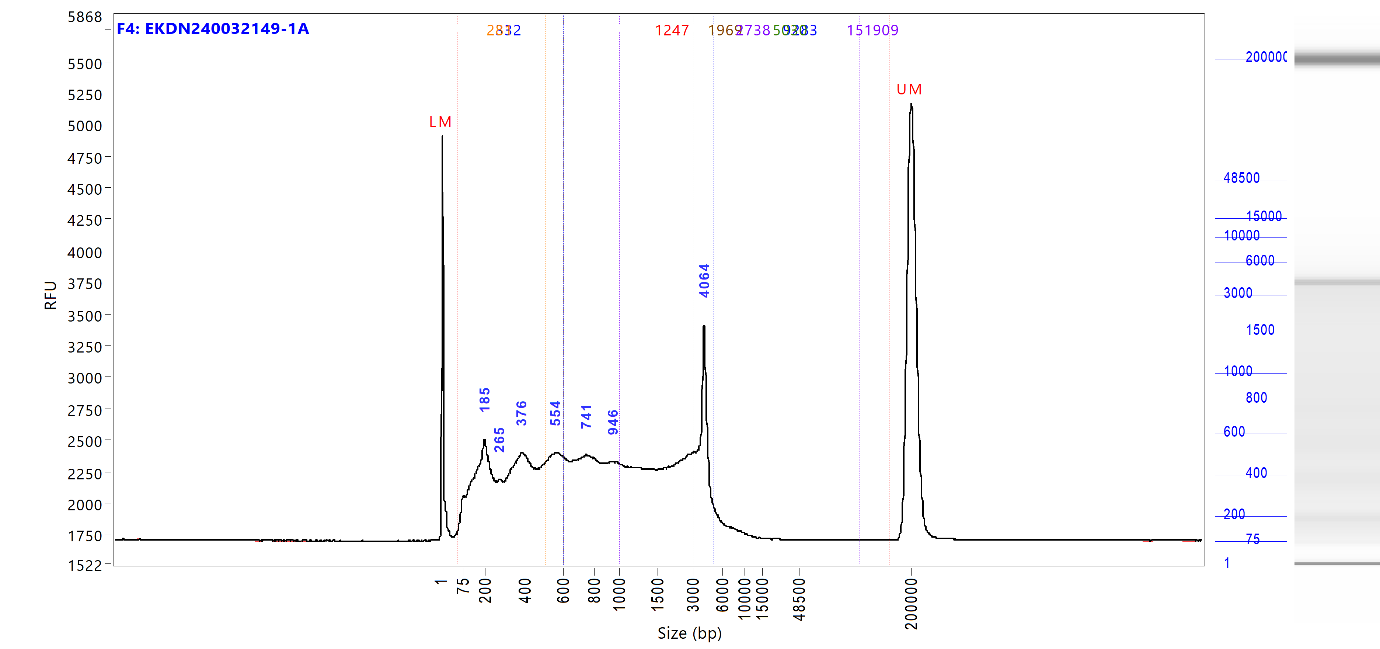

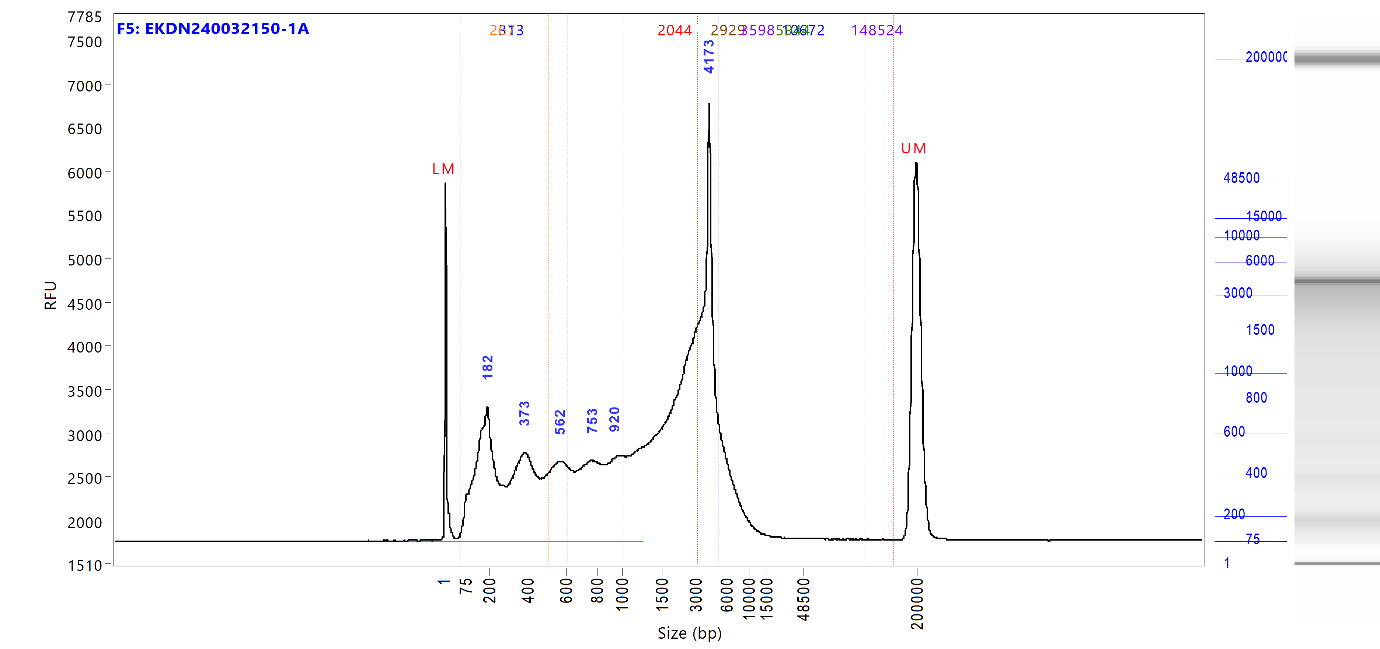

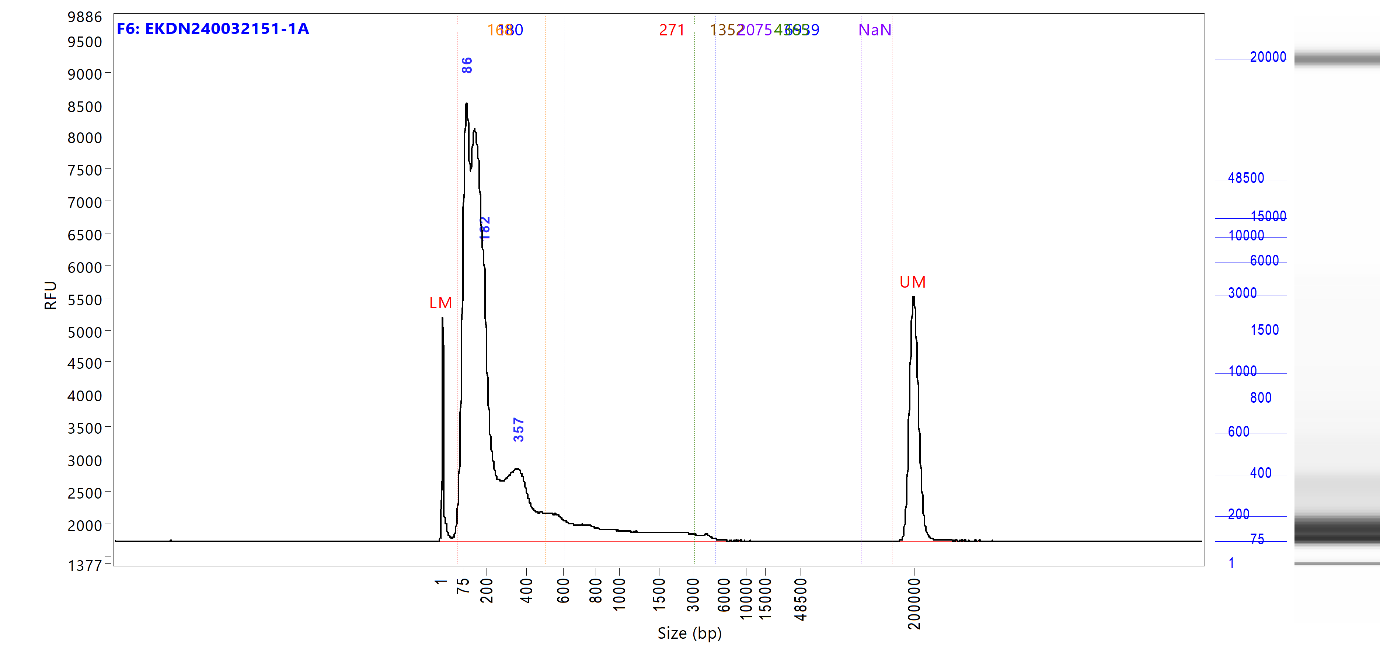


**Supplementary Figure 25. Integrity test results for samples AN07_gill (top, failed eQC), AN07_skin (middle, failed eQC) and AN06_gill (bottom, failed eQC).**


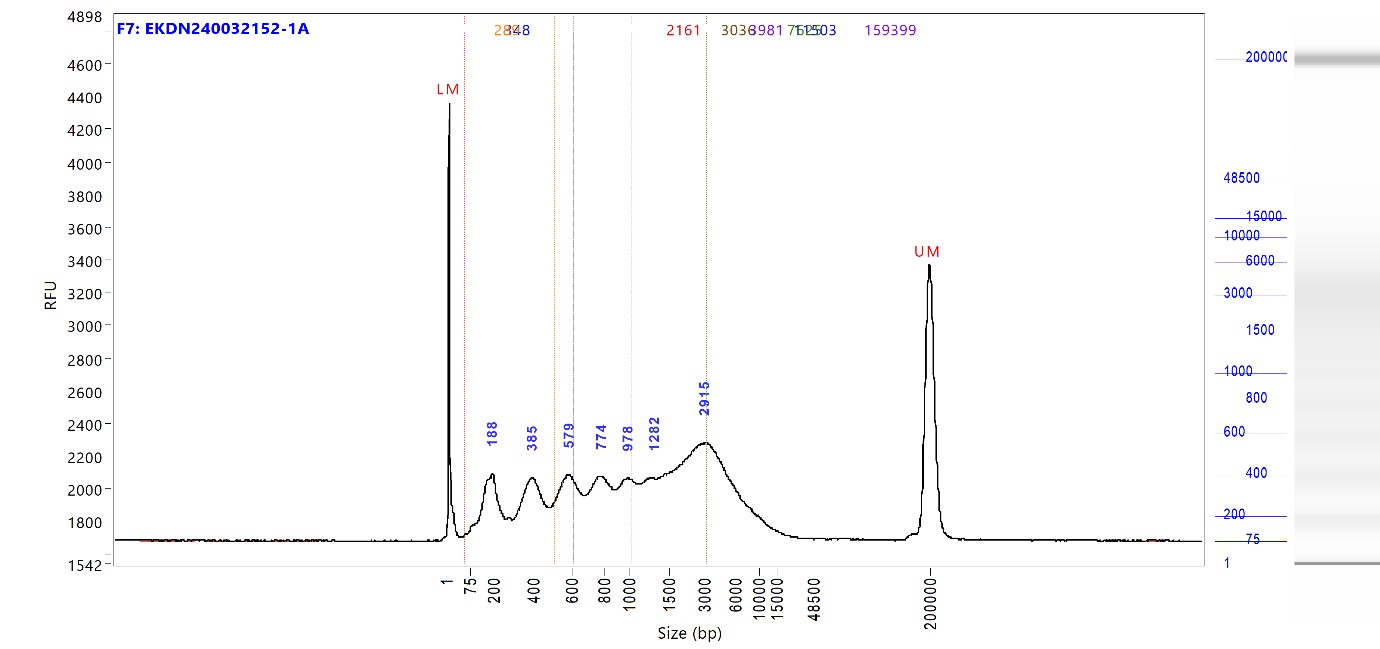

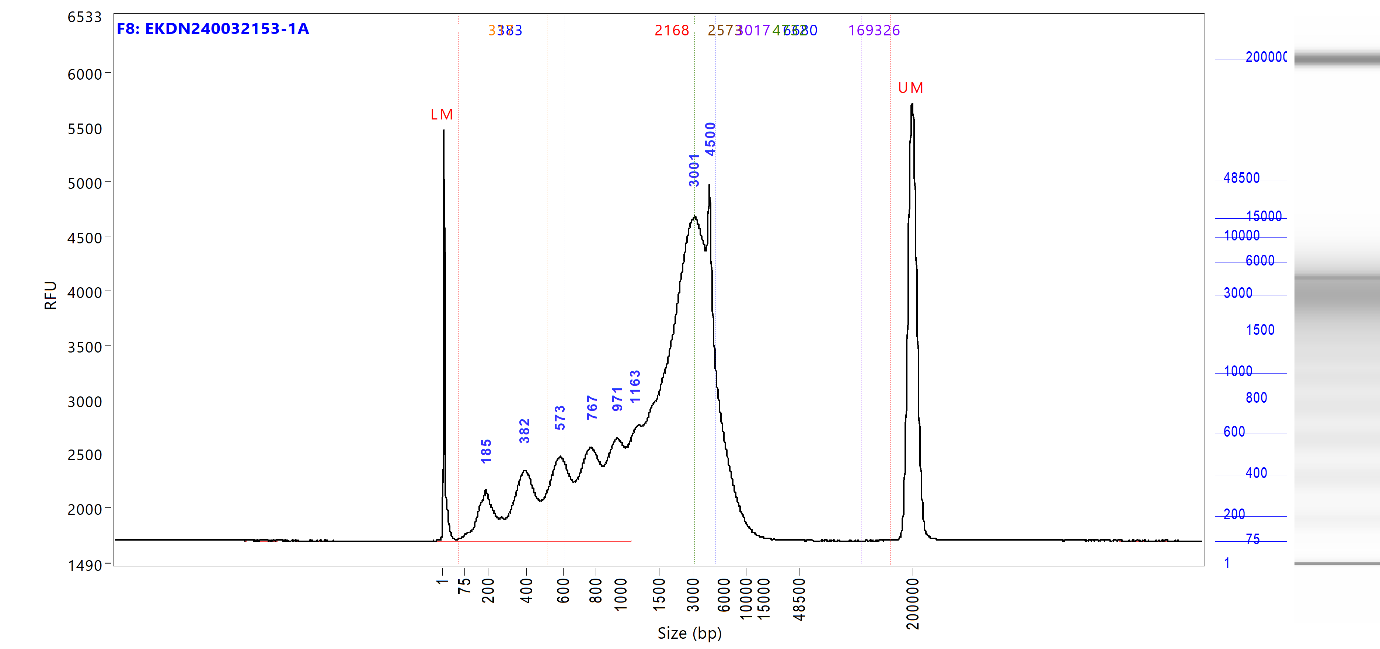

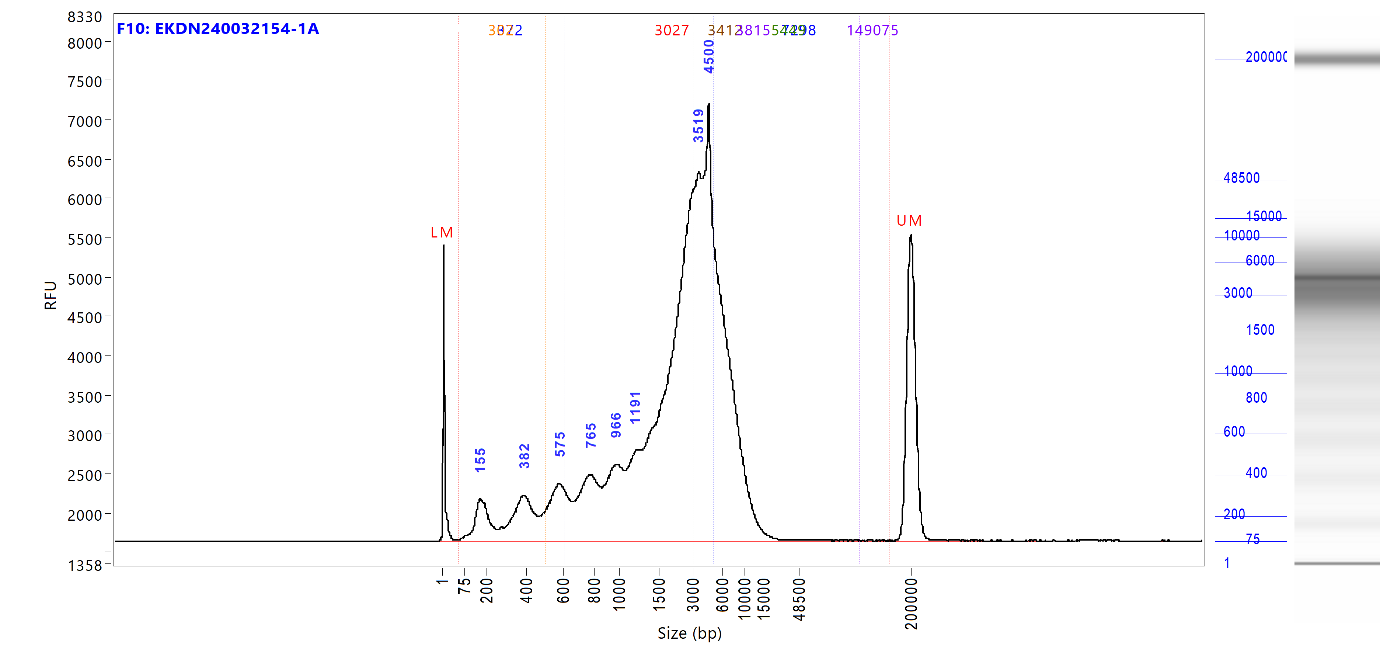


**Supplementary Figure 26. Integrity test results for samples AN05_gill (top, failed eQC), AN05_skin (middle, failed eQC) and AN02_skin (bottom, failed eQC).**


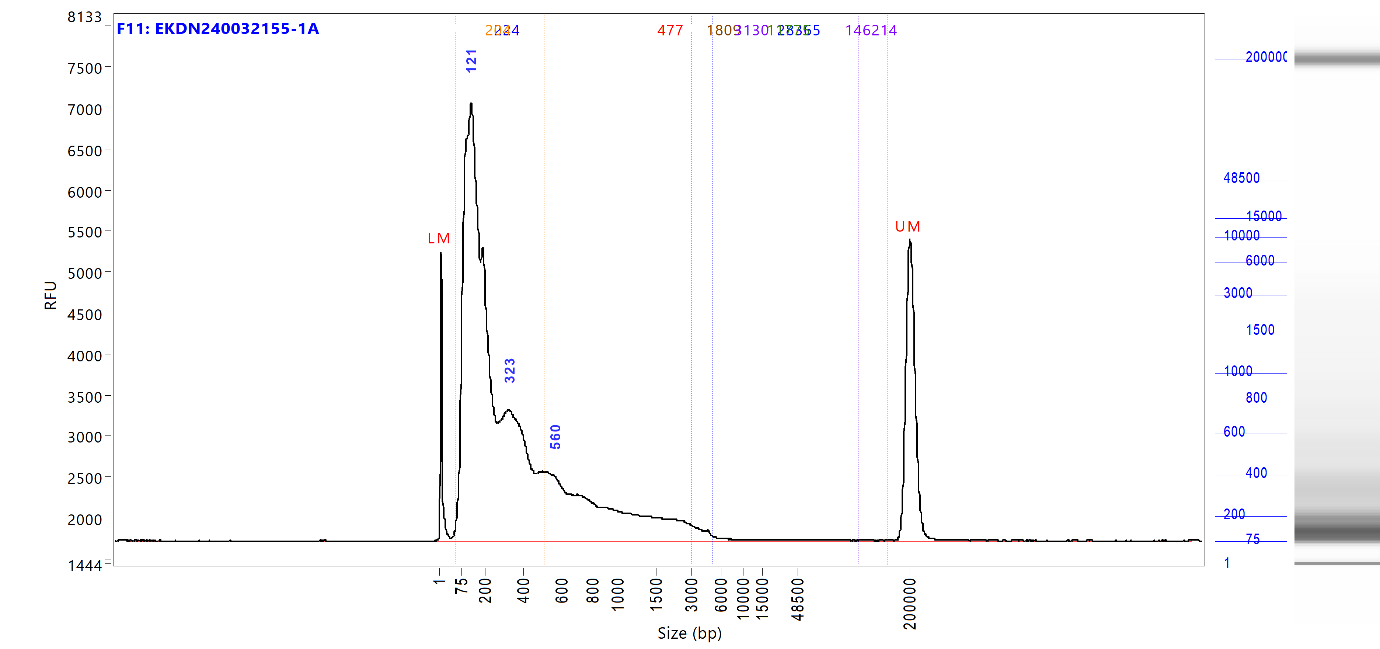

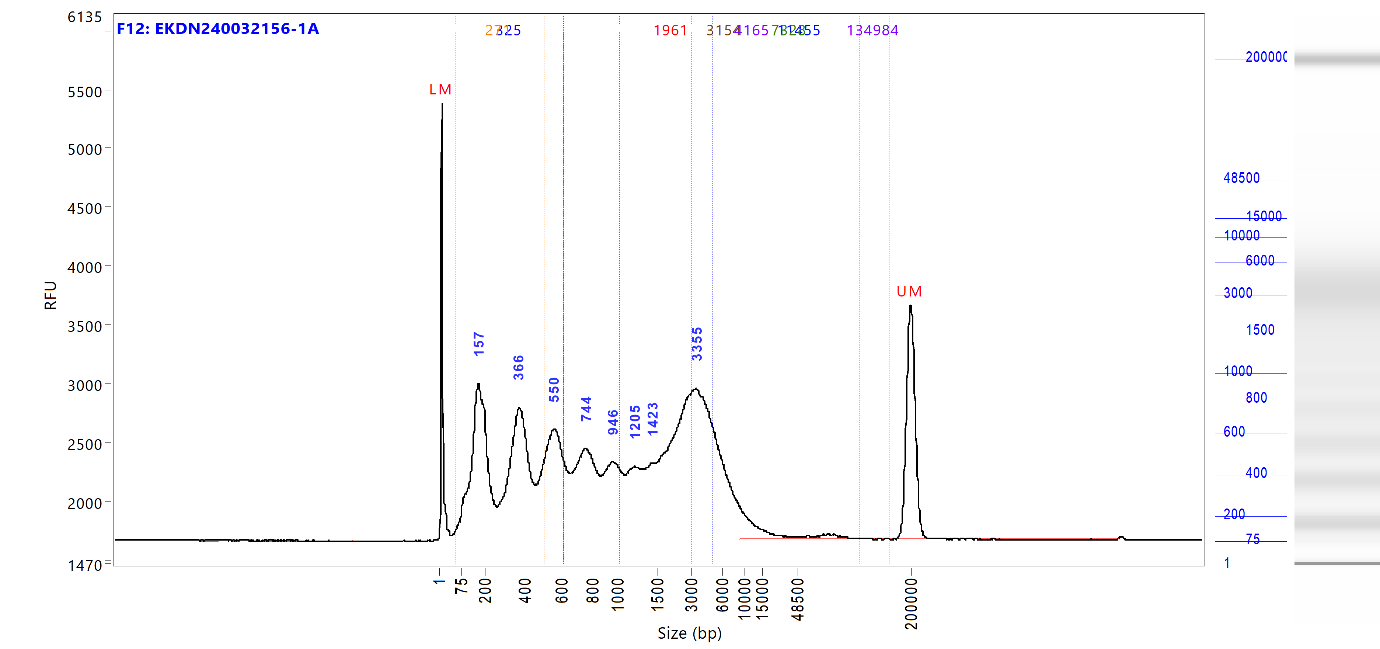

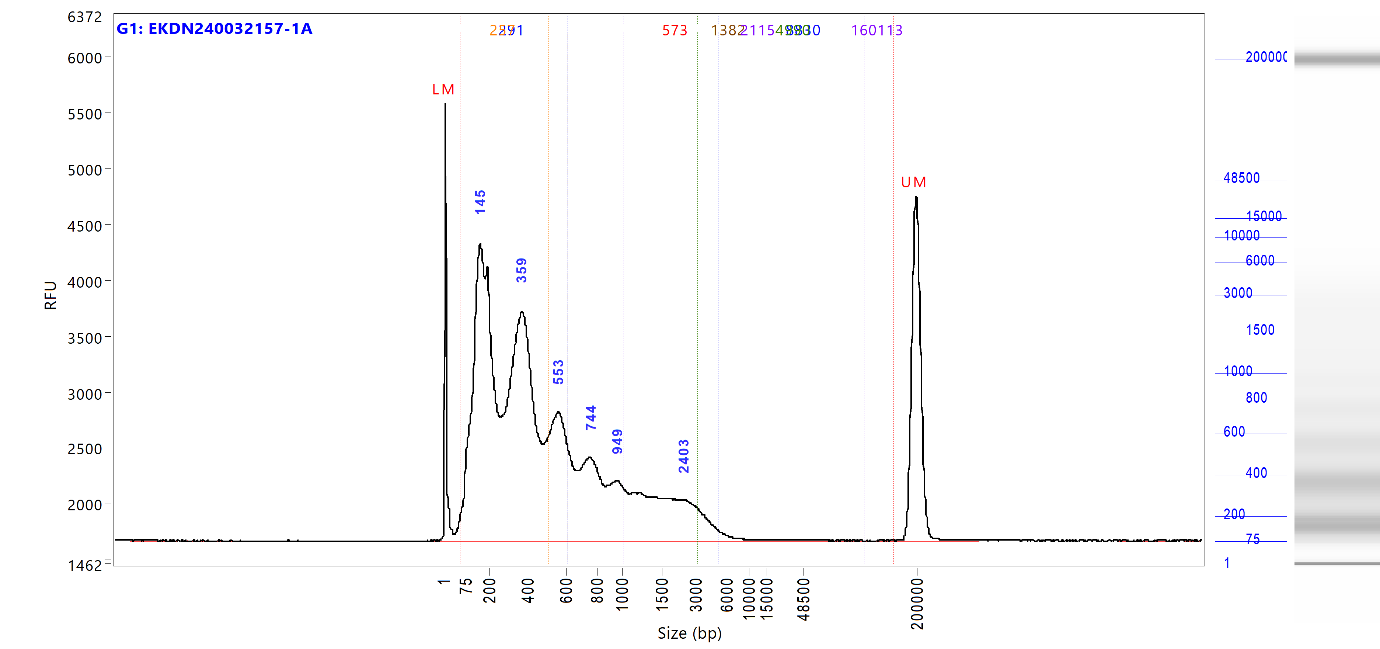


**Supplementary Figure 27. Integrity test results for samples AN00_skin (top, failed eQC), AM98_skin (middle, failed eQC) and AM89_gill (bottom, failed eQC).**


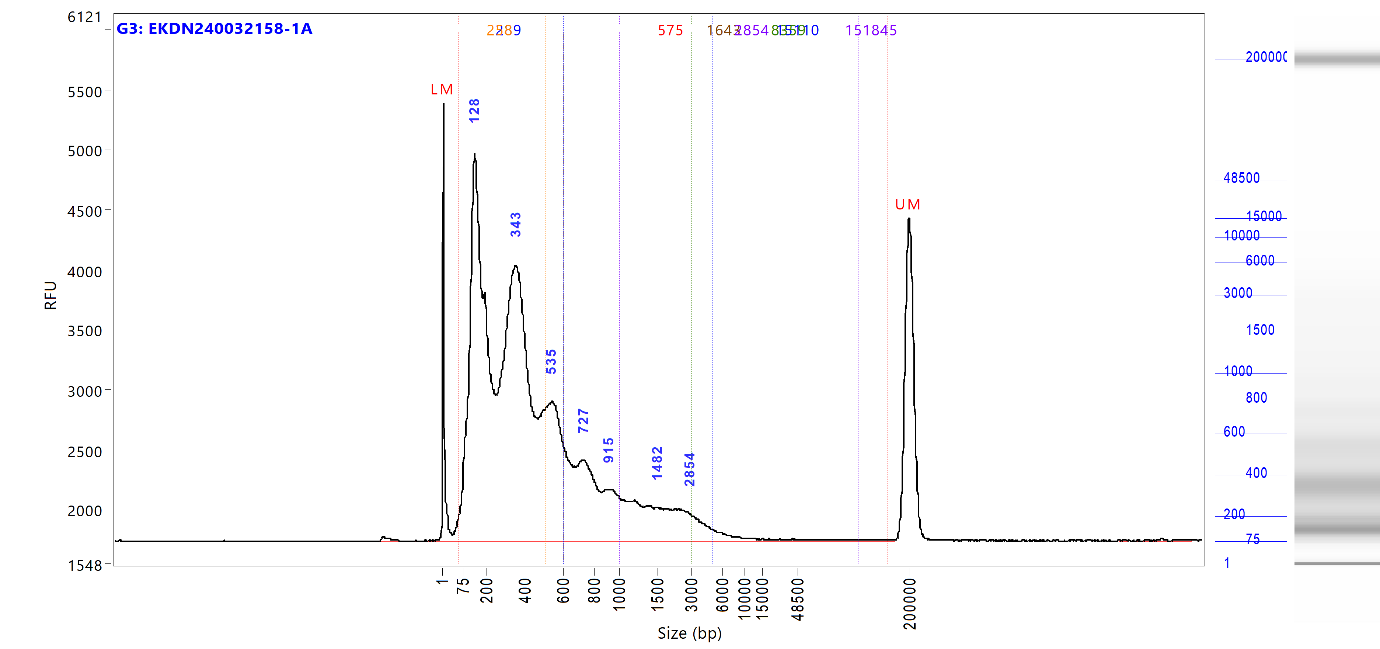

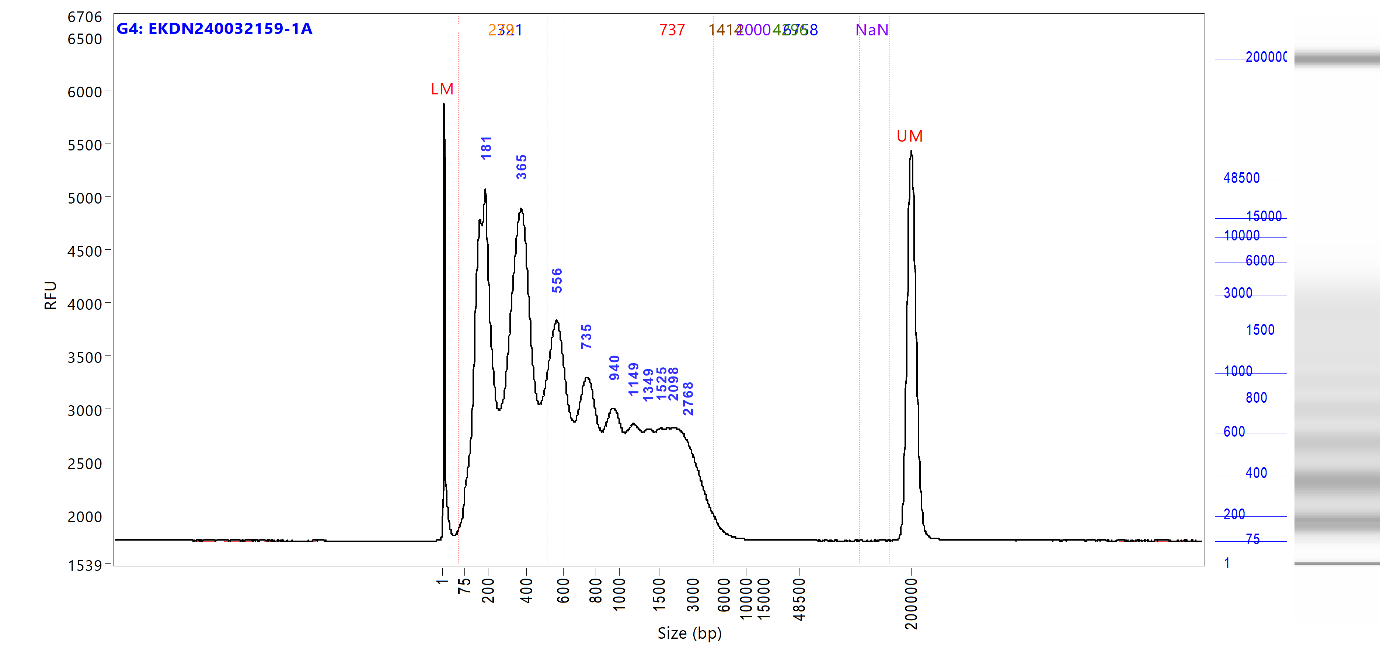


**Supplementary Figure 28. Integrity test results for samples AM97_gill (top, failed eQC), AM95_gill (bottom, failed eQC).**


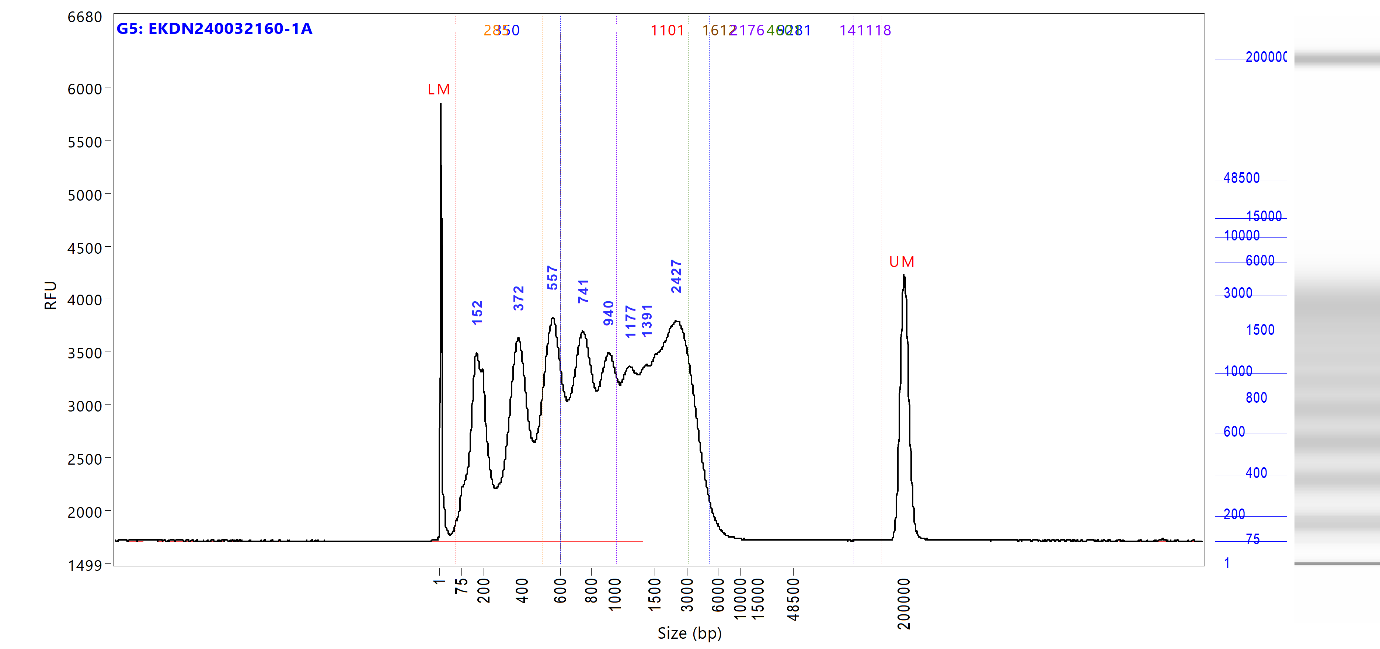

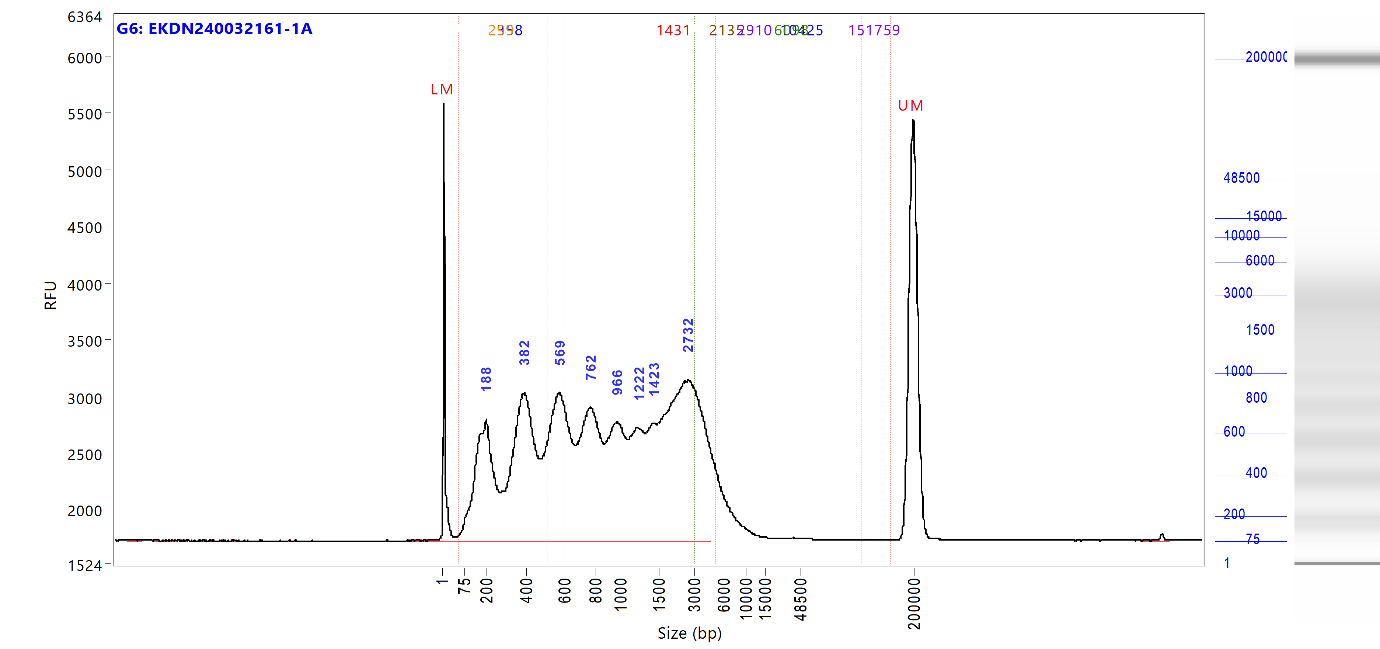


**Supplementary Figure 29. Integrity test results for samples AM94_skin (top, failed eQC), AM92_gill (bottom, failed eQC).**
