# Supplementary material for: Comparison of whole genome sequencing performance from fish swabs and fin clips
Source: BMC Res Notes. 2025 Jan 15;18:15. doi: 10.1186/s13104-024-07075-1 (PMC11734550; doi:10.1186/s13104-024-07075-1)
Supplement: Supplementary file 3 — Supplementary material 3: Table 1. Dunn’s multiple comparisons test results for DNA quality data post DNA extraction. Differences in 260/280 ratio, 260/230 ratio, and final DNA concentration were investigated between fin clips, gill swabs, skin swabs, and skin swabs pre-treated with ATL buffer and/or Proteinase K. A Dunn’s multiple comparisons test was employed for the comparison after significant Kruskal-Wallis test results. [file 13104_2024_7075_MOESM3_ESM.docx]

**Supplementary table 1. Dunn’s multiple comparisons test results for DNA quality data post DNA extraction.** Differences in 260/280 ratio, 260/230 ratio, and final DNA concentration were investigated between fin clips, gill swabs, skin swabs, and skin swabs pre-treated with ATL buffer and/or Proteinase K (skin ATL and skin ProtK). A Dunn’s multiple comparisons test was employed for the comparison after significant Kruskal-Wallis test results.

| **260/280 ratio** | | | |
| --- | --- | --- | --- |
|  | Z | P.unadj | P.adj |
| fin-gill | 3.0390516 | 2.373242e-03 | 2.373242e-02 |
| fin-skin | 4.4701807 | 7.815353e-06 | 7.815353e-05 |
| gill-skin  fin-skin ATL  gill-skin ATL  skin-skin ATL  fin-skin ProtK  gill-skin ProtK  skin-skin ProtK  skin ATL-skin ProtK | 1.3974508  3.6806830  2.2464609  1.5802523  1.1467657  -0.2740281  -0.9402366  -1.8930576 | 1.622780e-01  2.326100e-04  2.467450e-02  1.140491e-01  2.514785e-01  7.840630e-01  3.470962e-01  5.835020e-02 | 1.000000e+00  2.326100e-03  2.467450e-01  1.000000e+00  1.000000e+00  1.000000e+00  1.000000e+00  5.835020e-01 |

| **260/230 ratio** | | | |
| --- | --- | --- | --- |
|  | Z | P.unadj | P.adj |
| fin-gill | 7.5572030 | 4.118289e-14 | 4.118289e-13 |
| fin-skin | 7.8397754 | 4.513531e-15 | 4.513531e-14 |
| gill-skin  fin-skin ATL  gill-skin ATL  skin-skin ATL  fin-skin ProtK  gill-skin ProtK  skin-skin ProtK  skin ATL-skin ProtK | 0.2759227  1.9549607  -1.5733722  -1.7049131  0.5108805  -3.0097995  3.1413405  -1.0788541 | 7.826075e-01  5.058771e-02  1.156327e-01  8.821063e-02  6.094347e-01  2.614202e-03  1.681764e-03  2.806528e-01 | 1.000000e+00  5.058771e-01  1.000000e+00  8.821063e-01  1.000000e+00  2.614202e-02  1.681764e-02  1.000000e+00 |

| **Final DNA concentration** | | | |
| --- | --- | --- | --- |
|  | Z | P.unadj | P.adj |
| fin-gill | 7.1455349 | 8.964611e-13 | 8.964611e-12 |
| fin-skin | 6.3253004 | 2.527404e-10 | 2.527404e-09 |
| gill-skin  fin-skin ATL  gill-skin ATL  skin-skin ATL  fin-skin ProtK  gill-skin ProtK  skin-skin ProtK  skin ATL-skin ProtK | -0.8009321  1.9437057  -1.3929308  -1.0111014  -0.1917772  -3.5170969  -3.1352674  -1.5953924 | 4.231709e-01  5.193095e-02  1.636407e-01  3.119679e-01  8.479167e-01  4.362946e-04  1.716975e-03  1.106245e-01 | 1.000000e+00  5.193095e-01  1.000000e+00  1.000000e+00  1.000000e+00  4.362946e-03  1.716975e-02  1.000000e+00 |
